# SreA-mediated iron regulation in *Aspergillus fumigatus*

**DOI:** 10.1111/j.1365-2958.2008.06376.x

**Published:** 2008-08-21

**Authors:** Markus Schrettl, H Stanley Kim, Martin Eisendle, Claudia Kragl, William C Nierman, Thorsten Heinekamp, Ernst R Werner, Ilse Jacobsen, Paul Illmer, Hyojeong Yi, Axel A Brakhage, Hubertus Haas

**Affiliations:** 1 Division of Molecular Biology/Biocenter, Medical University InnsbruckFritz-Pregl-Str. 3, A-6020 Innsbruck, Austria; 2 Department of Medicine, College of Medicine, Korea UniversityAnam-Dong, Seongbuk-Gu, Seoul 136-705, Korea; 3 J. Craig Venter InstituteRockville, MD 20850, USA; 4 The George Washington University School of Medicine, Department of Biochemistry and Molecular BiologyN.W. Washington, DC 20037, USA; 5 Department of Molecular and Applied Microbiology, Leibniz Institute for Natural Product Research and Infection Biology (HKI), and Friedrich Schiller University JenaBeutenbergstrasse 11a, D-07745 Jena, Germany; 6 Division of Biological Chemistry/Biocenter, Medical University InnsbruckFritz-Pregl-Str. 3, A-6020 Innsbruck, Austria; 7 Department of Microbiology, Leopold-Franzens-University of InnsbruckA-6020 Innsbruck, Austria

## Abstract

*Aspergillus fumigatus*, the most common airborne fungal pathogen of humans, employs two high-affinity iron uptake systems: iron uptake mediated by the extracellular siderophore triacetylfusarinine C and reductive iron assimilation. Furthermore, *A. fumigatus* utilizes two intracellular siderophores, ferricrocin and hydroxyferricrocin, to store iron. Siderophore biosynthesis, which is essential for virulence, is repressed by iron. Here we show that this control is mediated by the GATA factor SreA. During iron-replete conditions, SreA deficiency partially derepressed synthesis of triacetylfusarinine C and uptake of iron resulting in increased cellular accumulation of both iron and ferricrocin. Genome-wide DNA microarray analysis identified 49 genes that are repressed by iron in an SreA-dependent manner. This gene set, termed SreA regulon, includes all known genes involved in iron acquisition, putative novel siderophore biosynthetic genes, and also genes not directly linked to iron metabolism. SreA deficiency also caused upregulation of iron-dependent and antioxidative pathways, probably due to the increased iron content and iron-mediated oxidative stress. Consistently, the *sreA* disruption mutant displayed increased sensitivity to iron, menadion and phleomycin but retained wild-type virulence in a mouse model. As all detrimental effects of *sreA* disruption are restricted to iron-replete conditions these data underscore that *A. fumigatus* faces iron-depleted conditions during infection.

## Introduction

Iron is essential for the vast majority of organisms as it serves as a cofactor in several enzymatic reactions and as catalyst in electron transport systems. However, an overabundance or incorrect storage of iron causes oxidative damage of macromolecules and cell membranes as this metal can act as a catalyst in the formation of highly reactive oxygen species through Haber-Weiss/Fenton chemistry (Haber and Weiss, 1934; Halliwell and Gutteridge, 1984). Therefore, cellular iron homeostasis is designed to tightly regulate the iron supply, while, at the same time, preventing its excess accumulation and cell damaging capacity. Despite its general abundance, the bioavailability of iron is very limited due to oxidation by atmospheric oxygen into sparingly soluble ferric oxyhydroxides. Therefore, all iron-dependent organisms evolved tightly regulated iron acquisition strategies. Moreover, the mammalian defence system against microbial infection includes iron-withholding mechanisms to deny invading microorganism's access to free iron *in vivo* (Weinberg, 1993, 1999; Weiss, 2002; Fluckinger *et al*., 2004). Consequently, the control over access to iron is one of the central battlefields deciding the fate of an infection. Furthermore, iron availability can serve as a regulatory signal not only for expression of genes directly involved in iron uptake and storage but also for other virulence determinants in many prokaryotic and eukaryotic pathogens (Weinberg, 1999).

*Aspergillus fumigatus* is a typical saprophytic mould, which has become the most common airborne fungal pathogen of humans, causing life-threatening invasive disease especially in immunocompromised patients (Latge, 1999; Marr *et al*., 2002). *A. fumigatus* lacks specific uptake systems for host iron sources such as haem, ferritin or transferrin (Schrettl *et al*., 2004a). However, it employs two high-affinity iron uptake systems, siderophore-assisted iron uptake and reductive iron assimilation, both of which are induced upon iron starvation (Schrettl *et al*., 2004a). The siderophore system became a matter of particular interest as it represents an attractive target for antifungal therapy due to its requirement for virulence of *A. fumigatus* and its lack in mammalian hosts (Schrettl *et al*., 2004a; 2007; Hissen *et al*., 2005). Recently, both extracellular and intracellular siderophores have also been implicated in phytopathogenicity of various ascomycetes (Oide *et al*., 2006; Greenshields *et al*., 2007; Hof *et al*., 2007).

Siderophores are low-molecular-mass, ferric iron-specific chelators (Winkelmann, 1993; Haas, 2003). As its close relative *Aspergillus nidulans*, *A. fumigatus* employs the hydroxamate-type siderophores desferri-triacetylfusarinine C (TAFC) to mobilize extracellular iron and desferri-ferricrocin (FC) to intracellularly store iron in hyphae respectively (Oberegger *et al*., 2001; Schrettl *et al*., 2004a). TAFC is a cyclic tripeptide consisting of three *N*^*2*^-acetyl-*N*^*5*^*-cis*-anhydromevalonyl-*N*^*5*^-hydroxyornithine residues linked by ester bonds and FC is a cyclic hexapeptide with the structure Gly–Ser–Gly–(*N*^*5*^-acetyl-*N*^*5*^-hydroxyornithine)_3_ (Haas, 2003). Notably, for conidial iron storage *A. fumigatus* uses hydroxyferricrocin, a derivative of FC, which serves this purpose in *A. nidulans* (Eisendle *et al*., 2006; Schrettl *et al*., 2007). Subsequent to synthesis and excretion, TAFC solubilizes extracellular iron by chelation. Ferri-TAFC (TAFC^+Fe^) is taken up by specific transporters, which belong to the Siderophore-Iron-Transporter (SIT) family of the major facilitator protein superfamily (Winkelmann, 2001; Haas *et al*., 2003). For release of iron from the siderophore, TAFC^+Fe^ is hydrolysed in the cytoplasm by the esterase EstB and an additional unknown mechanism (Oberegger *et al*., 2001; Kragl *et al*., 2007). TAFC degradation products are excreted (Emery, 1976; Oberegger *et al*., 2001), and the iron is transferred to the metabolic machinery or stored as ferri-FC (FC^+Fe^).

**Fig. 1:**
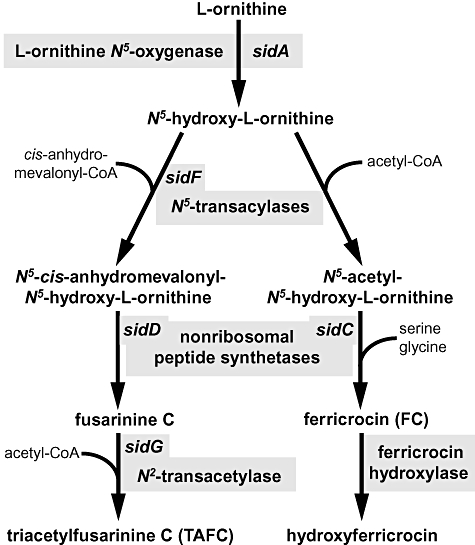
Postulated siderophore biosynthetic pathway of *A. fumigatus*. Biosynthesis of both TAFC and FC starts with *N*^*5*^-hydroxylation of ornithine. Subsequently, the hydroxamate group is formed by the transfer of an acyl group from acyl-coenzyme A (CoA) derivatives to *N*^*5*^-hydroxyornithine. Here the pathways for biosynthesis of TAFC and FC split due to the choice of the acyl group with acetyl for FC and anhydromevalonyl for TAFC. Assembly of the cyclic siderophores fusarinine C and FC is catalysed by different non-ribosomal peptide synthetases (NRPS). TAFC and hydroxyferricrocin are formed by *N*^*2*^-acetylation of fusarinine C and hydroxylation of FC respectively. With exception of the acetyl transferase required for FC biosynthesis all *A. fumigatus* genes encoding respective enzyme activities have been identified and are indicated (Schrettl *et al*., 2004a; 2007).

The postulated biosynthetic pathway according to Plattner and Diekmann (1994) and adapted to the *Aspergillus* system is shown in Fig. 1. The first committed step in the biosynthesis of both TAFC and FC is hydroxylation of ornithine (Fig. 1). Subsequently, the pathways for biosynthesis of TAFC and FC split involving acylation of *N*^*5*^-hydroxyornithine, assembly of siderophore-back bones by non-ribosomal peptide synthetases (NRPS), and derivatization by acetylation or hydroxylation. Five *A. fumigatus* genes encoding respective enzyme activities have been identified (Schrettl *et al*., 2004a; 2007): *sidA* (*N*^*5*^-ornithine-monooxygenase), *sidF* (*N*^*5*^-hydroxyornithine:*cis*-anhydromevalonyl coenzyme A-*N*^*5*^-transacylase), *sidC* (FC NRPS), *sidD* (fusarinine C NRPS) and *sidG* (fusarinine C:acetyl coenzyme A-*N*^*2*^-transacetylase). Elimination of both intra- and extracellular siderophores (Δ*sidA* mutants) results in absolute avirulence of *A. fumigatus* in a mouse model of pulmonary aspergillosis (Eisendle *et al*., 2003; Schrettl *et al*., 2004a). Deficiency in either extracellular (Δ*sidF* or Δ*sidD* mutants) or intracellular siderophores (Δ*sidC* mutants) causes partial attenuation of virulence (Schrettl *et al*., 2007).

In agreement with iron playing an important role in the pathophysiology of *A. fumigatus*, increased bone marrow iron stores represent an independent risk factor for invasive aspergillosis in high-risk patients (Kontoyiannis *et al*., 2007). Moreover, polymorphonuclear leukocytes inhibit growth of *A. fumigatus* conidia by lactoferrin-mediated iron depletion (Zarember *et al*., 2007), and the human body produces proteins able to sequester fungal siderophores (Fluckinger *et al*., 2004). Consistently, the chelator EDTA (which binds iron among other metals) as an adjunct was found to improve the effectiveness of other antifungal agents in a rodent model for invasive pulmonary aspergillosis (Hachem *et al*., 2006).

Iron regulation has been studied in great detail in the fungal prototype *Saccharomyces cerevisiae.* However this yeast is, like *Candida albicans* and *Cryptococcus neoformans*, not able to synthesize siderophores although it can utilize iron bound to siderophores produced by other microbial species (Lesuisse *et al*., 2001; Heymann *et al*., 2002; Hu *et al*., 2002; Philpott, 2006; Winkelmann, 2007). Moreover, in contrast to most fungal species, *S. cerevisiae* lacks an orthologue to SreA. In this yeast, iron regulation is mediated by two paralogous transcriptional activators Aft1p and Aft2p, of which orthologues are missing in most other fungal species (Yamaguchi-Iwai *et al*., 1995; Blaiseau *et al*., 2001; Haas *et al*., 2003). Due to these reasons, the rich literature on iron homeostatic mechanisms in *S. cerevisiae* is not informative for understanding iron metabolism of siderophore-producing fungi. In *A. nidulans*, transcription of the genes encoding orthologues of *A. fumigatus* SidA and SidC as well as the siderophore transporters MirA, MirB and MirC is repressed by iron mediated by the negative-acting GATA-type transcription factor SreA (Haas *et al*., 1999; 2003; Oberegger *et al*., 2001; 2002a). Moreover, interaction of HapX with the CCAAT-binding complex was found to be required for TAFC biosynthesis and for repression of iron-dependent pathways during iron starvation in *A. nidulans* (Hortschansky *et al*., 2007).

To explore the network of iron regulated genes in *A. fumigatus* and to identify further components of the siderophore system, we have generated an *A. fumigatus* mutant lacking the orthologue of *A. nidulans* SreA. Using genome-wide expression profiling, we identified and functionally categorized those genes, whose transcription is regulated by iron availability in an SreA-dependent manner. This study represents the first genome-wide analysis of the influence of iron availability on the transcriptome of a siderophore-producing ascomycete.

## Results and discussion

### Characterization of *A. fumigatus* SreA

Inspection of the annotated genomic sequence of *A. fumigatus* (Nierman *et al*., 2005) revealed the presence of a gene (Afu5g11260) displaying significant identity to *A. nidulans sreA*. Analysis of the *A. fumigatus sreA* cDNA sequence, as described in *Experimental procedures*, revealed the presence of two introns, a 3′-UTR of 377 nt, and two transcription start sites 807 nt and 346 nt upstream of the start codon. The deduced protein of 545 amino acids shows 66% overall identity to the *A. nidulans* orthologue and contains all typical features common to this class of fungal iron-regulatory GATA-transcription factors identified so far in the ascomycetes *Penicillium chrysogenum*, *Neurospora crassa*, *C. albicans* and *Schizosaccharomyces pombe* as well as in the basidiomycetes *Ustilago maydis* and *C. neoformans* (Voisard *et al*., 1993; Haas *et al*., 1997; Zhou *et al*., 1998; Lan *et al*., 2004; Pelletier *et al*., 2005; Jung *et al*., 2006): two GATA-type zinc fingers and an interjacent cysteine-rich region, which was suggested to be involved in iron sensing (Harrison and Marzluf, 2002; Haas, 2003; Pelletier *et al*., 2005). Remarkably, the *C. neoformans* orthologue Cir1 lacks the N-terminal zinc finger motif (Jung *et al*., 2006). An alignment of SreA orthologues from *A. fumigatus*, *A. nidulans*, *C. albicans*, *U. maydis* and *C. neoformans* is shown in Fig. S1 in *Supporting information*. Analysis of the amino acid sequence of SreA using different computer modelling programs such as coils (Lupas *et al*., 1991) and paircoil2 (McDonnell *et al*., 2006) revealed a putative C-terminal amphipathic α-helix (amino acids 508–546, data not shown) suggesting that SreA might self-interact as shown for *S. pombe* Fep1 (Pelletier *et al*., 2005).

### Generation of an *sreA* disruption strain and its genetic complementation

**Fig. 2:**
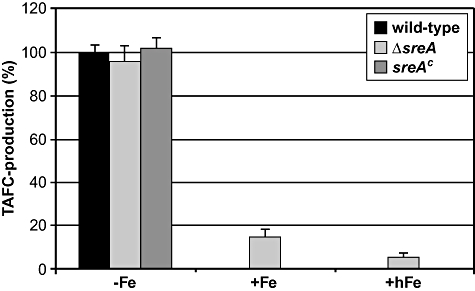
SreA deficiency causes derepression of TAFC production during iron-replete conditions. Quantification of TAFC production after 24 h of growth during iron-depleted (−Fe), iron-replete (+Fe; 10 μM FeSO_4_) and high-iron (+hFe; 1.5 mM FeSO_4_) conditions of Δ*sreA* and *sreA*^*c*^ strains was normalized to that of the wild type during iron-depleted conditions.

**Fig. 3:**
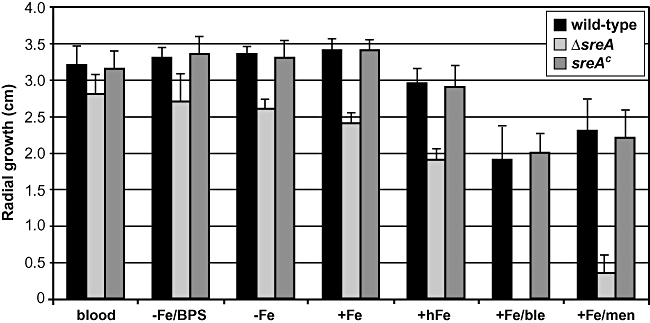
Disruption of *sreA* causes sensitivity to iron, phleomycin and menadion. A total of 10^4^ conidia of the respective strain were point-inoculated on plates containing the indicated growth medium and radial growth was measured after incubation for 48 h at 37°C. −Fe, +Fe, +hFe were as in the legend of Fig. 2; blood was −Fe containing 5% (v/v) sheep blood. BPS was −Fe containing 200 μM bathophenanthroline disulphonic acid; ble and men were +Fe containing 10 μg ml^−1^ phleomycin and 25 μM menadion respectively.

To elucidate the role of *sreA*, we constructed a disruption mutant, termed Δ*sreA*, via replacing the region encoding amino acid residues 1–36 and 321 bp of the 5′-upstream region by the hygromycin B resistance marker in *A. fumigatus* ATCC46645, termed wild type here. The disruption of *sreA* was confirmed by Southern blot and Northern blot analysis (Fig. S2). Complementation of the Δ*sreA* mutant by ectopic integration of a functional *sreA* copy (strain *sreA*^*c*^, Fig. S2) cured all defects (Figs 2 and 3 and data not shown), which demonstrates that the Δ*sreA* mutant phenotype is a direct result of loss of SreA function.

### SreA deficiency causes derepression of TAFC production, and increased cellular accumulation of iron and FC^*+*Fe^

To investigate the role of SreA in extracellular siderophore production, we compared the TAFC content of liquid culture supernatants of wild-type and Δ*sreA* strains after growth for 24 h during iron-depleted, iron-replete (10 μM FeSO_4_) and high-iron (1.5 mM FeSO_4_) conditions by reversed-phase high-performance liquid chromatography (HPLC) analysis. The wild type produced 41 mg of TAFC per gram dry weight during iron-depleted conditions. In contrast TAFC was not detectable during iron-replete and high-iron conditions (Fig. 2). During iron-depleted conditions TAFC production of the Δ*sreA* mutant resembled that of the wild type. However, during iron-replete and high-iron conditions the Δ*sreA* supernatants contained significant amounts of TAFC, 15% and 5%, respectively, compared with iron-depleted conditions. These data indicate that SreA acts as a repressor of TAFC biosynthesis.

Measurement of short-term uptake of ^55^Fe-labelled TAFC^+Fe^, performed as described previously (Oberegger *et al*., 2001), demonstrated that disruption of *sreA* causes about 35% derepression of TAFC^+Fe^ uptake during iron-replete compared with iron-depleted conditions in *A. fumigatus* (data not shown), which is similar to what we found previously in *A. nidulans* (Oberegger *et al*., 2001; Haas *et al*., 2003). Microorganisms take up siderophores only in the ferri- but not the desferri-form. Consequently, during iron-depleted conditions TAFC accumulates in *A. fumigatus* culture supernatants whereas during iron-replete conditions TAFC chelates iron and is subsequently taken up in the Δ*sreA* mutant due to derepressed uptake. Therefore, the quantification of TAFC accumulation underestimates the degree of derepression of TAFC biosynthesis in the Δ*sreA* strain during iron-replete conditions.

To analyse the effect of SreA deficiency on cellular accumulation of siderophores and iron, we compared wild-type and Δ*sreA* strains that were grown for 24 h during iron-depleted, iron-replete and high-iron conditions (Table 1), but used TAFC^+Fe^ instead of FeSO_4_ as iron source because it is a natural iron source of *A. fumigatus* and soluble even in high concentrations (Eisendle *et al*., 2003). FC binds iron stoichiometrically 1:1 and therefore the FC^+Fe^ content directly corresponds to the amount of iron bound to intracellular siderophores. During iron-depleted conditions, the wild type contained about 0.22 μmol of iron and 3.45 μmol of FC per gram dry weight, but did not contain detectable amounts of FC^+Fe^. During iron-replete and high-iron conditions the total cellular iron content was increased 4.5-fold and 7.3-fold, respectively, and FC^+Fe^ constituted in each case about 4% of the total iron content; FC was not detected. The Δ*sreA* mutant did not show any significant difference to the wild type under iron-depleted conditions with respect to FC and total iron content. However, *sreA* disruption caused a 3.5-fold increased total iron content paralleled by a 10.3-fold increased FC^+Fe^ content during iron-replete conditions. During high-iron conditions *sreA* deficiency increased the contents in total iron and FC^+Fe^ 8.8-fold and 19.7-fold respectively. Remarkably, the ratio of FC^+Fe^ to total iron decreased from 12% to 8% in the Δ*sreA* mutant comparing iron-replete and high-iron conditions. The latter indicates that iron is stored to an increasing degree independently of FC^+Fe^ in the Δ*sreA* strain under high-iron conditions, which might be explained by a limited capacity of FC^+Fe^-imparted iron storage or increased activation of FC^+Fe^-independent storage during high-iron conditions. Compared with iron-depleted conditions, Δ*sreA* mycelia contained 54-fold more iron during high-iron conditions, which demonstrates a remarkable wide range in the content of this potentially toxic metal. In contrast to the wild type, Δ*sreA* mycelia were orange coloured during high-iron conditions (data not shown), which is likely to be caused by the huge cellular accumulation of the orange-coloured FC^+Fe^ as found for *A. nidulans* Δ*sreA* (Eisendle *et al*., 2006). Similar to *A. nidulans* (Oberegger *et al*., 2001), the FC content during iron-depleted conditions largely exceeds the FC^+Fe^ content during iron-replete conditions in *A. fumigatus*, here about 86-fold (Table 1). At first sight, production of an iron storage compound during iron starvation appears paradoxical. Possible explanations are that this mode of regulation represents a proactive mechanism to cope with imported iron or that FC also optimizes intracellular iron transfer. In agreement with the latter possibility, inactivation of FC biosynthesis results in decreased growth and conidiation rates during iron starvation as well as an increased iron content during iron-replete conditions in both *A. nidulans* and *A. fumigatus* (Eisendle *et al*., 2003; 2006; Schrettl *et al*., 2007).

**Table 1:** Contents in FC, FC^+Fe^, and total iron of *A. fumigatus* wild-type and Δ*sreA* strains during iron-depleted (−Fe), iron-replete (10 μM TAFC^+Fe^) and high-iron (100 μM TAFC^+Fe^) conditions.

Strain	Growth condition	FC (μmol g^−1^ DW)	FC^+Fe^ (μmol g^−1^ DW)	Total iron (μmol g^−1^ DW)	FC^+Fe^/total iron
Wild type	−Fe	3.45 ± 0.37	0.00	0.22 ± 0.02	–
	10 μM TAFC^+Fe^	0.00	0.04 ± 0.01	1.0 ± 0.08	0.04
	100 μM TAFC^+Fe^	0.00	0.06 ± 0.01	1.60 ± 0.20	0.04
Δ*sreA*	−Fe	3.52 ± 0.37	0.00	0.26 ± 0.03	–
	10 μM TAFC^+Fe^	0.00	0.41 ± 0.01	3.46 ± 0.42	0.12
	100 μM TAFC^+Fe^	0.00	1.18 ± 0.02	14.09 ± 0.99	0.08

The data represent the means ± standard deviations of results from three independent measurements.

DW, dry weight.

Taken together, these data show that SreA deficiency causes in response to increased iron availability an increase of the total iron content accompanied by an increase of the FC^+Fe^ content indicating that SreA is a repressor of iron uptake and that FC^+Fe^ serves as an iron storage compound. The fact that TAFC^+Fe^ uptake was only partially derepressed during iron-replete conditions in the Δ*sreA* mutant indicates the presence of further iron-regulatory mechanisms in *A. fumigatus* as suggested for *A. nidulans* (Oberegger *et al*., 2001). In contrast to TAFC^+Fe^ uptake, accumulation of FC^+Fe^ was higher during high-iron compared with iron-replete conditions in Δ*sreA*. The opposite effect of increasing iron availability on TAFC^+Fe^ uptake and FC^+Fe^ production is likely an adaptation to the intracellular increase of iron, indicating a protective role of FC against iron toxicity. These data also demonstrate that regulation of synthesis of TAFC and FC is not completely coupled and suggest an SreA-independent regulation of FC production. This regulation might be part of the antioxidative stress response as FC^+Fe^ accumulation is responsive to oxidative stress in the wild type, supported by the observation that the addition of paraquat or hydrogen peroxide to a final concentration of 2.0 mM increased the FC^+Fe^ content during iron-replete conditions 2.4-fold and 1.6-fold, respectively, whereas no effect on TAFC production was found (data not shown).

### SreA deficiency increases sensitivity to iron and oxidative stress

Determination of the radial growth rate demonstrated that disruption of *sreA* results in a slightly reduced growth rate during conditions of low iron availability, e.g. in blood agar and iron-depleted medium containing the iron-specific chelator bathophenanthroline disulphonate (Fig. 3). An increase of iron availability decreased the growth rate of the Δ*sreA* mutant significantly more than that of the wild type, which indicates increased sensitivity to iron (Fig. 3). Moreover, the Δ*sreA* strain was more sensitive to the iron-activated antibiotic phleomycin (Haas *et al*., 1999), and the redox cycler menadione (Comporti, 1989) (Fig. 3). These data reflect the perturbation of iron homeostasis and iron-mediated oxidative stress, which is likely to be caused by Haber-Weiss/Fenton chemistry due to deregulated iron uptake (Haber and Weiss, 1934; Halliwell and Gutteridge, 1984). SreA does not appear to influence thermotolerance, cell wall integrity and calcium homeostasis as its deficiency did not affect resistance to high temperatures (up to 50°C), CaCl_2_ (up to 400 mM), FK506 (up to 1 μg ml^−1^), Calcofluor (up to 30 μg ml^−1^) or SDS (up to 0.0125%) either during iron-depleted condition or during iron-replete condition (data not shown). In contrast, lack of the SreA orthologue Cir1 reduces the growth rate at 37°C, and alters calcium sensitivity as well as cell wall composition in *C. neoformans* (Jung *et al*., 2006). However, increased sensitivity to amphotericin B [minimum inhibitory concentration (MIC) of 0.75 compared with > 32 μg ml^−1^] and decreased sensitivity to Posaconazole (MIC of 0.5 compared with 0.25 μg ml^−1^) and voriconazole (MIC of 0.094 compared with 0.125 μg ml^−1^) of the Δ*sreA* mutant compared with the wild-type strain during high-iron conditions (data not shown) indicate an alteration of the plasma membrane. This phenotype could be an indirect effect, possibly due to increased iron accumulation or oxidative stress. SreA deficiency had no effect on resistance to azoles and amphotericin B during iron-depleted growth (data not shown).

### Influence of iron and SreA on expression of selected genes involved in iron uptake and oxidative stress detoxification

To analyse the impact of SreA on gene expression, wild-type and Δ*sreA* strains were grown under iron-depleted conditions for 12 h. Subsequently, iron was added to a final concentration of 10 μM and the cultures were further incubated throughout a time-course. RNA for Northern blot analysis and genome-wide microarray-based expression profiling was isolated at time points 0, 10, 30, 60, 120 and 240 min. We chose this shift experiment starting with iron-depleted conditions (rather than comparing steady-state iron-depleted and iron-replete conditions) to avoid indirect effects as wild-type and Δ*sreA* strains show significant physiological differences (e.g. iron content and oxidative stress sensitivity) during steady-state growth in iron-replete but not iron-depleted conditions. Moreover, this approach allows insight into the dynamics of gene regulation.

**Fig. 4:**
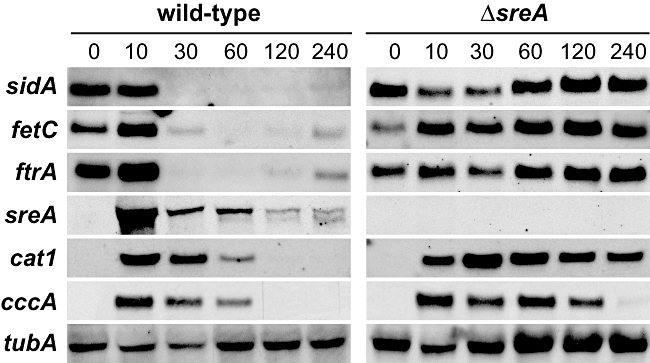
SreA deficiency results in deregulation of iron-regulated genes. *A. fumigatus* wild-type and Δ*sreA* strains were grown in shake flasks for 12 h under iron-depleted conditions at 37°C. Subsequently, FeSO_4_ was added to a final concentration of 10 μM and incubated for another 4 h. Total RNA was isolated at 0, 10, 30, 60, 120 and 240 min after addition of iron and subjected to Northern blot analysis of *sidA* (siderophore biosynthesis), *ftrA* and *fetC* (reductive iron assimilation), *sreA* (iron regulator), *cat1* (haem-containing catalase) and *cccA* (vacuolar iron storage). As a control blots were hybridized with the β-tubulin-encoding *tubA*.

In a first step we investigated the expression of selected genes known to be iron regulated by Northern analysis (Fig. 4). In *A. nidulans*, two different classes of genes that respond inversely to iron availability have been identified (Oberegger *et al*., 2002b; Hortschansky *et al*., 2007): genes repressed by iron (iron acquisition systems) and genes induced by iron (iron-dependent proteins/pathways, e.g. SreA and catalase B).

Iron permease-encoding *ftrA* and ferroxidase-encoding *fetC*, which are both involved in reductive iron assimilation (Schrettl *et al*., 2004a), as well as siderophore-biosynthetic *sidA* were highly expressed during iron-depleted conditions in both *A. fumigatus* wild-type and Δ*sreA* strains (Fig. 4). In the wild type, the transcript levels of all three genes dramatically decreased within 30 min after the addition of iron. After 120 min the expression of all three genes slightly increased, which indicates consumption of the available iron and re-induction of the high-affinity iron uptake systems at the transcriptional level. The derepression of all three genes in the Δ*sreA* mutant after the addition of iron confirms SreA-mediated iron regulation of siderophore biosynthesis (see above) and reveals that SreA also controls reductive iron assimilation.

In contrast to the genes involved in iron acquisition, *sreA* transcripts were not detectable during iron-depleted conditions but addition of iron resulted in the appearance of *sreA* transcripts, 2.9 kb in size, within 10 min in the wild-type strain (Fig. 4). Subsequently, the transcript level steadily decreased. Remarkably, after 60 min, a second transcript with a length of about 2.5 kb appeared. Notably, the appearance of the 2.5 kb *sreA* transcript roughly paralleled the re-induction of high-affinity uptake systems. Both transcript types were also found during steady-state iron-replete conditions (Fig. S3). The two *sreA* transcript types are in accordance with the two transcription start sites found (see above). Consistent with disruption of *sreA*, the Δ*sreA* strain lacked both transcripts. The suppression of *sreA* expression during iron-depleted conditions matches the function of the gene product as a repressor of iron uptake. The rational, if any, for the switch of the transcription start points is not known. Notably, the iron-regulated expression with two transcripts differing in length is conserved in the *A. nidulans* and *S. pombe* orthologues (Haas *et al*., 1999; Pelletier *et al*., 2002; Mercier *et al*., 2008). In contrast, only one transcript type, the level of which does not respond to iron availability, has been found for *sreA* orthologues in *N. crassa* and *U. maydis* (Voisard *et al*., 1993; Zhou *et al*., 1998).

Transcription of mycelial catalase-encoding *cat1*, which confers resistance to oxidative stress in *A. fumigatus* (Paris *et al*., 2003), was induced within 10 min in both wild-type and Δ*sreA* strains (Fig. 4). Subsequent to a maximum at 10 min, the transcript level declined in the wild type but not in the Δ*sreA* mutant, which might reflect increased oxidative stress as suggested by the increased sensitivity to menadion (see above).

Measurement of the contents in total iron and FC^+Fe^ suggested siderophore-independent means of iron storage (Table 1, see above). The *A. fumigatus* genome encodes two putative orthologues (Afu3g09970 and Afu4g12530) to Ccc1p, which mediates vacuolar iron storage in *S. cerevisiae* (Li *et al*., 2001). Afu3g09970 transcripts were detected neither in the wild type nor in the Δ*sreA* strain at any time point (data not shown). In contrast, Afu4g12530 (termed *cccA* here) revealed strong induction by iron and prolonged expression during the time-course in the Δ*sreA* mutant (Fig. 4) indicating that CccA is possibly also involved in vacuolar iron storage. As found previously (Schrettl *et al*., 2004a), the transcript level of β-tubulin-encoding *tubA* is unresponsive to iron availability.

### Transcriptomes during a shift from iron-depleted to iron-replete conditions

To profile the genome-wide expression responses of *A. fumigatus* in a shift from iron-depleted to iron-replete conditions and to identify the genes regulated by SreA, we conducted a microarray analysis with the wild-type and Δ*sreA* strains using the same RNA samples that were used for the Northern blot analysis (see above). RNA samples from each time point (i.e. 0, 10, 30, 60, 120 and 240 min) were compared with that from 0 min (the reference) in hybridizations. The expression profiles were obtained using *A. fumigatus* whole-genome DNA microarrays (Nierman *et al*., 2005). Of the 9516 genes represented on these arrays, 8975 satisfied our quality control standards, and we were able to identify 1147 genes that were differentially expressed (95% confidence level) in the wild-type strain in response to the shift from iron-depleted to iron-replete conditions at one or more time points. These genes were clustered using *k*-means (*k* = 25, Euclidean distance) for similar expression vectors (Table S1, Fig. S4A–C). Clusters 1–13 contain genes that are downregulated in response to the iron addition, while clusters 14–18 contain genes that are upregulated. The genes from clusters 19–25 exhibited various patterns that are both up and down. Most gene expression patterns were more or less similar between the wild type and the Δ*sreA* strain, except genes from clusters 1, 2 and 3 that showed significantly decreased downregulation in the Δ*sreA* strain after addition of iron. Furthermore, genes from clusters 14 and 15 showed significantly increased expression in the Δ*sreA* strain at later time points.

### Genes repressed by SreA

**Fig. 5:**
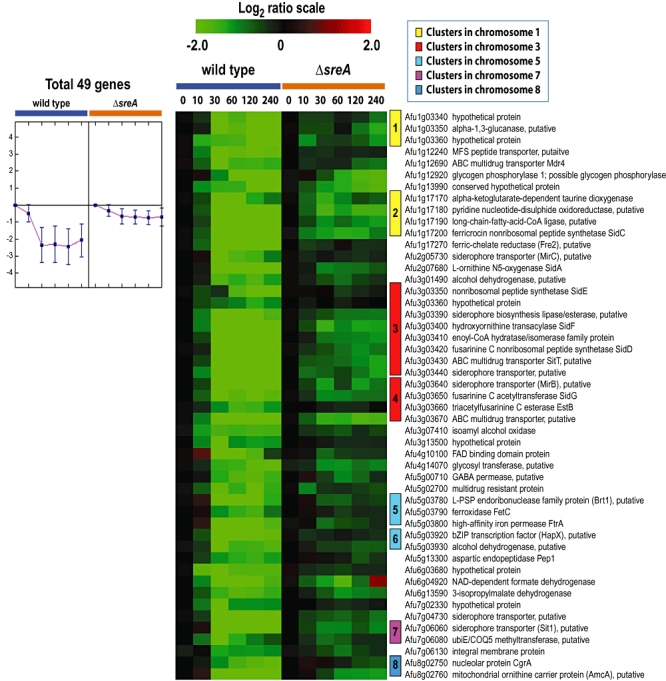
Heatmap representation of gene expression profiles for the putative SreA-responsive genes. Microarray data from the iron concentration shift assay with the wild-type and Δ*sreA* strains are displayed in parallel for comparison. The bar at the top indicates colours corresponding to the range of the observed expression ratios on a log_2_ scale. Genes are displayed in the order of chromosomal localization. Numbers 1–8 in colour-coded boxes, representing gene clusters on chromosomes, denote SreA-Regulated Gene Clusters (SRGCs) found in this study. A graph left to the heatmap displays average ratio values with error bars of the data from each time point.

**Fig. 6:**
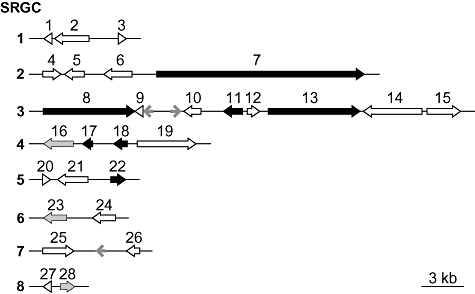
Schematic map of SRGC1–8. Arrows denote the transcriptional orientation of clustered genes numbered according to Table 2. Genes with a characterized role in iron homeostasis in *A. fumigatus* and *A. nidulans* are marked in black and grey respectively. Arrows without numbering represent genes, expression of which was not significantly affected by SreA in the microarray analyses.

As found for selected genes in the Northern analysis (Fig. 4), addition of iron decreased transcript levels of genes from clusters 1–13 in the wild type after 10–30 min (Table S1, Fig. S4A). SreA deficiency caused significantly higher expression of 44 genes of the clusters 1–3, indicating that SreA directly or indirectly regulates expression of these genes. Strikingly, 23 of these genes are organized in eight gene clusters (Fig. 5). We named them SreA-Regulated Gene Cluster (SRGC) 1–8. Five genes (i.e. Afu3g03350, Afu3g03360, Afu3g03660, Afu5g03790, Afu7g06080) from clusters 5, 7 and 11 are located within the SRGC. While these showed only mild SreA-dependent expression patterns, there is high possibility for them to be part of the SreA regulon as genes involved in a common pathway often tend to be in clusters in *A. fumigatus* (Schardl, 2006). Therefore, we present these 49 genes as potential subjects of SreA-mediated regulation (Fig. 5). Six out of these eight gene clusters (SRGC2–4 and SRGC6–8) appear to be related to siderophore metabolism as member genes have previously been implicated in siderophore biosynthesis, uptake or utilization (Fig. 6, Table 2): SRGC2 encodes SidC; SRGC3 encodes SidF, SidD, and a putative siderophore transporter (Afu3g03440); SRGC4 encodes SidG, EstB, and a putative siderophore transporter (Afu3g03640); SRGC6 encodes the orthologue of the *A. nidulans* iron regulator HapX (Hortschansky *et al*., 2007); SRGC7 encodes a putative siderophore transporter (Afu7g06060); and SRGC8 encodes the orthologue (Afu8g02760) of the *A. nidulans* mitochondrial ornithine carrier AmcA, which is likely to be involved in supply of ornithine for siderophore biosynthesis (Oberegger *et al*., 2001). Moreover, the SRGCs encode putative novel components of the siderophore system (Fig. 6, Table 2), e.g. ABC transporters that are possibly involved in excretion of siderophores (Afu3g03430 and Afu3g03670) and a putative siderophore-degrading enzyme (Afu3g03390). In this respect it is noteworthy that *A. fumigatus* exhibits EstB-independent TAFC hydrolysis (Kragl *et al*., 2007). CgrA from SRGC8 functions in ribosome biosynthesis and is required for thermotolerance and virulence (Bhabhra *et al*., 2004). CgrA deficiency had no impact on production of TAFC and FC (data not shown), ruling out a direct role in siderophore biosynthesis. However, the *cgrA*-deletion mutant displayed reduced conidiation during iron-depleted conditions at 20°C, a temperature at which CgrA deficiency causes no phenotype in standard growth media (data not shown), which indicates that CgrA is required in particular during iron-depleted conditions. SRGC5 encodes FetC and FtrA, which are key components of the reductive iron assimilatory system.

**Table 2:** Comparison of the genomic organization of *A. fumigatus* SreA-regulated genes with that of homologues from *A. nidulans* and *U. maydis*.

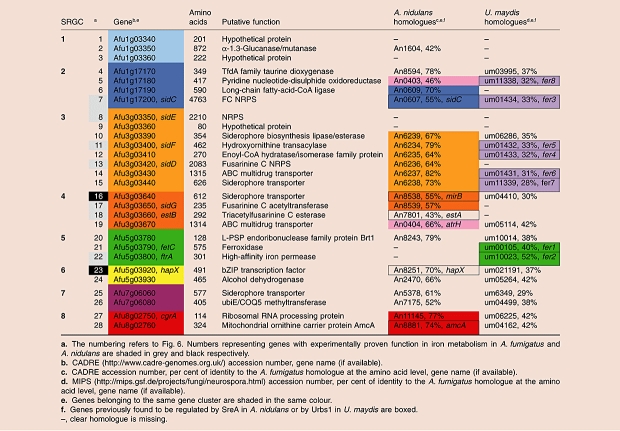

The only gene cluster not obviously linked to iron metabolism is SRGC1. The encoded Afu1g03350 displays high similarity to a *Trichoderma asperellum* α-1,3-glucanase that has cell wall-degrading and antifungal activity (Sanz *et al*., 2005), which implicates this protein in antagonistic functions, possibly to increase iron supply.

The 21 SreA-repressed genes that are not organized in gene clusters encode one known (SidA) and at least three further potential components of the iron homeostasis-maintaining system (Fig. 5): two putative siderophore transporters (Afu2g05730, Afu7g04730), and one putative ferric reductase (Afu1g17270) that might be involved in reductive iron assimilation (Martins *et al*., 1998; Kosman, 2003). SreA-mediated iron repression of the aspartic endopeptidase Pep1 (Afu5g13300) and a putative peptide transporter (Afu1g12240) suggests induction of protein degradation by iron starvation, possibly to facilitate iron acquisition. Interestingly, Pep1 was detected in sera of aspergillosis patients suggesting that it is produced by *A. fumigatus* during pathogenic growth (Reichard *et al*., 1994; 1995). Similarly, production of two secreted aspartyl proteinases, Sap10 and Sap99, is induced by iron starvation in *C. albicans*, correlating the expression of virulence traits with decreasing iron availability (Lan *et al*., 2004). SreA control of glycogen phosphorylase (Afu1g12920), alcohol dehydrogenase (Afu3g01490), isoamyl alcohol oxidase (Afu3g07410) and NAD-dependent formate dehydrogenase (Afu6g04920) links iron and carbon metabolism.

Selected genes displayed the same mode of regulation in Northern blot analysis (Fig. 4) and microarray-based expression profiling (Fig. 5). Moreover, SreA-mediated repression by iron of 15 genes identified in the microarray-based expression analysis during a shift from iron-depleted to iron-replete conditions was also confirmed by Northern analysis using RNA isolated from mycelia grown under steady-state iron-replete and -depleted conditions (Fig. S3). The congruence of the different approaches proves the validity of the employed methods.

The SreA regulon defined here contains all genes previously found to be involved in siderophore metabolism (*sidA*, *sidC*, *sidD*, *sidF*, *sidG*, *estB*) and reductive iron assimilation (*ftrA*, *fetC*) of *A. fumigatus*. Moreover, it contains orthologues to all known members of the *A. nidulans* SreA regulon (*sidA*, *sidC*, *amcA*, *mirB*, *estB* and *hapX*). As in *A. fumigatus*, both reductive iron assimilation and siderophore-mediated iron uptake are repressed by the respective SreA orthologues in *C. albicans*, *S. pombe* and *U. maydis* (Pelletier *et al*., 2003; Lan *et al*., 2004; Eichhorn *et al*., 2006). In contrast, the SreA orthologue Cir1 serves as a repressor for reductive iron assimilation but as an activator for siderophore-mediated iron uptake in *C. neoformans* (Jung *et al*., 2006), indicating fundamental differences in the mode of iron regulation in this basidiomycete species. The identification of putative novel components will guide the further characterization of the iron homeostasis-maintaining system of *A. fumigatus*.

For most SreA targets, SreA deficiency results only in partial derepression during iron-replete conditions (Fig. 5), which indicates additional SreA-independent iron regulatory mechanisms as previously suggested for *A. nidulans* (Haas *et al*., 1999; Oberegger *et al*., 2002b; Hortschansky *et al*., 2007). Interestingly, there are also several iron-repressed genes, which are largely unaffected by SreA deficiency, e.g. the ribotoxin Aspf1 [Afu5g02330 from cluster 4 (Table S1, Fig. S4A)], which was shown to be one of the major allergens of *A. fumigatus* (Lacadena *et al*., 2007).

### Promoter analysis of the SreA regulon genes

By mutational promoter analysis and DNA–protein interaction studies, genes involved in siderophore metabolism and reductive iron assimilation have been found to be directly regulated by SreA orthologues in *U. maydis* and *S. pombe*, respectively, via binding to the canonical consensus HGATAR motif (An *et al*., 1997; Pelletier *et al*., 2002; 2003). Promoter analysis as described in *Experimental procedures* identified several HGATAR motifs in promoters of all 49 putative SreA-regulated genes (Table S2 and Fig. 5). However, the occurrence of this motif was just slightly higher in the upstream sequences of the 49 genes compared with those in the genome (Tables S2 and S3). An extended and partial palindromic variation of this motif, ATCWGATAA, was discovered to be significantly over-represented in the most upstream regions of the 49 putative SreA-regulated genes, with an average copy number being 5.4-fold higher than that in the whole genome (Table S3). Strikingly, the identified *in vivo* binding sites for SreA orthologues of *U. maydis sid1* (siderophore biosynthesis), *S. pombe str1* (siderophore transporter) and *S. pombe fio1-fip1* (reductive iron assimilatory gene cluster) comply with the ATCWGATAA motif (An *et al*., 1997; Pelletier *et al*., 2002; 2003). Similar to this motif, palindromic GATA motifs bearing complete or partial GATA sequence also have been reported to be present in the genes of various organisms, including those coding for the GATA-1 factor in vertebrates (Trainor *et al*., 1996). These data suggest that direct control by SreA of the SreA regulon members is likely.

### Comparison of the genomic organization of the *A. fumigatus* SreA regulon with that of *A. nidulans* and *U. maydis*

*Aspergillus nidulans* employs the same siderophores and the same mode of iron regulation as *A. fumigatus*. Analysis of the genomic organization (Fig. 6, Table 2) revealed that genomic clustering of most genes of SRGC2, SRGC3, SRGC4 and SRGC8 has been evolutionary conserved in these two species. However, several genes have changed the order and/or orientation within the clusters (data not shown). Remarkably, An0403 (the orthologue of *A. fumigatus* Afu1g17180 from SRGC2) and An0404 (the orthologue of *A. fumigatus* Afu3g03670 from SRGC3) build a cluster on their own in *A. nidulans* (Table 2). In neither *A. fumigatus* nor *A. nidulans* is *sidA* clustered with other iron-regulated genes. In contrast, *sidA* orthologues are clustered with a siderophore NRPS in *S. pombe* and *U. maydis* (Yuan *et al*., 2001; Schrettl *et al*., 2004b)*. U. maydis* produces two siderophores, ferrichrome and ferrichrome A, the expression of which is regulated by the SreA orthologue Urbs1 (Leong and Winkelmann, 1998). Recently, Urbs1-controlled genes have also been found to be organized in gene clusters (Eichhorn *et al*., 2006), which comprise orthologues of genes from *A. fumigatus* SRGC2, SRGC3 and SRGC5 (Table 2). This comparison indicates that all siderophore biosynthetic genes might originate from a single-gene cluster and have subsequently reorganized and separated during genome evolution and speciation.

For some of the *A. fumigatus* genes, no clear homologue could be detected in *A. nidulans* or *U. maydis* (Table 2), for example, the NRPS SidE (Afu3g03350) that has previously been suggested to be involved in siderophore biosynthesis (Reiber *et al*., 2005; Perrin *et al*., 2007). Because of its absence in *A. nidulans*, however, a direct role of SidE in biosynthesis of TAFC or FC seems unlikely. In agreement, *sidE* but not *sidD* or *sidC* is regulated by LaeA, a regulator of many secondary metabolite gene clusters (Perrin *et al*., 2007). Vice versa, SreA does not affect expression of LaeA-controlled secondary metabolism gene clusters, e.g. the gliotoxin gene cluster (data not shown), indicating that SreA and LaeA can act independently and do not control expression of gene clusters in general. The absence of orthologues of SidG and EstB in *U. maydis* can be explained by the fact that *U. maydis* does not produce fusarinine-type siderophores. Consistent with *A. nidulans* lacking reductive iron assimilation (Eisendle *et al*., 2003), orthologues of the genes from *A. fumigatus* SGRC5 are missing in *A. nidulans*. In contrast, the genes encoding ferroxidase and iron permease are present and clustered in *U. maydis*, *S. pombe* and *C. neoformans* (Pelletier *et al*., 2002; Lian *et al*., 2005; Eichhorn *et al*., 2006).

### Genes upregulated by SreA deficiency

Transcript levels of genes from clusters 14–18 increased within 10 min after addition of iron (Table S1, Fig. S4B). This fast response might indicate a post-translational, rather than a transcriptional, activation of the respective regulatory mechanism. In this respect it is interesting to note that iron-related pathways (including haem-related enzymes and iron-sulphur-cluster-containing enzymes) are repressed during iron-depleted conditions via interaction of HapX with the CCAAT-binding (Hap) complex in *A. nidulans* (Hortschansky *et al*., 2007). Iron destroys this interaction, thereby derepressing transcription of the target genes. In *A. nidulans*, HapX and SreA display mutual transcriptional control. *A. fumigatus* possesses a HapX orthologue (Afu5g03920), the expression of which is controlled by SreA (Fig. 5), suggesting conservation of this iron-regulatory network in *A. fumigatus.*

**Fig. 7:**
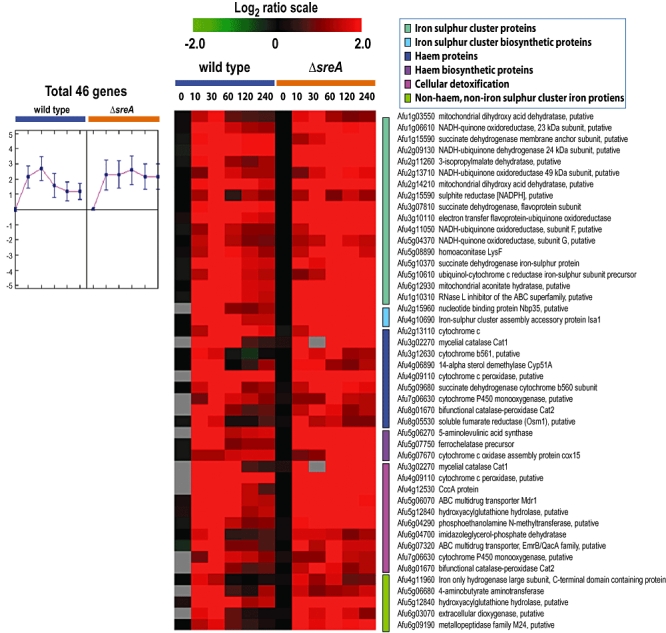
Heatmap representation of expression profiles of genes that are upregulated in the *A. fumigatus* Δ*sreA* strain and that are related to iron or cellular detoxification. Genes were selected for display from clusters 14 and 15 in Fig. S3B for their predicted functions related to iron or cellular detoxification. Microarray data from the iron concentration shift assay with the wild-type and Δ*sreA* strains are displayed in parallel for comparison. The bar at the top indicates colours corresponding to the range of the observed expression ratios on a log_2_ scale. Genes are displayed in the order of chromosomal localization. Colour-coded boxes denote the predicted functional categories to which the genes belong. A graph left to the heatmap displays average ratio values with error bars of the data from each time point.

The expression of 80 genes, clusters 14 and 15, increased for about 30 min and then returned to a lower level in the wild type, while the transcript levels of the same genes persisted at an elevated level in the Δ*sreA* strain (Table S1, Fig. S4B). The similar mode of induction of these genes in wild-type and Δ*sreA* strains suggests that SreA does not directly regulate these genes. Their upregulation is rather caused indirectly by metabolic changes due to SreA deficiency, e.g. iron overload and oxidative stress. In agreement, 36 of these 80 genes encode iron-related proteins, i.e. haem proteins, haem biosynthetic proteins, iron-sulphur-cluster proteins, iron-sulphur-cluster biosynthetic proteins and non-haem iron proteins (Fig. 7). Consistently, the SreA-deficient mutant displayed significantly increased contents in haem and the haem precursor coproporphyrin and a slightly increased content in the haem precursor protoporphyrin IX (Table 3). The increased production of iron-containing proteins in the *sreA* disruption mutant could be a direct result of inactivation of HapX/CCAAT-binding complex interaction by iron excess and could serve for detoxification of cellular iron. Moreover, 10 of the 80 gene products are potentially involved in general detoxification processes (Table S1, Fig. S3B, Fig. 7): for example, the catalase Cat1, the catalase/peroxidase Cat2, cytochrome *c* peroxidase, hydroxyacylglutathione hydrolase (glyoxylase II) and two ABC multidrug transporters. Upregulation of the encoding genes in the *sreA*-disruption mutant indicates increased oxidative stress, which is in agreement with the increased sensitivity to menadion (Fig. 3). In agreement, the genes encoding Cat1, Cat2 and cytochrome *c* peroxidase are upregulated by oxidative stress via the transcription factor AfYap1 (Lessing *et al*., 2007). The activity of these three antioxidative enzymes depends on iron as cofactor, which explains their downregulation during iron starvation and underscores the cross-linking of iron and oxidative metabolism. Consistently, iron starvation renders *A. fumigatus* hypersensitive to oxidative stress (Schrettl *et al*., 2007). Upregulation of the ABC multidrug transporter Mdr1 indicates that SreA deficiency results in accumulation of toxic compounds as Mdr1 and its *A. nidulans* orthologue AtrD confer resistance to various cytotoxic compounds (Tobin *et al*., 1997; Andrade *et al*., 2000). Similar to *A. fumigatus*, SreA deficiency causes upregulation of genes encoding aconitase, CatB (orthologue of *A. fumigatus* Cat1) and cytochrome *c* during iron-replete conditions in *A. nidulans* (Oberegger *et al*., 2001; 2002b), demonstrating a similar response to iron excess in *A. fumigatus* and *A. nidulans*.

**Table 3:** Contents in haem and haem precursers of *A. fumigatus* wild type and Δ*sreA*.

Precursors/haem	Wild type	Δ*sreA*	Δ*sreA/*wild type
Coproporphyrin	18.1 ± 2.3	142.1 ± 31.0	7.6
Protoporphyrin IX	48.6 ± 8.1	62.6 ± 11.6	1.3
Haem	284.9 ± 6.8	615.5 ± 19.9	2.2

Strains were grown in liquid culture for 24 h under iron-replete conditions. The data represent the means ± standard deviations of results from three independent measurements. Values are given in pmol mg^−1^protein.

### The *ΔsreA* mutant has wild-type virulence

**Fig. 8:**
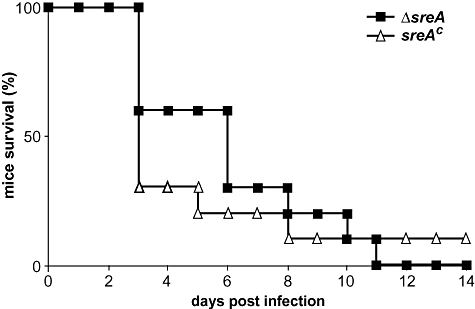
Virulence analysis of *A. fumigatus* Δ*sreA* (closed squares) and *sreA*^*c*^ (open triangles) strains. *sreA*^*c*^ displayed wild-type virulence (data not shown).

To determine whether SreA-mediated regulation is relevant to the growth of *A. fumigatus* in the environment of the host, we compared the virulence of the Δ*sreA* mutant with the complemented strain *sreA*^*c*^ in an immunosuppressed mouse model of invasive aspergillosis (Liebmann *et al*., 2004; Kupfahl *et al*., 2006). The mice were immunosuppressed with a single dose of cortisone acetate on day −1 and with cyclophosphamide every third day starting 4 days prior to infection. Survival was monitored for 14 days following intranasal inoculation with 4 × 10^4^ conidia on day 0. The Δ*sreA* mutant showed no difference regarding virulence (Fig. 8).

As indicated by Northern blot analysis (see above), SreA is expressed under iron-replete conditions only and, consistently, deleterious effects of *sreA*-disruption are limited to iron-replete conditions (see above). Therefore, the wild-type virulence of the Δ*sreA* mutant is in agreement with *A. fumigatus* facing iron-limited conditions in the host, which is also in accordance with the importance of siderophores for pathogenicity (Schrettl *et al*., 2004a; 2007). In striking contrast, the *C. neoformans* SreAorthologue Cir1 is essential for pathogenicity (Jung *et al*., 2006). However, Cir1 functions, directly or indirectly, also as an activator for growth at 37°C (host temperature) and capsule formation, which are both important virulence traits. In contrast, SreA does not appear to positively influence virulence determinants and its deficiency does not affect temperature resistance.

### Conclusions

Sensing of the environment is crucial for the survival of *A. fumigatus* during both saprophytic and pathogenic growth. Here we identified and characterized the transcriptional regulator SreA that is required for adaptation to the ambient iron availability. SreA was found to repress iron uptake during iron-replete conditions and consequently its genetic inactivation resulted in over-accumulation of iron, partially buffered by upregulation of intracellular iron chelation by siderophores. Microarray- and Northern-based expression profiling demonstrated control by SreA of all known genes involved in the two high-affinity iron uptake systems of *A. fumigatus*, siderophore-mediated iron uptake and reductive iron assimilation. Consistent with an iron- and oxidative stress-sensitive phenotype, the Δ*sreA* mutant displayed upregulation of iron-dependent and oxidative stress detoxifying pathways during iron-replete conditions, which appears to be caused indirectly by the excess iron accumulation. The identification of putative novel components of the *A. fumigatus* iron homeostasis-maintaining system by the undertaken transcriptomics approach will aid in the further characterization of this virulence-determining pathway. SreA regulation of the α-1,3-glucanase Afu1g03350, a potentially cell wall-degrading and antifungal protein (Sanz *et al*., 2005), and the endopeptidase Pep1 implicates these antagonistic enzymes in indirect improvement of iron supply. Alternatively, this control could be an example for iron availability serving as a regulatory signal for expression of virulence-related functions as suggested for other prokaryotic and eukaryotic pathogens (Weinberg, 1999). SreA appears to act only as a repressor and seems not to be required for expression of any virulence trait. In contrast, the *C. neoformans* SreA orthologue Cir1 functions as both repressor and activator, and it is essential for expression of several virulence determinants (Jung *et al*., 2006). This distinction most likely explains why SreA is dispensable for virulence in a mouse model whereas Cir1 is essential. Notably, there is a significant structural difference between Cir1 and all of its fungal orthologues, which might account for or reflect the different features: Cir1 contains only a single zinc finger instead of a double zinc finger.

## Experimental procedures

### Fungal strains, growth conditions, analysis of siderophore production and iron content

Fungal strains used were *A. fumigatus* wild-type ATCC46645 (American Type Culture Collection), Δ*sreA* (*ATCC46645*, Δ*sreA::hph*) and *sreA*^*c*^ (Δ*sreA, (p)::sreA*). Δ*sreA* and *sreA*^*c*^ were generated during this study as described below. Generally, *A. fumigatus* strains were grown at 37°C in *Aspergillus* minimal medium according to Pontecorvo *et al*. (1953) containing 1% glucose as the carbon source and 20 mM glutamine as the nitrogen source. For iron-depleted conditions iron was omitted (AMM^−Fe^). For iron-replete and high-iron conditions, FeSO_4_ or TAFC^+Fe^ was added as indicated. The blood agar was AMM^−Fe^ containing 5% sheep blood. For growth assays, 10^4^ conidia of the respective fungal strain was point inoculated on plates containing the indicated growth medium and incubated at 37°C.

Reversed-phase HPLC analysis of siderophore production and short-term uptake of ^55^Fe-labelled TAFC^+Fe^ were carried out as described previously (Oberegger *et al*., 2001).

For determination of the total cellular iron content, 50 mg of freeze-dried mycelia was decomposed in closed PTFE vessels containing 2 ml of HNO_3_ and 0.5 ml of hydrogen peroxide using a high-performance microwave digestion unit (mls-1200 mega). Appropriate dilutions were made with distilled water and the total iron content was determined by graphite furnace atomic absorption spectrometry (Hitachi Polarized Zeeman AAS Z8200) following standard methods.

### cDNA sequence, Northern analysis and DNA manipulations

RNA was isolated using TRI Reagent™ (Sigma). The *A. fumigatus sreA* cDNA sequence was analysed by reverse transcribed-PCR using Superscript™ (Invitrogen). The 5′ and 3′ ends were determined with the GenRacer™ method (Invitrogen) using total RNA.

For Northern analysis, 5 μg of total RNA was electrophoresed on 1.2% agarose-2.2 M formaldehyde gels and blotted onto Hybond N membranes (Amersham). The hybridization probes used in this study were generated by PCR. Hybridization probes and Primers used are listed in Table S4. For extraction of genomic DNA, mycelia were homogenized and DNA was isolated according to Sambrook *et al*. (1989). For general DNA propagations *Escherichia coli* DH5α strain was used as a host.

### Disruption of *sreA* and complementation of the *ΔsreA* strain

To construct the Δ*sreA* allele, a 5.6 kb fragment was amplified using primers oAfsreA1 and oAfsreA2, subcloned into the pGEM-T, yielding plasmid pSreA. Subsequently, an internal 0.4 kb BssHII–EcoRV fragment was replaced by the 2.4 kb BssHII–EcoRV fragment of vector pHPH. pHPH was generated by insertion of the 2.3 kb AvrII–XbaI *hph* marker-encoding fragment of pAN7-1 (Punt *et al*., 1987) into the SpeI site of pBluescript-KS (Stratagene). For transformation of *A. fumigatus* ATCC46645, the gel-purified 7.4 kb DraI fragment of pSreA was used.

Taking advantage of the decreased resistance of the Δ*sreA* mutant to phleomycin (Fig. 3), Δ*sreA* protoplasts were transformed with pSreA and screened for wild-type resistance to phleomycin for genetic complementation. Several strains with one to several ectopic integrations of pSreA were identified displaying wild type-like siderophore production and growth characteristics. Data of one strain, termed *sreA*^*c*^, are presented here.

Transformation of *A. fumigatus* was carried out as previously described for *A. nidulans* (Tilburn *et al*., 1995). Selection of transformants was carried out on plates containing 200 μg ml^−1^ hygromycin B (Calbiochem) or 5 μg ml^−1^ phleomycin (Sigma) respectively. Subsequent to a 24 h incubation period at 20°C, the plates were overlaid with 5 ml of soft agar containing the same concentration of 200 μg ml^−1^ hygromycin B or 10 μg ml^−1^ phleomycin, respectively, and incubated for 3 days at 37°C. Screening of transformants was performed by PCR; ectopic and single homologous genomic integrations were confirmed by Southern blot analysis.

### Transcriptional profiling

The *A. fumigatus* Af293 DNA amplicon microarray containing 9516 genes (Nierman *et al*., 2005) was used in this study. To profile the genome-wide expression responses of the fungus to the shift from iron-depleted to iron-replete conditions and to identify the genes regulated by SreA, we conducted microarray analysis with the wild-type and Δ*sreA* strains using the same RNA samples that were used for the Northern blot analysis (see above). Labelling reactions with RNA and hybridization were conducted as described in the PFGRC standard operating procedures (PFGRC SOPs) found at (http://pfgrc.jcvi.org/index.php/microarray/protocols.html). The sample from 0 h served as reference in all hybridizations with samples from later time points within each strain (i.e. wild type and Δ*sreA* strain) in order to identify genes exhibiting altered transcription after the shift from iron-depleted to iron-replete conditions. All the hybridizations were repeated in dye-swap sets. Hybridized slides were scanned using the Axon GenePix 4000B microarray scanner and the TIFF images generated were analysed using TIGR Spotfinder (http://www.jcvi.org/cms/research/software/, TIGR) to obtain relative transcript levels. Data from TIGR Spotfinder were stored in MAD, a relational database designed to effectively capture and store microarray data. Data were normalized using a local regression technique LOWESS (LOcally WEighted Scatterplot Smoothing) for hybridizations using a software tool MIDAS (http://www.jcvi.org/cms/research/software, TIGR). The resulting data were averaged from triplicate genes on each array and from duplicate flip-dye arrays for each experiment, taking a total of six intensity data points for each gene. Differentially expressed genes at the 95% confidence level were determined using intensity-dependent *Z*-scores (with *Z* = 1.96) as implemented in MIDAS and the union of all genes identified at each time point from the wild type were considered significant in this experiment. The resulting data were organized and visualized using *k*-means algorithm to find the genes that are differentially regulated between the wild type and the Δ*sreA* strain. Selected genes that appeared to be regulated by SreA were separately organized and visualized based on chromosomal locations with TIGR MEV (http://www.tm4.org, JCVI).

### Promoter motif studies for the putative SreA-regulated genes

To study the over-represented motifs in the 49 putative SreA-regulated genes found by microarray analyses (Fig. 5), upstream sequences spanning from −2000 to −1 bp relative to the start codon of the genes were retrieved. Among these, the sequences from those 22 genes, association of which with SreA is further supported by their proven or likely direct function in iron uptake (Table S5), were subjected to *de novo* motif searches by oligo- and dyad-analysis tools available from the Regulatory Sequence Analysis Tools (RSAT) package (http://rsat.ulb.ac.be/rsat/). The motifs found from the analyses were mapped to the corresponding upstream sequences, and those with the Occurrence Significance index higher than 8 were retrieved. A new motif with the consensus sequence of ATCWGATAA was discovered from these analyses. A collection of sequences that belong to this group of motif was then used to generate an alignment matrix with SEQLOGO (http://www.bioinf.ebc.ee/EP/EP/SEQLOGO/) for further analysis. The pattern matching analysis with the previously identified motif of HGATAR and the newly discovered ATCWGATAA on the upstream sequences from all 49 genes was conducted using patser analysis tool from RSAT. The search results were displayed in graphics using the ‘feature map’ tool that also is available in RSAT. The upstream sequences from the whole genome of *A. fumigatus* also were retrieved and subjected to the same motif analysis to be compared with the results with the 49 putative SreA-regulated genes. Default values were used for the most parameters in patser except that the lower threshold estimation of weight score was set at 5.0 and 8.0 for HGATAR and ATCWGATAA, respectively, and Alphabet was set at 0.25 for both A:T and G:C. The alignment matrices used for the analyses are provided in Fig. S5.

### Virulence assay

Virulence assays in a mouse model for pulmonary aspergillosis were performed as described previously (Liebmann *et al*., 2004; Kupfahl *et al*., 2006). Survival curves were compared using Kaplan–Meier log rank analysis.

### Accession numbers

Described genes and proteins can be retrieved with the respective ‘Afu’ accession numbers from the CoreNucleotide database at NCBI (http://www.ncbi.nlm.nih.gov/sites/entrez) or from the Central Aspergillus Data Repository (CADRE: http://www.cadre-genomes.org.uk/).
