# Supplementary material for: SreA-mediated iron regulation in Aspergillus fumigatus
Source: Mol Microbiol. 2008 Aug 21;70(1):27–43. doi: 10.1111/j.1365-2958.2008.06376.x (PMC2610380; doi:10.1111/j.1365-2958.2008.06376.x)
Supplement: Supplementary file 1 [file mmi0070-0027-SD1.pdf]

Table S1. Expression ratio values and associated Gene Ontology (GO) categories for the 1,147 genes displayed in Fig. S4ABC.
Expression ratio values are shown for the wild-type and Δ*sreA* strain. Genes with GO categories are listed in the order they appear in clusters 1-25 in Fig. S4ABC.

Cluster	Gene	Description	wild-type						Δ*sreA*							
			0	10	30	60	120	240	0	10	30	60	120	240	slim_GO_1	
1	Afu5g03800	high-affinity iron permease CaFTR2	0,006	0,448	-4,172	-4,172	-3,547	-1,671	-0,076	0,092	-0,841	-0,604	-0,519	-0,282	GO:0006810	transport
1	Afu7g04730	siderochrome-iron transporter, putative	-0,024	-0,452	-4,699	-4,659	-4,519	-3,066	-0,002	-0,500	-0,653	-0,776	-0,563	-0,895	GO:0006810	transport
1	Afu2g07680	L-ornithine N5-oxygenase SidA	0,023	-0,138	-4,174	-4,236	-4,176	-3,512	-0,040	-0,118	-1,007	-0,923	-0,839	-0,640	GO:0019748	secondary metabolism
1	Afu7g06060	siderochrome-iron transporter (Sit1), putative	-0,012	0,249	-3,894	-4,211	-4,142	-2,634	0,018	-0,527	-1,022	-1,048	-0,631	-1,037	GO:0006810	transport
1	Afu3g03640	siderochrome-iron transporter (MirB), putative	0,005	-0,513	-3,598	-4,830	-4,868	-4,686	0,011	-0,509	-1,246	-0,467	-1,224	-0,894	GO:0019725	homeostasis
1	Afu3g03670	ABC multidrug transporter, putative	-0,029	-0,975	-2,959	-3,802	-4,175	-3,958	-0,026	-0,694	-1,416	-1,357	-1,659	-1,570	GO:0006810	transport
1	Afu3g03420	hypothetical protein	-0,039	-0,205	-2,547	-3,310	-3,547	-3,199	-0,043	-0,391	-0,601	-0,870	-1,129	-0,915	GO:0019748	secondary metabolism
1	Afu3g03440	MFS family siderophore transporter, putative	0,026	-1,041	-3,908	-3,740	-4,144	-3,578	0,000	-0,843	-0,739	-0,520	-0,907	-1,254	GO:0006810	transport
1	Afu3g03430	ABC multidrug transporter SitT, putative	-0,011	-0,518	-3,660	-3,915	-4,158	-3,366	0,003	-0,656	-0,722	-0,821	-1,080	-1,285	GO:0006810	transport
1	Afu3g03410	enoyl-CoA hydratase/isomerase family protein	-0,011	-0,518	-3,156	-3,857	-4,088	-3,434	-0,014	-0,157	-0,910	-0,984	-1,012	-1,154	GO:0019748	secondary metabolism
1	Afu3g03400	siderophore biosynthesis acetylase AceI, putative	0,005	-0,964	-3,871	-3,495	-3,513	-3,246	-0,018	-0,584	-1,483	-1,021	-1,255	-1,238	GO:0019748	secondary metabolism
1	Afu1g17180	pyridine nucleotide-disulphide oxidoreductase, putative	0,021	-0,663	-3,665	-3,190	-3,217	-3,036	0,013	-0,759	-1,627	-1,231	-1,543	-1,533	GO:0006259	DNA metabolism
1	Afu1g17190	long-chain-fatty-acid-CoA ligase, putative	0,025	-0,478	-3,625	-3,156	-3,370	-3,133	-0,014	-0,517	-1,118	-0,784	-1,088	-1,220	GO:0006810	transport
2	Afu1g12920	glycogen phosphorylase 1; possible glycogen phosphorylase	0,002	0,213	-1,038	-2,111	-2,491	-2,277	-0,056	0,185	-0,626	-1,331	-1,648	-1,777	GO:0006091	energy pathways
2	Afu1g03360	hypothetical protein	-0,021	-1,337	-1,530	-1,587	-2,372	-2,942	0,047	-1,111	-0,461	-0,445	-0,676	-1,164	GO:0000004	unknown
2	Afu1g03340	hypothetical protein	-0,034	-0,387	-2,111	-1,593	-2,012	-2,500	-0,002	-0,486	-0,466	-0,169	-0,405	-0,900	GO:0000004	unknown
2	Afu1g03350	alpha-1,3-glucanase, putative	-0,007	-0,184	-1,977	-1,805	-2,674	-3,213	-0,018	-0,572	-0,588	-0,060	-0,912	-1,337	GO:0005975	carbohydrate metabolism
2	Afu1g13990	conserved hypothetical protein	-0,013	-1,104	-1,404	-1,446	-2,588	-2,750	-0,077	-0,649	-1,185	-1,347	-1,585	-1,769	GO:0000004	unknown
2	Afu4g14070	glycosyl transferase, putative	-0,036	-0,304	-1,409	-1,497	-1,848	-1,897	-0,202	-0,337	-1,102	-1,132	-0,845	-1,010	GO:0005975	carbohydrate metabolism
2	Afu6g13590	3-isopropylmalate dehydrogenase	0,024	-0,634	-1,464	-1,803	-1,662	-1,435	0,020	-0,207	-0,678	-1,175	-0,991	-0,855	GO:0006519	amino acid
2	Afu3g03650	acetyltransferase, GNAT family, putative	0,012	-0,736	-2,067	-1,826	-2,065	-1,828	-0,018	-0,362	-1,070	-1,009	-0,850	-0,830	GO:0000004	unknown
2	Afu1g17200	nonribosomal peptide synthase, putative	-0,016	-0,661	-2,465	-2,366	-2,545	-2,149	-0,086	-0,956	-1,223	-1,365	-1,305	-0,939	GO:0019748	secondary metabolism
3	Afu6g04920	NAD-dependent formate dehydrogenase	0,027	-1,051	-2,169	-2,501	-2,312	-1,597	-0,048	-0,732	-1,206	-1,762	-0,815	1,013	GO:0006732	coenzyme metabolism
3	Afu8g02760	mitochondrial ornithine carrier protein (AmcA), putative	0,020	0,385	-2,346	-2,544	-2,648	-2,430	0,019	0,153	-0,503	-1,133	-1,156	-1,211	GO:0006519	amino acid
3	Afu1g17170	alpha-ketoglutarate-dependent taurine dioxygenase	-0,019	-0,537	-2,926	-2,269	-1,305	-0,922	0,063	-0,787	-1,435	-1,056	-0,576	-0,718	GO:0000004	unknown
3	Afu3g03390	siderophore biosynthesis lipase/esterase, putative	0,037	-0,943	-2,869	-2,154	-2,248	-1,638	-0,025	-0,379	-0,614	-0,906	-0,917	-0,934	GO:0019748	secondary metabolism
3	Afu5g03920	bZIP transcription factor (HapX), putative	-0,009	-0,623	-3,096	-2,557	-2,357	-1,898	-0,031	-0,386	-0,246	-0,098	-0,252	-0,375	GO:0045449	regulation of transcription
3	Afu1g17270	ferric-chelate reductase (Fre2), putative	0,067	-0,759	-2,889	-2,459	-2,645	-2,199	-0,010	-0,468	-0,361	0,173	-0,372	-0,360	GO:0006810	transport
3	Afu2g05730	siderochrome-iron transporter (MirC), putative	0,003	0,222	-2,175	-1,663	-1,547	-0,943	-0,036	-0,283	-0,090	-0,276	-0,387	-0,239	GO:0019725	homeostasis
3	Afu4g10100	FAD binding domain protein	-0,035	0,751	-2,751	-2,130	-1,657	-0,474	-0,045	-0,055	-0,791	-0,497	-0,416	-0,397	GO:0000004	unknown
3	Afu5g03780	L-PSP endoribonuclease family protein (Brt1), putative	-0,024	0,422	-2,536	-2,341	-2,306	-1,349	0,016	0,219	-0,723	-0,750	-0,406	-0,520	GO:0000004	unknown
3	Afu3g13500	hypothetical protein	-0,004	-1,044	-2,358	-1,098	-1,229	-1,152	0,010	-0,039	-0,130	-0,333	-0,309	-0,265	GO:0000004	unknown
3	Afu7g02330	hypothetical protein	-0,012	-0,908	-2,329	-1,119	-1,111	-1,070	0,034	-0,005	-0,197	-0,367	-0,244	-0,104	GO:0000004	unknown
3	Afu5g02700	multidrug resistant protein	-0,052	-0,702	-1,926	-1,440	-2,091	-1,416	-0,017	-1,000	-0,395	-0,358	-0,495	-0,591	GO:0006810	transport
3	Afu1g12690	ABC multidrug transporter Mdr4	0,107	-0,713	-2,164	-1,491	-1,534	-1,403	0,033	-0,380	-0,257	-0,245	-0,798	-0,892	GO:0006810	transport
3	Afu5g03930	alcohol dehydrogenase, putative	-0,076	-0,409	-2,048	-1,489	-1,535	-1,685	-0,006	-0,168	-0,490	-0,477	-0,731	-0,690	GO:0005975	carbohydrate metabolism
3	Afu6g03680	hypothetical protein	-0,025	-1,834	-2,027	-1,732	-1,702	-1,735	0,172	-0,284	-0,402	-0,518	-0,287	-0,307	GO:0000004	unknown
3	Afu3g07410	isoamyl alcohol oxidase	-0,084	-1,216	-1,612	-1,514	-1,596	-1,547	0,024	-0,377	-0,523	-0,672	-0,352	-0,550	GO:0000004	unknown
3	Afu1g12240	MFS peptide transporter, putaitve	-0,020	-0,964	-2,067	-1,884	-2,065	-1,941	-0,001	-0,351	-0,362	-0,189	-0,267	-0,221	GO:0006810	transport
3	Afu8g02750	nucleolar protein CgrA	-0,001	-0,121	-1,090	-1,570	-1,800	-1,951	0,017	0,293	0,147	-0,216	-0,563	-0,662	GO:0006996	organelle organization and biogenesis
3	Afu5g13300	aspartic endopeptidase Pep1	-0,003	-0,483	-1,168	-1,360	-1,460	-1,246	-0,002	0,114	-0,054	-0,148	-0,657	-0,386	GO:0030163	protein catabolism
3	Afu5g00710	GABA permease, putative	-0,013	-0,081	-1,313	-1,707	-1,828	-1,502	0,020	0,303	-0,487	-1,060	-0,797	-0,342	GO:0006810	transport
3	Afu7g06130	integral membrane protein	-0,191	-0,020	-1,268	-1,165	-1,187	-1,072	-0,068	-0,206	-0,563	-0,929	-0,508	-0,340	GO:0000004	unknown
3	Afu3g01490	alcohol dehydrogenase, putative	-0,004	-0,176	-1,647	-1,379	-1,676	-1,238	-0,024	-0,201	-0,613	-0,870	-0,517	-0,365	GO:0005975	carbohydrate metabolism
4	Afu7g06570	zinc/cadmium resistance protein	0,001	1,282	-1,284	-2,616	-2,792	-1,850	0,056	0,534	-0,217	-1,518	-1,598	-1,594	GO:0006810	transport
4	Afu5g02330	major allergen Asp F1	0,040	0,319	-0,685	-2,192	-2,602	-2,495	-0,010	0,619	-0,081	-1,825	-2,028	-1,919	GO:0000004	unknown
4	Afu4g01290	endo-chitosanase, pseudogene	0,039	0,376	-0,575	-2,724	-3,030	-2,611	0,010	0,771	-0,141	-2,418	-2,520	-1,473	GO:0005975	carbohydrate metabolism
4	Afu1g04300	hypothetical protein	0,039	0,399	-1,967	-1,105	-1,368	-1,182	0,006	0,152	-1,358	-2,085	-1,280	-1,499	GO:0000004	unknown
4	Afu5g09400	carbonyl reductase, putative	-0,006	-0,210	-0,940	-1,660	-1,337	-1,099	-0,020	-0,180	-0,767	-1,889	-2,428	-2,154	GO:0000004	unknown
4	Afu5g00720	acetyltransferase, GNAT family family	-0,016	0,064	-1,101	-2,384	-1,560	-1,096	-0,071	0,289	-0,157	-1,797	-1,476	-1,029	GO:0000004	unknown
4	Afu1g09930	glycerol dehydrogenase (GCY1), putative	0,013	-0,105	-0,744	-1,880	-1,809	-0,959	-0,050	-0,072	-0,610	-1,365	-1,562	-1,091	GO:0000004	unknown
4	Afu6g11430	aldehyde dehydrogenase, putative	-0,021	0,199	-0,082	-1,581	-1,924	-1,482	0,001	0,152	-0,573	-1,212	-1,444	-1,587	GO:0000004	unknown
4	Afu1g03150	c-14 sterol reductase	-0,047	2,163	-1,106	-1,740	-1,177	-0,452	-0,034	1,015	-1,139	-2,100	-1,422	-1,192	GO:0006629	lipid metabolism
5	Afu3g03350	nonribosomal peptide synthase, putative	-0,020	-0,579	-0,359	-1,656	-2,171	-1,984	-0,028	0,089	0,046	-0,481	-0,279	-0,114	GO:0019748	secondary metabolism
5	Afu4g13080	monosaccharide transporter	-0,041	1,244	-1,342	-0,685	-1,701	-1,692	-0,039	0,413	0,049	-0,031	-0,409	-0,637	GO:0006810	transport
5	Afu3g08610	DUF124 domain protein	-0,050	0,786	-0,342	-0,975	-1,527	-1,622	-0,015	0,409	-0,292	-0,494	-0,496	-0,521	GO:0000004	unknown
5	Afu4g09710	C6 sexual development transcription factor Pro1	0,003	0,296	-0,037	-1,145	-1,782	-2,125	-0,038	-0,274	0,058	-0,434	-0,621	-0,999	GO:0045449	regulation of transcription
5	Afu5g13450	triosephosphate isomerase	0,007	-0,433	-0,776	-1,828	-1,582	-0,769	-0,060	-0,255	-0,563	-1,259	-1,715	-0,865	GO:0006091	energy pathways
5	Afu1g08880	heavy metal ion transporter, putative	0,068	0,126	-0,656	-1,955	-1,671	-0,619	-0,010	0,317	-0,425	-0,935	-1,259	-0,334	GO:0006810	transport
5	Afu3g08470	glucose-6-phosphate 1-dehydrogenase	-0,036	0,464	-0,191	-1,760	-1,499	-0,730	-0,033	0,562	-0,593	-0,869	-1,499	-0,747	GO:0006091	energy pathways
5	Afu6g08050	6-phosphogluconate dehydrogenase, decarboxylating	0,000	-0,121	-0,287	-1,604	-1,293	-0,174	-0,024	0,158	-0,093	-0,927	-1,790	-0,310	GO:0005975	carbohydrate metabolism
5	Afu3g03660	esterase superfamily protein	-0,035	-0,306	-1,086	-1,597	-1,508	-1,153	0,026	0,015	-0,174	-0,347	-0,285	-0,020	GO:0006629	lipid metabolism
5	Afu4g07060	acyl-CoA thioester hydrolase, putative	0,020	0,079	-1,525	-1,273	-1,204	-0,512	0,013	-0,159	-0,342	-0,492	-0,579	-0,214	GO:0006629	lipid metabolism
5	Afu1g03200	MFS transporter, putative	0,033	0,312	-1,411	-1,024	-1,047	-0,777	0,165	-0,055	-0,398	-0,665	-0,568	-0,476	GO:0006810	transport
5	Afu5g03790	ferro-O2-oxidoreductase	0,031	0,202	-1,106	-1,435	-1,582	-0,865	0,031	0,162	-0,179	-0,466	-0,264	-0,296	GO:0006810	transport
5	Afu6g03190	hypothetical protein	-0,002	0,584	-1,307	-1,146	-1,228	-1,006	0,016	0,129	-0,165	-0,222	-0,267	-0,267	GO:0000004	unknown

5	Afu1g14560	alpha-mannosidase	-0,006	-0,716	-1,207	-1,145	-1,373	-1,259	0,018	-0,099	-0,441	-0,477	-0,876	-0,448	GO:0000004	unknown
5	Afu2g13580	conserved hypothetical protein	0,040	-0,230	-1,374	-1,373	-1,429	-1,286	-0,118	-0,294	-0,640	-0,327	-1,108	-1,011	GO:0000004	unknown
5	Afu2g13630	amino transferase	-0,084	-0,254	-1,201	-1,371	-1,446	-1,071	-0,147	-0,682	-0,042	0,343	-0,838	-0,821	GO:0006519	amino acid
5	Afu5g02480	glycogen synthase	0,105	0,585	-1,078	-1,010	-1,180	-0,759	-0,044	-0,067	-0,982	-1,321	-0,990	-0,899	GO:0006091	energy pathways
5	Afu3g12330	phosphatidyl synthase	-0,018	0,425	-0,652	-0,798	-1,189	-0,766	-0,012	-0,023	-0,401	-1,095	-0,936	-0,567	GO:0000004	unknown
5	Afu4g14040	Hsp70 family protein	0,066	-0,126	-0,828	-0,962	-1,439	-1,491	-0,032	0,016	-0,674	-0,955	-0,891	-0,980	GO:0006464	protein modification
5	Afu3g11790	galactose-proton symport, putative	-0,050	0,521	-0,516	-0,841	-1,470	-1,503	0,111	-0,162	-0,520	-1,068	-0,700	-1,032	GO:0006810	transport
5	Afu6g00690	conserved hypothetical protein	-0,068	0,525	-0,443	-0,722	-1,120	-1,057	0,037	0,617	-0,308	-1,534	-0,985	-1,003	GO:0000004	unknown
5	Afu3g12790	conserved glutamic acid-rich protein	0,019	0,195	-0,873	-1,090	-0,737	-1,018	0,019	0,505	-0,511	-1,042	-0,785	-0,776	GO:0000004	unknown
5	Afu3g02550	conserved hypothetical protein	0,006	0,417	-0,545	-1,088	-0,876	-0,699	-0,023	0,597	-0,149	-0,559	-0,426	-0,421	GO:0000004	unknown
5	Afu6g03730	prpd protein	-0,053	-0,023	-0,478	-1,317	-1,350	-0,908	-0,013	0,069	-0,230	-0,837	-0,940	-0,661	GO:0000004	unknown
5	Afu6g09140	oxidoreductase, short-chain dehydrogenase/reductase family	0,016	-0,382	-0,597	-1,209	-1,385	-1,037	-0,017	0,143	-0,152	-0,871	-1,404	-0,827	GO:0006629	lipid metabolism
5	Afu2g08010	conserved hypothetical protein	-0,009	0,099	-0,039	-0,850	-1,167	-1,004	-0,005	0,042	-0,191	-0,780	-1,074	-0,974	GO:0000004	unknown
5	Afu4g14010	oxidoreductase, short chain dehydrogenase/reductase family superfamily	-0,009	-0,002	-0,368	-1,069	-1,210	-1,277	0,044	0,098	-0,175	-0,496	-0,760	-0,887	GO:0000004	unknown
5	Afu6g14460	2-haloalkanoic acid dehalogenase	-0,071	-0,025	-0,418	-1,004	-1,235	-1,019	0,008	0,049	-0,292	0,048	-0,686	-0,663	GO:0000004	unknown
5	Afu6g06520	WW domain protein	0,039	-0,120	-0,919	-1,296	-1,294	-0,788	-0,059	0,035	-0,718	-0,687	-0,753	-0,441	GO:0000004	unknown
5	Afu3g08110	cell wall protein, putative	-0,014	-0,065	-0,480	-1,226	-0,944	-0,950	-0,016	0,168	-0,185	-0,895	-0,556	-0,248	GO:0045229	cell wall and envelope biogenesis
5	Afu2g16570	short chain dehydrogenase/reductase, putative	-0,051	-0,419	-0,630	-0,947	-1,272	-1,138	-0,019	-0,214	-0,152	-0,644	-0,481	-0,306	GO:0000004	unknown
5	Afu6g04270	fructose symporter	0,094	1,590	-0,996	-0,554	-1,117	-0,645	-0,027	0,430	-0,935	-1,237	-1,177	-1,159	GO:0006810	transport
6	Afu6g06470	heat shock protein HSP30, putative	0,020	-0,045	0,052	-1,551	-0,935	0,753	-0,046	-0,664	0,989	0,037	-0,298	0,400	GO:0006464	protein modification
6	Afu8g02290	conserved hypothetical protein	NaN	-0,089	-0,008	-0,152	-1,174	-0,229	0,167	-0,149	-0,140	-0,225	-0,080	-0,142	GO:0000004	unknown
6	Afu2g02090	methylated-DNA-protein-cysteine methyltransferase	NaN	-0,249	-0,345	-0,040	-1,187	-0,012	0,033	-0,007	0,112	-0,063	-0,180	0,033	GO:0000004	unknown
6	Afu1g14710	beta-glucosidase 1	-0,261	-0,041	-0,282	-0,682	-0,447	-0,371	0,023	0,094	-0,389	-0,458	-0,378	-0,268	GO:0005975	carbohydrate metabolism
6	Afu4g01390	C6 transcription factor, putative	0,042	0,008	0,122	-0,843	-0,613	-0,354	0,077	-0,122	-0,181	-0,327	-0,398	-0,216	GO:0045449	regulation of transcription
6	Afu4g08600	aldehyde dehydrogenase, putative	-0,041	0,196	0,104	-0,938	-0,564	-0,287	-0,029	-0,153	-0,335	-0,320	-0,285	-0,187	GO:0006091	energy pathways
6	Afu5g02940	choline transport protein Ctr, putative	-0,045	0,005	-0,343	-0,243	-0,734	-0,697	-0,037	0,054	-0,018	-0,286	-0,341	-0,379	GO:0006810	transport
6	Afu1g06150	L-serine dehydratase, putative	0,015	0,168	-0,489	-0,597	-0,787	-0,606	0,002	-0,036	-0,026	-0,041	-0,308	-0,353	GO:0006519	amino acid
6	Afu8g01810	conserved hypothetical protein	0,110	0,215	0,061	-0,609	-0,963	-0,607	-0,080	0,161	-0,308	-0,405	-0,333	-0,529	GO:0000004	unknown
6	Afu8g01090	thioredoxin, putative	0,032	-0,038	-0,166	-0,679	-0,570	-0,643	0,088	0,236	-0,140	-0,497	-0,443	-0,326	GO:0006259	DNA metabolism
6	Afu4g11320	hypothetical protein	-0,020	0,102	-0,246	-0,666	-0,783	-0,875	0,017	0,056	-0,148	-0,436	-0,471	-0,378	GO:0000004	unknown
6	Afu7g03880	sodium/calcium exchanger protein	-0,102	0,020	-0,180	-0,185	-0,626	-0,248	-0,009	-0,226	-0,132	0,089	-0,302	-0,317	GO:0006810	transport
6	Afu3g00660	conserved hypothetical protein	0,029	0,207	-0,204	-0,316	-0,821	-0,375	0,106	-0,241	-0,271	-0,158	0,005	-0,402	GO:0000004	unknown
6	Afu4g13070	alpha/beta hydrolase, putative	-0,066	0,031	0,047	-0,586	-0,700	-0,367	0,003	-0,322	0,175	0,000	-0,265	-0,329	GO:0000004	unknown
6	Afu3g14870	hypothetical protein	NaN	0,549	-0,095	-0,613	-0,703	-0,378	-0,016	-0,039	-0,075	0,251	-0,339	-0,599	GO:0000004	unknown
6	Afu6g02860	isocitrate lyase	-0,001	0,364	0,047	-0,545	-0,839	-0,472	0,033	0,039	-0,109	-0,048	-0,270	-0,074	GO:0006091	energy pathways
6	Afu5g11430	quinone oxidoreductase, putative	-0,073	0,366	0,432	-0,438	-0,744	-0,483	0,069	-0,087	0,095	0,110	-0,326	-0,229	GO:0005975	carbohydrate metabolism
6	Afu7g05010	glyoxalase family protein	NaN	0,288	0,429	-0,368	-0,938	-0,568	-0,006	0,223	0,362	0,041	-0,239	-0,347	GO:0000004	unknown
6	Afu4g08660	oligosaccharyltransferase subunit ribophorin II, putative	-0,074	-0,143	0,002	-0,340	-0,585	-0,506	0,032	-0,124	-0,464	-0,195	-0,633	-0,557	GO:0006464	protein modification
6	Afu1g15430	lipase/esterase, putative	0,009	0,008	0,102	-0,230	-0,549	-0,342	-0,028	-0,149	-0,068	-0,501	-0,686	-0,597	GO:0006629	lipid metabolism
6	Afu6g00630	MFS transporter, putative	-0,014	-0,496	0,193	-0,357	-0,522	-0,858	-0,048	0,065	-0,087	-0,335	-0,595	-0,808	GO:0006810	transport
6	Afu1g01940	hypothetical protein	-0,226	-0,200	-0,093	-0,109	-0,679	-0,723	0,009	-0,234	-0,267	-0,399	-0,284	-0,704	GO:0000004	unknown
6	Afu2g11180	developmental regulator FlbA	-0,045	-0,159	0,096	-0,487	-0,771	-0,726	0,000	-0,388	-0,064	-0,463	-0,418	-0,765	GO:0007165	signal transduction
6	Afu6g12880	hypothetical protein	-0,074	-0,507	0,097	-0,437	-0,890	-0,974	-0,038	-0,254	-0,151	-0,233	-0,424	-0,658	GO:0000004	unknown
6	Afu1g03380	UDP-N-acetylglucosaminyltransferase	0,029	-0,326	-0,261	-0,064	-0,525	-0,817	-0,027	-0,735	-0,067	-0,167	-0,155	-0,599	GO:0045229	cell wall and envelope biogenesis
6	Afu5g02770	hypothetical protein	-0,050	-0,723	-0,015	-0,225	-0,439	-0,364	0,095	-0,231	-0,120	-0,182	-0,046	-0,066	GO:0000004	unknown
6	Afu4g06750	protein kinase, putative	-0,046	-0,676	0,018	-0,090	-0,357	-0,497	-0,038	-0,339	-0,004	0,054	-0,172	-0,159	GO:0051301	cell division
6	Afu7g02500	polysaccharide synthase Cps1, putative	0,030	-0,725	0,488	-0,224	-0,911	-1,097	0,044	0,086	0,218	-0,007	-0,184	0,043	GO:0005975	carbohydrate metabolism
6	Afu4g01350	hypothetical protein	0,051	-0,287	0,401	-0,552	-0,986	-0,822	-0,035	-0,350	0,094	0,597	-0,338	-0,414	GO:0000004	unknown
6	Afu1g10380	nonribosomal peptide synthase Afpes1	-0,022	-0,116	0,136	0,124	-0,609	-0,976	-0,005	-0,117	0,476	0,857	0,820	0,530	GO:0019748	secondary metabolism
6	Afu3g00720	DUF500 and UBA/TS-N domain protein	0,027	0,630	-0,162	-0,655	-0,601	-0,980	0,041	0,289	0,669	0,566	0,308	0,356	GO:0000004	unknown
6	Afu2g00670	protein kinase, putative	NaN	0,060	-0,167	-0,811	-0,061	-0,540	0,075	0,217	0,115	-0,110	-0,087	-0,066	GO:0006464	protein modification
6	Afu1g17250	conidial hydrophobin RodB	-0,100	-0,026	-0,205	-0,684	-0,703	-0,874	0,155	0,178	0,430	0,101	0,226	-0,112	GO:0045229	cell wall and envelope biogenesis
6	Afu2g00500	conserved hypothetical protein	0,049	-0,271	0,180	-0,859	-0,698	-0,916	0,016	0,627	0,399	-0,083	0,097	0,053	GO:0000004	unknown
6	Afu3g14680	lysophospholipase Plb3	-0,009	-0,097	-0,469	-0,037	-0,348	-0,858	-0,006	0,190	-0,222	-0,158	0,186	0,107	GO:0006629	lipid metabolism
6	Afu7g07140	hypothetical protein	-0,057	0,395	-0,377	-0,173	-0,633	-1,043	0,247	0,037	-0,050	0,144	0,072	-0,090	GO:0000004	unknown
6	Afu5g00230	hypothetical protein	0,110	0,522	-0,277	-0,302	-0,960	-1,389	-0,001	0,186	-0,126	-0,099	-0,033	0,150	GO:0000004	unknown
6	Afu5g00190	hypothetical protein	-0,019	0,331	-0,507	-0,346	-1,008	-1,064	NaN	-0,014	-0,013	-0,291	-0,094	-0,106	GO:0000004	unknown
6	Afu6g02940	chitin synthase activator (Chs3), putative	0,134	-0,092	0,221	-0,071	-0,360	-0,582	0,041	-0,082	0,076	-0,012	-0,144	-0,204	GO:0045229	cell wall and envelope biogenesis
6	Afu4g08390	hypothetical protein	0,042	-0,097	0,148	-0,062	-0,817	-0,626	-0,085	0,196	-0,073	-0,062	-0,093	-0,177	GO:0000004	unknown
6	Afu1g17280	hypothetical protein	0,003	-0,140	-0,036	-0,435	-0,462	-0,845	0,025	0,001	0,072	0,321	0,049	-0,311	GO:0000004	unknown
6	Afu5g00170	extracellular serine treonine rich protein	NaN	-0,192	-0,221	-0,381	-0,559	-0,590	0,041	-0,121	0,026	-0,219	0,163	-0,137	GO:0000004	unknown
6	Afu6g08470	glycerol kinase, putative	-0,180	-0,119	-0,159	-0,333	-0,602	-0,627	0,028	0,137	-0,028	-0,050	-0,240	-0,037	GO:0000004	unknown
6	Afu5g12460	2-amino-3-carboxymuconate-6-semialdehyde decarboxylase, putative	NaN	-0,188	-0,346	-0,515	-0,709	-0,811	0,062	0,233	-0,092	0,064	-0,205	-0,061	GO:0000004	unknown
6	Afu5g13690	DUF74 domain protein	NaN	-0,173	-0,016	-0,024	0,029	-0,598	0,406	-0,097	-0,057	-0,020	-0,047	0,046	GO:0000004	unknown
6	Afu3g15190	periplasmic nitrate reductase, putative	0,072	0,286	0,165	-0,228	-0,310	-0,446	-0,036	0,095	0,141	0,143	0,167	-0,004	GO:0000004	unknown
6	Afu5g01550	C6 transcription factor, putative	-0,052	0,025	0,360	0,123	0,034	-0,547	-0,007	-0,005	0,104	0,164	0,084	-0,017	GO:0045449	regulation of transcription
6	Afu2g02010	transmembrane protein UsgS	-0,005	0,409	0,064	-0,056	-0,655	-0,817	0,041	0,658	0,597	0,366	-0,044	-0,248	GO:0000004	unknown
6	Afu3g03230	hypothetical protein	0,038	0,504	0,057	-0,261	-0,798	-0,613	0,069	0,364	0,517	0,478	-0,011	-0,223	GO:0000004	unknown
6	Afu2g13500	hypothetical protein	-0,023	0,591	0,215	-0,136	-0,747	-1,064	-0,046	0,561	0,380	-0,008	-0,053	-0,204	GO:0000004	unknown
6	Afu7g08300	hypothetical protein	NaN	0,420	0,292	-0,282	-0,254	-0,884	NaN	0,488	0,289	0,240	0,361	0,085	GO:0000004	unknown
6	Afu6g06560	conserved hypothetical protein	0,053	0,372	0,602	-0,215	-0,554	-0,316	-0,053	0,260	-0,011	0,214	-0,079	-0,193	GO:0000004	unknown
6	Afu8g00410	methionine aminopeptidase, type II, putative	-0,125	0,633	0,536	-0,323	-0,302	-0,662	0,137	0,357	0,268	-0,026	-0,067	-0,060	GO:0019748	secondary metabolism

6	Afu3g02870	NUDIX domain, putative	-0,143	0,502	0,628	-0,171	-0,314	-0,720	0,060	0,640	0,148	0,045	0,024	-0,433	GO:0000004	unknown
6	Afu5g09640	conserved fungal protein	0,028	0,116	0,249	-0,657	-0,940	-1,208	-0,032	0,258	-0,130	-0,546	-0,718	-0,998	GO:0000004	unknown
6	Afu3g00960	conserved hypothetical protein	0,037	0,486	0,574	-1,131	-1,199	-0,929	0,005	0,209	0,111	-0,236	-0,712	-0,757	GO:0000004	unknown
6	Afu3g09980	hypothetical protein	0,035	0,427	-0,093	-0,157	-0,748	-1,020	-0,038	0,293	-0,647	-0,007	-0,415	-0,667	GO:0000004	unknown
6	Afu4g09700	hypothetical protein	-0,053	0,237	0,278	-0,461	-0,773	-1,378	-0,036	0,075	0,008	-0,286	-0,213	-0,403	GO:0000004	unknown
6	Afu1g15160	hypothetical protein	0,012	0,599	-0,181	-0,676	-1,094	-1,102	-0,022	0,415	0,085	-0,268	-0,234	-0,194	GO:0000004	unknown
6	Afu4g12700	hypothetical protein	-0,020	0,426	-0,221	-0,425	-0,889	-1,519	0,019	0,530	0,018	-0,040	-0,270	-0,641	GO:0000004	unknown
6	Afu2g04980	tyrosine decarboxylase, putative	0,035	0,392	-0,324	-0,323	-0,801	-0,679	-0,020	0,004	0,396	0,009	-0,461	-0,597	GO:0000004	unknown
6	Afu2g12780	von Willebrand domain protein	NaN	0,477	-0,209	-0,429	-0,865	-0,669	-0,019	0,388	0,727	0,133	-0,505	-0,608	GO:0000004	unknown
6	Afu1g02570	aspartyl-tRNA synthetase, cytoplasmic	0,000	0,694	-0,685	-0,732	-0,878	-0,627	0,002	-0,244	0,002	-0,039	-0,306	-0,279	GO:0006412	protein biosynthesis
6	Afu6g13200	autophagy regulatory protein (Gsa11/Atg2), putative	0,024	0,675	-0,561	-0,738	-0,993	-0,710	-0,004	0,211	-0,038	0,102	-0,648	-0,632	GO:0006950	response to stress
6	Afu7g06100	acyl-coenzyme A oxidase I, peroxisomal, component A	-0,146	0,778	-0,450	-0,661	-0,775	-0,950	0,026	0,085	-0,080	-0,070	-0,730	-0,610	GO:0006629	lipid metabolism
6	Afu2g17220	C2H2 transcription factor (AmdX), putative	0,058	0,755	-0,449	-0,356	-0,992	-0,742	-0,004	0,379	-0,182	0,020	-0,483	-0,587	GO:0045449	regulation of transcription
6	Afu1g15940	Auxin Efflux Carrier superfamily	-0,109	0,597	-0,192	-0,471	-0,855	-0,835	0,053	-0,019	-0,030	-0,072	-0,442	-0,483	GO:0006810	transport
6	Afu1g09750	aldehyde reductase (AKR1), putative	NaN	0,499	0,051	-0,547	-0,658	-0,914	0,050	0,282	-0,030	-0,140	-0,447	-0,419	GO:0000004	unknown
6	Afu7g01340	RPEL repeat protein	0,005	0,370	-0,497	-0,704	-0,800	-0,720	0,029	0,377	-0,315	-0,254	-0,481	-0,471	GO:0000004	unknown
6	Afu2g11570	F-box domain protein	-0,051	0,562	-0,352	-0,618	-0,619	-0,327	0,058	0,276	-0,251	-0,443	-0,644	-0,234	GO:0000004	unknown
6	Afu2g02990	MYB DNA-binding domain protein	-0,028	0,516	-0,129	-0,469	-0,753	-0,617	-0,045	0,199	-0,265	-0,485	-0,616	-0,786	GO:0000004	unknown
6	Afu7g00710	oxalate/formate antiporter, putative	0,131	0,808	-0,414	-0,637	-0,650	-0,616	0,040	0,177	-0,278	-0,843	-0,630	-0,781	GO:0006810	transport
6	Afu8g00930	chitosanase, putative	NaN	0,435	0,143	-0,785	-0,668	-0,495	-0,066	0,522	-0,089	-1,232	-0,943	-0,569	GO:0045229	cell wall and envelope biogenesis
6	Afu1g03720	UPF0136 domain protein	-0,042	0,300	-0,136	-0,950	-0,899	-0,015	0,004	0,358	0,017	-0,485	-0,653	0,051	GO:0000004	unknown
6	Afu5g00700	hypothetical protein	-0,042	0,572	-0,497	-0,837	-0,636	-0,363	0,046	0,457	-0,374	-0,812	-0,335	0,116	GO:0000004	unknown
7	Afu6g07720	phosphoenolpyruvate carboxykinase (ATP)	-0,014	0,426	-1,699	0,077	-0,736	-1,004	-0,030	0,254	0,051	0,662	-0,316	-0,283	GO:0006091	energy pathways
7	Afu4g11310	fructose-1,6-bisphosphatase	-0,186	0,840	-1,461	-0,592	-0,769	-0,645	0,017	0,110	-0,276	-0,159	-0,406	-0,225	GO:0006091	energy pathways
7	Afu4g08710	short chain dehydrogenase, putative	0,016	1,005	-1,842	-0,794	-0,758	-0,869	0,017	0,993	-0,219	-0,045	-0,047	0,117	GO:0006629	lipid metabolism
7	Afu5g13970	conserved hypothetical protein	0,014	-0,537	-0,492	-0,654	-0,897	-0,760	-0,036	0,023	0,135	0,179	0,240	0,873	GO:0000004	unknown
7	Afu4g09190	S-adenosyl-methionine-sterol-C- methyltransferas	0,016	-0,062	-1,291	-0,629	-0,683	-0,175	-0,006	-0,644	0,152	-0,045	0,193	0,352	GO:0006629	lipid metabolism
7	Afu5g06580	mfs-multidrug-resistance transporter	-0,042	-0,480	-0,583	-1,112	-1,073	-1,183	0,004	-0,048	0,496	0,266	-0,421	-0,445	GO:0006810	transport
7	Afu6g01870	hypothetical protein	-0,040	-0,948	-0,834	-0,812	-1,000	-1,036	0,021	-0,115	-0,203	-0,179	-0,122	-0,093	GO:0000004	unknown
7	Afu3g03360	hypothetical protein	0,035	-0,549	-0,792	-0,992	-1,363	-1,068	0,011	-0,130	-0,009	-0,197	-0,028	0,009	GO:0000004	unknown
7	Afu2g17830	hypothetical protein	-0,009	-1,029	-1,353	-0,553	-0,983	-1,014	0,038	-0,346	-0,231	-0,215	-0,057	-0,198	GO:0000004	unknown
7	Afu3g08850	FAD dependent sulfhydryl oxidase Erv1, putative	-0,100	-0,544	-1,272	-0,804	-0,801	-0,490	-0,004	-0,222	-0,303	0,040	-0,061	-0,013	GO:0019725	homeostasis
7	Afu2g15190	ribulose-phosphate 3-epimerase	0,014	-0,601	-1,054	-0,640	-0,812	-0,630	-0,011	-0,169	-0,209	-0,202	-0,130	0,013	GO:0006091	energy pathways
7	Afu2g14530	esterase D	NaN	-0,812	-0,995	-0,840	-0,805	-0,474	0,013	0,067	0,048	-0,287	-0,155	0,064	GO:0006629	lipid metabolism
7	Afu3g03210	hypothetical protein	NaN	-0,757	-1,268	-0,750	-0,817	-0,609	0,025	-0,047	0,016	-0,277	-0,110	0,054	GO:0000004	unknown
7	Afu7g00330	Esterase/Lipase family protein	-0,057	-0,335	-1,411	-0,927	-0,892	-0,752	-0,040	-0,074	-0,205	-0,087	-0,200	-0,277	GO:0006629	lipid metabolism
7	Afu5g14130	hypothetical protein	0,043	-0,531	-1,612	-0,601	-0,843	-0,572	0,038	-0,300	-0,262	-0,513	-0,089	-0,214	GO:0000004	unknown
7	Afu3g04220	fatty acid synthase beta subunit, putative	-0,002	-0,346	-0,527	-1,029	-0,677	-0,070	-0,042	-0,316	-0,203	-0,163	-0,192	-0,186	GO:0006629	lipid metabolism
7	Afu3g07400	hypothetical protein	0,026	-0,914	-0,544	-0,627	-0,478	-0,420	-0,002	-0,233	-0,306	-0,049	-0,095	0,120	GO:0000004	unknown
7	Afu5g07840	conserved hypothetical protein	NaN	-0,703	-0,896	-0,821	-0,571	-0,535	-0,001	0,328	-0,111	-0,621	-0,519	0,062	GO:0000004	unknown
7	Afu2g11590	hypothetical protein	NaN	-0,525	-0,835	-0,784	-1,133	-0,577	-0,063	-0,148	-0,198	-0,566	-0,543	-0,486	GO:0000004	unknown
7	Afu2g13060	calcineurin binding protein, putative	-0,005	-0,746	-0,991	-0,737	-0,797	-0,595	0,032	-0,499	-0,283	-0,549	-0,374	-0,363	GO:0006464	protein modification
7	Afu5g12560	hypothetical protein	-0,091	-0,209	-0,661	-0,508	-0,856	-0,805	0,082	0,062	-0,570	-0,103	-0,419	-0,500	GO:0000004	unknown
7	Afu3g06030	ubiquitin conjugating enzyme (UbcD), putative	0,018	-0,120	-0,610	-0,906	-0,882	-0,518	0,025	0,059	-0,219	-0,401	-0,704	-0,234	GO:0006464	protein modification
7	Afu1g14450	exo-beta-1,3-glucanase Exg0	NaN	-0,188	-0,532	-0,825	-0,791	-0,577	0,133	-0,239	-0,279	-0,571	-0,208	-0,295	GO:0045229	cell wall and envelope biogenesis
7	Afu3g03380	maltose O-acetyltransferase, putative	NaN	-0,349	-0,735	-0,885	-0,933	-0,740	0,222	-0,015	-0,356	-0,451	-0,353	-0,271	GO:0019748	secondary metabolism
7	Afu4g13990	hypothetical protein	NaN	0,012	-0,590	-0,649	-0,866	-0,826	-0,050	0,005	-0,461	-0,614	-0,365	-0,466	GO:0000004	unknown
7	Afu5g09420	CLOCK-CONTROLLED PROTEIN 8	0,169	0,075	-0,669	NaN	-0,695	-0,595	-0,017	-0,466	-0,469	-0,242	-0,328	-0,421	GO:0000004	unknown
7	Afu5g07410	phenylacetyl-CoA ligase	0,188	-0,390	-0,340	-0,481	-0,758	-0,370	0,032	-0,384	-0,349	-0,370	-0,457	-0,320	GO:0000004	unknown
7	Afu5g09930	hypothetical protein	0,073	-0,083	-1,003	-0,971	-0,650	-0,611	0,056	0,145	-0,476	-0,296	-0,166	-0,182	GO:0000004	unknown
7	Afu2g10920	enoyl-CoA hydratase/isomerase family protein	0,218	-0,433	-1,065	-0,877	-0,599	-0,613	0,045	-0,156	-0,367	-0,475	-0,275	-0,159	GO:0006629	lipid metabolism
7	Afu5g09290	NAD-dependent 15-hydroxyprostaglandin dehydrogenase	0,724	-0,188	-0,754	NaN	-0,477	-0,568	0,054	-0,077	-0,449	-0,397	-0,440	-0,218	GO:0006732	coenzyme metabolism
7	Afu4g14290	conserved hypothetical protein	NaN	0,074	-0,509	-0,923	-0,423	-0,703	0,195	-0,037	-0,418	-0,244	-0,249	-0,205	GO:0000004	unknown
7	Afu7g00550	NACHT and WD40 domain protein	-0,234	-0,534	-0,293	-0,531	-1,089	-1,056	0,028	-0,231	-0,063	-0,248	-0,236	-0,282	GO:0000004	unknown
7	Afu3g11970	C2H2 transcription factor PacC, putative	-0,070	-0,858	-0,053	-0,293	-0,909	-0,982	-0,001	-0,480	0,026	-0,140	-0,293	-0,404	GO:0045449	regulation of transcription
7	Afu6g14470	hypothetical protein	-0,068	-0,391	-0,692	-0,176	-0,569	-0,780	0,045	-0,244	-0,191	0,113	-0,178	-0,345	GO:0000004	unknown
7	Afu6g07730	plasma membrane antiporter, putative	-0,093	-0,398	-0,415	-0,249	-0,927	-0,830	-0,009	-0,298	-0,278	-0,132	-0,405	-0,506	GO:0019725	homeostasis
7	Afu2g08660	conserved hypothetical protein	-0,067	-0,319	-0,432	-0,171	-0,697	-0,822	-0,010	-0,196	-0,462	-0,139	-0,290	-0,357	GO:0000004	unknown
7	Afu4g10210	phospholipid-transporting ATPase (DRS2), putative	-0,068	-0,154	-0,623	-0,388	-0,514	-0,690	-0,028	-0,383	0,040	-0,304	-0,032	-0,234	GO:0006810	transport
7	Afu3g07570	conserved hypothetical protein	-0,029	-0,102	-0,610	-0,123	-0,645	-0,756	0,073	-0,026	-0,200	-0,158	-0,030	-0,089	GO:0000004	unknown
7	Afu1g15420	hypothetical protein	NaN	-0,673	-0,581	-0,610	-0,836	-0,723	-0,011	-0,104	-0,088	-0,211	-0,312	-0,189	GO:0000004	unknown
7	Afu6g10290	receptor L domain protein	0,065	-0,561	-0,660	-0,636	-0,919	-0,988	0,010	-0,061	-0,095	-0,352	-0,375	-0,505	GO:0045229	cell wall and envelope biogenesis
7	Afu1g03790	mannosylphosphorylation protein (Mnn4), putative	-0,002	-0,547	-0,428	-0,515	-0,655	-0,600	-0,017	-0,039	-0,208	-0,265	-0,356	-0,537	GO:0006464	protein modification
7	Afu5g07980	hypothetical protein	0,051	-0,516	-0,300	-0,667	-0,686	-0,564	0,071	-0,021	-0,051	-0,076	-0,065	-0,097	GO:0000004	unknown
7	Afu8g00710	secreted antimicrobial peptide, putative	NaN	-0,339	-0,493	-0,576	-0,467	-0,304	-0,074	0,177	0,074	-0,128	-0,255	0,002	GO:0019748	secondary metabolism
7	Afu1g09900	alpha-L-arabinofuranosidase	-0,143	-0,214	-0,472	NaN	-0,671	-0,649	0,035	0,130	0,027	-0,118	-0,337	NaN	GO:0000004	unknown
7	Afu6g03200	solute symporter family transporter	-0,068	0,456	-0,971	-0,819	-1,039	-0,694	0,050	0,164	-0,062	-0,101	-0,052	-0,181	GO:0006810	transport
7	Afu7g00320	extracelular proline-glycine rich protein	0,026	-0,069	-0,680	-0,862	-1,001	-0,673	-0,055	-0,067	0,097	0,157	0,092	-0,073	GO:0000004	unknown
7	Afu5g00220	hypothetical protein	0,009	0,016	-0,553	-0,885	-1,494	-0,562	-0,064	-0,116	-0,105	-0,157	-0,117	-0,302	GO:0000004	unknown
7	Afu3g03700	Sugar transporter subfamily	0,149	-0,389	-1,059	-0,511	-0,750	-0,700	0,096	0,028	0,254	0,021	-0,281	-0,271	GO:0006810	transport
7	Afu1g16110	arsenic methyltransferase Cyt19, putative	0,028	-0,192	-1,245	-0,816	-0,924	-0,737	0,037	0,273	0,144	-0,136	-0,286	-0,180	GO:0006950	response to stress
7	Afu6g07370	hypothetical protein	-0,006	-0,088	-0,562	-0,642	-0,950	-1,199	0,021	0,335	0,070	-0,066	-0,135	-0,291	GO:0000004	unknown

7	Afu5g12350	ribonucleotide reductase	0,008	-0,040	-0,678	-0,516	-0,957	-1,081	-0,038	-0,174	0,129	0,273	-0,245	-0,667	GO:0006259	DNA metabolism
7	Afu3g00930	C6 transcription factor, putative	0,149	-0,045	-0,574	-0,429	-0,760	-0,782	-0,045	-0,054	-0,106	0,076	-0,257	-0,455	GO:0045449	regulation of transcription
7	Afu1g13300	GTP cyclohydrolase ii	0,027	0,201	-0,747	-0,740	-0,478	-0,212	-0,056	-0,049	0,067	-0,313	-0,199	-0,123	GO:0006732	coenzyme metabolism
7	Afu5g07290	NADPH-dependent FMN and FAD containing oxidoreductase	-0,083	-0,227	-1,138	-0,694	-0,499	-0,245	0,013	0,023	0,069	-0,408	-0,534	-0,153	GO:0000004	unknown
7	Afu2g14390	methyltransferase, putative	0,024	-0,232	-0,853	-0,636	-0,554	-0,383	0,025	0,011	-0,343	-0,525	-0,152	-0,258	GO:0000004	unknown
7	Afu4g12270	androgen receptor coactivator ARA55	0,039	-0,134	-1,092	-0,573	-0,610	-0,197	0,077	-0,047	-0,282	-0,248	-0,289	-0,144	GO:0000004	unknown
7	Afu2g13620	thiamine pyrophosphate enzyme, putative	-0,137	0,231	-0,838	-0,216	-0,513	-0,416	0,085	0,091	-0,304	-0,096	-0,145	0,016	GO:0000004	unknown
8	Afu5g07330	carboxypeptidase S1, putative	-0,019	NaN	-1,054	-1,216	-0,569	-0,366	-0,005	-0,878	-0,639	-0,570	-0,693	-0,253	GO:0030163	protein catabolism
8	Afu3g12690	conserved hypothetical protein	0,009	-1,272	-0,431	-1,066	-0,697	-0,170	-0,009	-0,985	-0,546	-0,691	-0,968	-0,533	GO:0000004	unknown
8	Afu3g10090	dihydroneopterin aldolase domain protein	-0,047	-0,868	-0,707	-0,843	-0,556	-0,220	-0,002	-0,575	-0,845	-1,094	-0,961	-0,624	GO:0000004	unknown
8	Afu1g15780	3-isopropylmalate dehydrogenase	-0,002	-0,570	-1,205	-0,943	-0,783	-0,113	-0,012	-0,433	-0,637	-1,105	-0,745	-0,372	GO:0006519	amino acid
8	Afu5g06710	DUF89 domain protein	-0,058	-0,629	-0,931	-0,959	-0,508	0,243	-0,024	-0,388	-0,860	-1,237	-1,088	-0,373	GO:0000004	unknown
8	Afu4g11340	saccharopine dehydrogenase	-0,011	-0,480	-0,831	-1,465	-1,037	-0,397	-0,012	-0,132	-0,222	-0,574	-1,072	-0,258	GO:0006519	amino acid
8	Afu5g09860	esterase, putative	0,040	-0,415	-1,035	-1,572	-1,233	-0,300	-0,020	0,163	-0,496	-1,238	-1,062	0,045	GO:0006629	lipid metabolism
8	Afu2g09790	glucose-6-phosphate isomerase	-0,067	0,468	-0,080	-1,285	-0,853	-0,040	0,005	0,272	-0,295	-1,123	-0,969	-0,326	GO:0006091	energy pathways
8	Afu2g13590	hypothetical protein	0,027	0,021	-0,627	-0,891	-0,735	-0,234	-0,028	-0,078	-0,909	-1,230	-1,015	-0,547	GO:0000004	unknown
8	Afu2g03870	FKBP-type peptidyl-prolyl isomerase, putative	-0,012	-0,267	-0,729	-1,162	-0,798	-0,273	-0,003	-0,123	-0,638	-0,916	-0,928	-0,512	GO:0000004	unknown
8	Afu1g09470	aminotransferase, class V, putative	-0,046	-0,182	-0,871	-0,901	-0,883	-0,332	-0,004	-0,185	-0,566	-0,802	-1,070	-0,641	GO:0006091	energy pathways
8	Afu4g12730	Phosphoserine phosphatase, putative	-0,033	-0,114	-0,635	-0,912	-0,756	-0,339	-0,012	-0,217	-0,445	-0,889	-0,942	-0,705	GO:0000004	unknown
8	Afu3g10300	galactokinase	0,070	-0,152	-0,577	-1,082	-0,958	-0,187	-0,093	-0,242	-0,614	-1,077	-1,191	-0,478	GO:0005975	carbohydrate metabolism
8	Afu7g01580	molybdopterin synthase small subunit CnxG	-0,107	-0,029	-0,581	-0,779	-0,463	-0,194	-0,005	0,014	-0,655	-0,667	-0,661	-0,421	GO:0006732	coenzyme metabolism
8	Afu6g13310	26S proteasome non-ATPase regulatory subunit 9, putative	-0,023	-0,038	-0,809	-0,915	-0,451	-0,180	-0,038	0,098	-0,614	-0,589	-0,679	-0,492	GO:0030163	protein catabolism
8	Afu2g03970	DUF52 domain protein	0,061	0,249	-0,605	-0,821	-0,792	-0,336	0,039	0,103	-0,562	-0,584	-0,802	-0,451	GO:0000004	unknown
8	Afu2g00280	hypothetical protein	-0,041	0,099	-0,756	-0,796	-0,915	-0,567	-0,044	0,005	-0,565	-1,100	-0,681	-0,458		
8	Afu5g02820	hypothetical protein	0,078	-0,251	-0,928	-0,675	-0,649	-0,494	-0,011	-0,004	-0,645	-1,050	-0,564	-0,701	GO:0000004	unknown
8	Afu1g12940	MAP kinase (Osm1), putative	-0,012	0,071	-0,910	-0,423	-0,695	-0,484	-0,012	-0,069	-0,580	-0,956	-0,536	-0,474	GO:0007165	signal transduction
8	Afu3g05650	alpha,alpha-trehalose-phosphate synthase subunit TPS2, putative	-0,014	0,075	-0,989	-0,761	-0,486	-0,075	-0,001	-0,463	-0,560	-0,695	-0,543	-0,317	GO:0005975	carbohydrate metabolism
8	Afu2g14960	thioredoxin, putative	0,002	0,117	-1,086	-0,914	-0,612	-0,072	0,053	-0,260	-0,644	-0,820	-0,444	-0,187	GO:0006950	response to stress
8	Afu2g11560	galactose-1-phosphate uridylyltransferase	-0,130	-0,096	-0,892	-0,529	-0,359	-0,050	-0,067	-0,131	-0,573	-0,861	-0,539	-0,473	GO:0005975	carbohydrate metabolism
8	Afu4g13500	aldehyde dehydrogenase, putative	-0,004	-0,251	-0,780	-0,789	-0,492	-0,028	0,050	-0,369	-0,634	-0,685	-0,643	-0,318	GO:0000004	unknown
8	Afu7g01370	thiosulfate sulfurtransferase, putative	0,050	-0,404	-1,064	-0,683	-0,322	0,173	-0,047	-0,307	-0,740	-0,569	-0,671	-0,402	GO:0000004	unknown
8	Afu2g16530	cyanate hydratase, putative	0,120	0,008	-0,793	-1,095	-0,413	-0,022	-0,006	-0,243	-1,028	-1,293	-0,819	-0,150	GO:0006950	response to stress
8	Afu3g09120	hypothetical protein	-0,211	0,582	-0,745	-0,710	-0,376	-0,245	-0,035	-0,018	-0,873	-0,965	-0,791	-0,430	GO:0000004	unknown
8	Afu3g12270	glutathione peroxidase family protein	0,004	1,046	-0,620	-1,142	-0,511	0,218	-0,056	0,101	-0,799	-0,688	-0,523	-0,283	GO:0006950	response to stress
8	Afu2g09030	secreted dipeptidyl peptidase	-0,229	0,153	-0,299	-0,451	-0,414	-0,710	-0,006	-0,265	-0,779	-1,143	-0,730	-0,791	GO:0030163	protein catabolism
8	Afu1g13340	DUF6 domain protein, putative	0,074	0,294	-0,562	-0,610	-0,418	-0,789	-0,009	-0,046	-0,779	-0,561	-0,485	-0,469	GO:0000004	unknown
8	Afu1g09740	hypothetical protein	0,237	0,111	-0,400	-0,369	-0,588	-0,503	-0,066	0,005	-0,348	-0,637	-0,456	-0,597	GO:0000004	unknown
8	Afu6g03140	isp4 protein, putative	0,171	-0,175	-0,146	-0,549	-0,530	-0,308	0,013	0,050	-0,622	-0,304	-0,467	-0,598	GO:0006810	transport
8	Afu7g02390	hypothetical protein	0,024	-0,563	-0,718	-0,891	-1,111	-0,515	0,029	-0,416	-0,659	-0,617	-0,357	-0,276	GO:0000004	unknown
8	Afu6g03530	glutamine synthetase	-0,009	-0,136	-1,083	-1,041	-0,862	-0,451	-0,016	-0,310	-0,688	-0,353	-0,894	-0,552	GO:0006519	amino acid
8	Afu1g12370	DUF255 domain protein	-0,033	-0,406	-1,101	-0,622	-0,812	-0,550	-0,066	-0,309	-0,482	-0,722	-0,679	-0,419	GO:0000004	unknown
8	Afu2g08950	isochorismatase family hydrolase, putative	-0,030	-0,214	-1,114	-0,879	-0,952	-0,547	0,010	-0,234	-0,640	-0,731	-0,764	-0,515	GO:0000004	unknown
8	Afu3g07150	succinate-semialdehyde dehydrogenase, putative	0,003	-0,611	-1,115	-1,041	-0,741	-0,515	0,090	-0,169	-0,402	-0,830	-0,548	-0,362	GO:0006519	amino acid
8	Afu4g07050	L-lactate dehydrogenase	-0,010	-0,788	-1,178	-0,853	-0,849	-0,449	-0,018	-0,449	-0,403	-0,781	-0,667	-0,370	GO:0006091	energy pathways
8	Afu4g11460	aminotransferase, classes I and II, putative	-0,050	-0,567	-1,138	-1,087	-0,961	-0,636	-0,044	-0,363	-0,379	-0,640	-0,549	-0,569	GO:0000004	unknown
8	Afu6g13700	hypothetical protein	-0,015	-0,545	-1,166	-0,951	-1,097	-0,579	0,030	-0,254	-0,469	-0,583	-0,641	-0,386	GO:0000004	unknown
8	Afu5g12130	Rab small monomeric GTPase Rab7, putative	-0,094	-0,161	-1,454	-0,682	-0,735	-0,469	0,034	-0,147	-0,502	-0,571	-0,549	-0,277	GO:0046951	intracellular transport
8	Afu2g11070	tyrosyl-DNA phosphodiesterase, putative	NaN	-0,383	-0,805	-0,688	-0,831	-0,141	0,005	-0,498	-0,603	-0,558	-0,935	-0,689	GO:0006259	DNA metabolism
8	Afu5g03600	nucleotide binding protein, putative	-0,020	-0,401	-0,922	-0,731	-0,764	-0,288	0,015	-0,570	-0,731	-0,932	-0,804	-0,609	GO:0000004	unknown
8	Afu5g08650	conserved hypothetical protein	0,032	-0,460	-0,959	-0,620	-0,612	-0,387	-0,056	-0,617	-0,436	-0,949	-0,672	-0,553	GO:0000004	unknown
8	Afu1g09520	hypothetical protein	NaN	-0,622	-0,675	-0,532	-0,646	-0,446	0,049	-0,374	-0,393	-0,551	-0,613	-0,494	GO:0000004	unknown
8	Afu2g11600	oxidoreductase, putative	0,028	-0,537	-0,916	-0,712	-0,681	-0,414	0,004	-0,247	-0,421	-0,655	-0,620	-0,634	GO:0000004	unknown
8	Afu5g03150	hypothetical protein	NaN	-0,546	-1,136	-0,476	-0,477	-0,388	-0,006	-0,333	-0,684	-0,496	-0,548	-0,397	GO:0000004	unknown
8	Afu7g06120	transmembrane transporter, putative	-0,037	-0,426	-1,091	-0,691	-0,465	-0,490	0,069	-0,172	-0,292	-0,599	-0,393	-0,419	GO:0006810	transport
8	Afu4g06520	conserved hypothetical protein	-0,130	-0,376	-1,054	-0,556	-0,373	-0,412	-0,007	-0,451	-0,371	-0,490	-0,393	-0,450	GO:0000004	unknown
8	Afu2g02810	alcohol dehydrogenase, zinc-containing, putative	-0,052	-0,402	-0,434	-0,505	-0,673	-0,336	-0,056	-0,218	-0,209	-0,767	-0,782	-0,527	GO:0005975	carbohydrate metabolism
8	Afu2g10220	glycerol dehydrogenase, putative	-0,068	-0,340	-0,693	-0,789	-0,726	-0,506	-0,008	-0,022	-0,250	-0,795	-0,730	-0,413	GO:0000004	unknown
8	Afu6g12820	MAP kinase FUS3/KSS1	-0,023	-0,375	-0,104	-0,492	-0,610	-0,206	0,066	-0,312	-0,471	-0,703	-0,550	-0,438	GO:0007165	signal transduction
8	Afu3g08650	C1-THFS protein	-0,026	0,085	-0,263	-0,886	-0,470	0,071	-0,008	-0,107	-0,021	-0,682	-0,737	-0,292	GO:0006139	nucleobase, nucleoside, nucleotide and nucleic acid metabolism
8	Afu1g14850	acyl-CoA dehydrogenase, putative	-0,184	0,186	-0,047	-0,435	-0,784	0,055	-0,053	-0,107	-0,541	-0,577	-0,746	-0,071	GO:0006629	lipid metabolism
8	Afu2g12450	hydroxymethylglutaryl-CoA lyase	-0,057	-0,252	-0,553	-0,563	-0,748	-0,083	-0,044	-0,227	-0,567	-0,293	-0,682	-0,253	GO:0006629	lipid metabolism
8	Afu6g12680	HIT domain protein	-0,011	-0,204	-0,576	-1,103	-0,753	-0,093	-0,050	0,022	-0,309	-0,312	-0,762	-0,019	GO:0000004	unknown
8	Afu6g03520	short-chain dehydrogenase/reductase family protein, putative	-0,012	0,069	-0,525	-0,821	-0,653	0,211	-0,012	-0,200	-0,333	-0,004	-0,887	0,035	GO:0000004	unknown
9	Afu6g10660	ATP-citrat-lyase	0,035	-1,322	-2,021	0,476	-0,749	-0,271	-0,031	-1,396	-1,719	-0,562	-0,539	-0,531	GO:0006629	lipid metabolism
9	Afu4g10230	conserved hypothetical protein	-0,017	-0,817	-1,561	-0,206	-0,723	-0,890	0,066	-1,347	-0,658	-0,922	-0,275	-0,996	GO:0000004	unknown
9	Afu6g10650	ATP citrate lyase, subunit 1, putative	-0,003	-1,426	-1,191	0,475	-0,359	-0,040	-0,027	-0,997	-0,944	0,064	-0,258	-0,152	GO:0006629	lipid metabolism
9	Afu5g04220	mitochondrial DNA replication protein (Yhm2), putative	0,004	-1,159	-1,177	0,262	-0,642	-0,759	0,016	-0,648	-0,512	0,614	-0,329	-0,193	GO:0006259	DNA metabolism
9	Afu3g08980	threonine synthase	-0,037	-1,227	-0,905	-0,756	0,003	0,224	-0,041	-0,778	-0,006	-0,626	-0,457	-0,103	GO:0006519	amino acid
9	Afu2g03990	hypothetical protein	-0,033	-0,914	-0,829	-0,440	-0,308	-0,119	-0,005	-1,037	-0,117	0,481	-0,417	-0,332	GO:0000004	unknown
9	Afu5g03690	CRAL/TRIO domain protein	0,007	-1,662	-0,626	-0,390	-0,285	-0,025	-0,023	-1,236	0,021	0,143	-0,218	-0,024	GO:0006810	transport
9	Afu2g05670	negative differentiation regulator (Nrd1), putative	0,011	-1,689	-0,327	-0,269	-0,519	-0,439	-0,044	-0,992	-0,102	-0,205	-0,328	-0,376	GO:0016070	RNA metabolism
9	Afu3g05480	cystathionine gamma-synthase, putative	0,004	-1,236	-0,392	-0,523	-0,606	-0,420	-0,031	-0,687	0,125	-0,204	-0,573	-0,554	GO:0006519	amino acid

9	Afu2g16440	hypothetical protein	-0,019	-0,727	-1,495	-0,472	-0,536	0,130	0,036	-1,370	-0,532	-0,542	-0,174	0,546	GO:0000004	unknown
9	Afu4g13380	conserved hypothetical protein	-0,038	-0,666	-1,937	-0,571	-0,353	-0,167	-0,036	-0,776	-0,848	-0,743	0,345	-0,003	GO:0000004	unknown
9	Afu5g03340	hypothetical protein	-0,019	-0,628	-1,657	-0,644	-0,510	0,035	-0,040	-0,672	-0,582	-0,561	-0,385	-0,066	GO:0000004	unknown
9	Afu2g02740	hypothetical protein	0,055	-0,954	-1,213	-0,716	-0,427	-0,092	0,001	-0,718	-0,334	-0,595	-0,106	0,169	GO:0000004	unknown
9	Afu3g03280	FAD binding monooxygenase, putative	0,097	-0,731	-1,422	-0,490	-0,460	-0,635	0,020	-0,692	-0,375	-0,265	-0,270	-0,072	GO:0000004	unknown
9	Afu8g06730	endo-polygalacturonase D	NaN	-0,725	-1,122	-0,655	-0,727	-0,543	-0,037	-0,991	-0,599	-0,739	-0,276	-0,343	GO:0005975	carbohydrate metabolism
9	Afu3g13440	stomatin family protein	0,014	-0,506	-1,507	0,116	0,225	0,448	0,008	-1,263	-0,763	-0,737	-0,350	-0,146	GO:0000004	unknown
10	Afu2g03120	cell wall glucanase (Utr2), putative	0,054	-0,103	-2,464	-0,786	0,236	0,462	-0,086	-0,302	-2,087	-1,954	-0,745	-0,860	GO:0045229	cell wall and envelope biogenesis
10	Afu6g04460	phosphatidyl synthase	0,028	-0,155	-1,988	-0,752	0,155	0,271	-0,028	-0,452	-1,657	-1,493	-0,398	-0,536	GO:0006629	lipid metabolism
10	Afu2g10370	Iron-sulfur cluster assembly accessory protein subfamily, putative	-0,005	0,076	-1,827	-0,389	0,547	0,611	-0,022	-0,273	-1,580	-1,550	-0,343	-0,694	GO:0006464	protein modification
10	Afu8g01260	hypothetical protein	0,012	-0,787	-1,517	-0,026	-0,116	-0,196	-0,072	-0,797	-1,984	-1,250	-0,485	-0,462	GO:0000004	unknown
10	Afu5g10210	hypothetical protein	NaN	-0,380	-2,435	-0,483	-0,134	-0,137	0,039	-1,207	-1,586	-1,332	-0,301	-1,149	GO:0000004	unknown
10	Afu2g01890	CAT5 protein	0,071	0,803	-1,918	-0,768	-0,034	0,571	-0,007	-0,099	-1,385	-1,311	-0,613	-0,340	GO:0006732	coenzyme metabolism
10	Afu4g07030	hypothetical protein	0,028	1,146	-2,146	0,093	-0,031	0,413	0,022	0,169	-1,252	-0,802	-0,684	-0,093	GO:0000004	unknown
10	Afu5g09330	CipC protein	0,048	0,166	-1,034	-0,749	-0,109	1,201	-0,133	-0,359	-0,638	-0,853	-1,061	-0,287	GO:0000004	unknown
10	Afu5g11030	acyltransferase, putative	NaN	-0,149	-1,471	0,018	-0,633	-0,145	-0,072	-0,800	-1,167	-0,786	-0,956	-0,661	GO:0006629	lipid metabolism
10	Afu6g02460	glucosamine 6-phosphate acetyltransferase, putative	-0,019	-0,220	-1,368	-0,794	-0,372	-0,053	0,017	-0,387	-0,970	-1,116	-0,707	-0,666	GO:0005975	carbohydrate metabolism
10	Afu3g10940	peroxisomal membrane protein receptor Pex19, putative	0,042	-0,131	-1,552	-0,785	-0,483	-0,200	0,041	-0,612	-1,156	-1,212	-0,602	-0,531	GO:0000004	unknown
10	Afu3g15230	general amidase, putative	NaN	-0,374	-1,515	-0,611	-0,278	-0,401	0,049	-0,471	-0,977	-1,177	-0,694	-0,804	GO:0019748	secondary metabolism
10	Afu4g13590	hypothetical protein	0,050	-0,278	-1,118	-0,520	-0,229	0,179	0,140	-0,492	-0,880	-0,958	-0,583	-0,394	GO:0000004	unknown
10	Afu3g07230	DNA / pantothenate metabolism flavoprotein, putative	0,027	0,127	-1,683	-0,749	-0,187	0,415	-0,001	-0,680	-1,130	-1,066	-0,745	-0,541	GO:0006732	coenzyme metabolism
10	Afu3g14660	hypothetical protein	0,052	-0,152	-1,214	-0,260	0,237	-0,229	-0,040	-0,315	-1,218	-1,160	-0,377	-1,019	GO:0000004	unknown
10	Afu5g14110	hypothetical protein	NaN	-0,123	-1,138	-0,158	-0,068	-0,190	0,001	-0,647	-1,204	-1,153	-0,445	-0,861	GO:0000004	unknown
10	Afu5g14860	cytochrome P450	NaN	-0,109	-1,310	-0,187	-0,010	0,131	-0,059	-0,478	-1,276	-1,309	-0,510	-0,837	GO:0000004	unknown
10	Afu5g02500	hypothetical protein	-0,017	0,279	-1,137	-0,359	-0,538	-0,419	0,055	-0,092	-0,605	-1,041	-0,484	-0,601	GO:0000004	unknown
10	Afu5g11690	related to protein tyrosine phosphatase PPS1	-0,086	0,427	-1,526	-0,529	-0,566	-0,399	0,033	-0,415	-1,081	-0,662	-0,685	-0,397	GO:0006732	coenzyme metabolism
10	Afu5g09450	isoflavone reductase family protein	0,148	0,777	-1,183	-0,498	-0,495	-0,326	-0,091	-0,344	-0,742	-0,823	-0,708	-0,411	GO:0000004	unknown
10	Afu6g14280	hypothetical protein	-0,011	0,406	-1,368	0,032	-0,194	-0,083	-0,018	-0,573	-0,640	-0,977	-0,407	-0,346	GO:0000004	unknown
10	Afu2g14520	hydrolase, putative	NaN	-0,233	-1,804	-0,202	-0,126	0,012	0,047	-0,757	-0,767	-0,756	-0,221	-0,427	GO:0000004	unknown
10	Afu5g14780	heat shock trehalose synthase, putative	-0,097	-0,106	-1,817	-0,423	-0,397	-0,404	0,037	-0,254	-0,595	-0,555	-0,211	-0,396	GO:0005975	carbohydrate metabolism
10	Afu1g14610	smp2 protein	0,015	0,281	-1,783	-0,476	-0,075	0,418	-0,008	-0,628	-0,990	-0,613	-0,469	-0,201	GO:0006629	lipid metabolism
10	Afu5g08370	polyubiquitin binding protein (Doa1/Ufd3), putative	0,035	0,208	-1,870	-0,614	-0,034	0,634	-0,053	-0,333	-0,830	-0,438	-0,428	0,010	GO:0030163	protein catabolism
10	Afu6g02090	ATP synthase subunit E, putative	-0,015	0,175	-1,310	-0,431	-0,114	0,237	-0,029	-0,341	-0,904	-0,706	-0,251	-0,280	GO:0006810	transport
10	Afu5g10650	pyridoxamine phosphate oxidase, putative	0,020	0,303	-1,462	-0,349	-0,065	0,397	-0,029	-0,194	-0,828	-0,759	-0,358	-0,218	GO:0006629	lipid metabolism
10	Afu1g12090	cation efflux family protein family	0,152	-0,021	-1,353	-0,103	0,079	0,359	-0,031	-0,200	-1,019	-0,663	-0,331	-0,171	GO:0006810	transport
10	Afu4g06240	progesterone binding protein, putative	-0,039	0,233	-0,766	-0,749	-0,225	0,142	-0,058	-0,044	-1,052	-1,112	-1,012	-0,404	GO:0006629	lipid metabolism
10	Afu1g02140	glycogen debranching enzyme, putative	0,007	0,790	-0,634	-0,762	-0,350	0,026	0,070	-0,135	-1,029	-0,997	-0,509	-0,237	GO:0006091	energy pathways
10	Afu6g08940	hypothetical protein	0,019	0,523	-0,937	-0,106	-0,091	0,058	-0,042	-0,021	-0,771	-0,974	-0,251	-0,186	GO:0000004	unknown
10	Afu2g10030	vip1 protein	-0,123	0,244	-0,950	-0,282	-0,242	0,438	-0,019	0,107	-0,809	-0,940	-0,594	0,054	GO:0051301	cell division
10	Afu4g06940	Des-1 protein	0,012	0,367	-1,694	-0,792	-0,502	-0,291	0,035	0,057	-0,962	-0,952	-0,595	-0,540	GO:0006629	lipid metabolism
10	Afu4g09810	hypothetical protein	0,032	0,363	-1,466	-0,959	-0,527	-0,244	0,027	0,068	-1,246	-1,373	-0,866	-0,793	GO:0000004	unknown
10	Afu4g09800	conserved hypothetical protein	0,022	0,630	-1,467	-1,048	-0,448	0,061	0,005	0,054	-1,132	-1,259	-0,718	-0,598	GO:0000004	unknown
10	Afu4g09590	hypothetical protein	0,008	0,759	-1,304	-0,806	-0,649	-0,471	-0,032	0,018	-1,058	-1,371	-0,875	-0,809	GO:0000004	unknown
10	Afu1g05720	c-14 sterol reductase	-0,053	0,841	-0,965	-0,857	-0,831	-0,804	0,039	0,007	-1,187	-1,503	-0,879	-0,880	GO:0006629	lipid metabolism
10	Afu4g09060	phosphatidate cytidylyltransferase, putative	-0,024	0,809	-1,945	-0,744	-0,817	-0,792	0,008	-0,795	-1,227	-1,076	-0,488	-0,881	GO:0000004	unknown
10	Afu7g06770	hypothetical protein	0,029	1,182	-1,161	-0,055	-0,622	-0,381	-0,029	0,391	-0,732	-1,502	-0,840	-0,344	GO:0000004	unknown
11	Afu5g01680	MFS transporter, putative	0,157	-1,057	-2,131	-1,082	-1,102	-0,743	-0,018	-1,051	-0,924	0,276	-0,487	-0,868	GO:0006810	transport
11	Afu2g01010	myo-inositol-phosphate synthase, putative	0,013	-0,925	-2,727	-1,702	-0,217	0,557	-0,026	-0,801	-1,748	-1,329	-0,778	-0,101	GO:0005975	carbohydrate metabolism
11	Afu3g10460	nuclear transport factor 2	-0,027	-0,363	-2,307	-0,942	-0,834	-0,256	-0,043	-1,033	-1,519	-1,716	-1,161	-1,113	GO:0046907	intracellular transport
11	Afu8g02440	C-4 methyl sterol oxidase, putative	0,012	0,429	-2,852	-1,589	-0,904	-0,424	0,011	-0,647	-1,275	-1,542	-0,955	-0,896	GO:0006629	lipid metabolism
11	Afu2g15660	aldehyde dehydrogenase family protein, putative	-0,037	-0,564	-1,305	-0,917	-0,607	0,156	-0,007	-1,036	-1,155	-1,111	-0,917	-0,506	GO:0000004	unknown
11	Afu1g10120	ABC transporter permease protein	-0,035	-0,474	-1,793	-0,566	-0,541	-0,368	0,096	-0,824	-0,794	-1,198	-0,419	-0,584	GO:0000004	unknown
11	Afu8g01820	hypothetical protein	NaN	-0,561	-1,779	-0,672	-0,394	-0,401	0,066	-0,999	-1,085	-1,341	-0,761	-1,027	GO:0000004	unknown
11	Afu1g09510	GPI anchored serine-threonine rich protein	-0,031	-1,155	-1,643	-0,993	-0,915	-1,192	-0,030	-0,743	-1,368	-1,420	-1,177	-1,274	GO:0000004	unknown
11	Afu1g03490	conserved hypothetical protein	0,029	-1,459	-1,363	-1,231	-0,987	-0,493	0,016	-1,194	-0,890	-0,818	-0,949	-0,714	GO:0000004	unknown
11	Afu1g11480	hypothetical protein	0,001	-0,188	-0,913	-1,095	-1,244	-0,171	-0,032	-0,681	-1,098	-1,202	-1,417	-1,070	GO:0000004	unknown
11	Afu4g11080	acetyl-coenzyme a synthetase	0,063	-0,173	-1,083	-1,760	-1,028	-0,251	0,002	-0,516	-1,296	-1,624	-1,380	-0,794	GO:0006629	lipid metabolism
11	Afu5g02850	conserved hypothetical protein	0,001	-0,336	-0,938	-1,437	-0,960	0,078	-0,017	-0,093	-0,838	-1,582	-1,470	-0,608	GO:0000004	unknown
11	Afu7g06080	ubiE/COQ5 methyltransferase, putative	-0,005	-0,846	-2,043	-1,364	-0,944	-0,624	-0,009	-0,360	-0,843	-0,693	-0,309	-0,346	GO:0000004	unknown
11	Afu7g01590	cystathionine gamma-synthase	0,024	-0,625	-2,041	-1,329	-0,835	-0,335	-0,002	-0,662	-1,168	-1,118	-0,913	-0,584	GO:0006519	amino acid
11	Afu2g01250	serine peptidase, family S28, putative	-0,001	-0,552	-1,773	-1,070	-1,231	-0,688	-0,002	-0,938	-0,925	-0,945	-0,785	-0,591	GO:0030163	protein catabolism
11	Afu2g05720	conserved hypothetical protein	-0,023	-0,411	-1,677	-1,613	-1,428	-1,041	-0,004	-0,539	-1,036	-1,126	-0,764	-0,460	GO:0000004	unknown
11	Afu2g01220	GTP cyclohydrolase II, putative	0,043	-0,252	-1,506	-1,287	-1,104	-0,785	-0,026	-0,476	-1,095	-0,423	-0,907	-0,661	GO:0006732	coenzyme metabolism
11	Afu4g13420	hypothetical protein	0,022	-0,673	-1,866	-0,719	-0,825	-0,784	-0,030	-0,445	-0,590	-1,049	-0,741	-0,687	GO:0000004	unknown
11	Afu2g16540	C2H2 finger domain protein, putative	0,002	-1,084	-1,268	-0,771	-1,177	-0,590	0,046	-0,652	-0,749	-1,045	-0,811	-0,532	GO:0045449	regulation of transcription
11	Afu8g05650	hypothetical protein	0,029	-0,432	-1,641	-1,272	-1,083	-0,506	0,004	-0,140	-0,986	-1,326	-0,966	-0,642	GO:0000004	unknown
11	Afu2g16520	phospholipase D (PLD), putative	0,015	-0,238	-1,483	-1,304	-1,143	-0,321	-0,005	-0,261	-0,999	-1,060	-0,871	-0,616	GO:0006629	lipid metabolism
11	Afu3g14590	copper amine oxidase	-0,128	-0,136	-1,286	-0,923	-1,012	-0,517	-0,033	-0,525	-0,934	-0,982	-0,946	-0,804	GO:0019725	homeostasis
11	Afu1g05050	cytosine deaminase, putative	0,022	-0,001	-1,378	-0,977	-1,019	-0,284	0,005	-0,286	-0,880	-1,125	-0,932	-0,703	GO:0006259	DNA metabolism
11	Afu4g00610	aryl-alcohol dehydrogenase, putative	0,049	-0,765	-1,369	-1,415	-1,141	-0,752	0,002	-0,282	-0,717	-1,194	-1,135	-0,712	GO:0000004	unknown
11	Afu6g12870	mitochondrial ABC-type iron exporter (Atm1), putative	-0,069	-0,386	-1,219	-1,290	-1,078	-0,764	-0,008	-0,108	-0,879	-0,920	-1,015	-0,719	GO:0006810	transport
11	Afu5g03750	WW domain protein	-0,038	-0,623	-1,472	-1,095	-1,006	-0,525	-0,050	-0,090	-0,555	-1,174	-0,776	-0,409	GO:0000004	unknown

11	Afu1g05320	disulfide isomerase, putative	0,057	-0,462	-1,430	-0,883	-0,589	-0,330	-0,015	-0,180	-0,832	-0,928	-0,850	-0,506	GO:0000004	unknown
11	Afu3g03450	cytochrome P450 pisatin demethylase, putative	0,037	0,038	-1,825	-1,336	-1,413	-0,990	0,022	-0,046	-0,769	-1,103	-0,683	-0,673	GO:0019748	secondary metabolism
11	Afu3g13620	conserved hypothetical protein	0,059	-0,008	-1,411	-1,128	-1,052	-0,769	-0,018	-0,147	-0,595	-0,949	-0,739	-0,989	GO:0000004	unknown
11	Afu2g11060	Acyl CoA binding protein family	0,018	-0,176	-1,386	-1,593	-0,950	-0,135	-0,005	0,134	-0,643	-1,204	-1,250	-0,274	GO:0000004	unknown
11	Afu6g06770	enolase	-0,017	-0,201	-1,350	-1,665	-1,095	-0,406	-0,005	-0,254	-0,261	-0,789	-1,027	-0,278	GO:0006091	energy pathways
12	Afu5g00210	hypothetical protein	0,008	-0,695	-1,339	-0,870	-1,054	1,272	-0,085	-0,147	-0,648	-0,371	-0,271	-0,243	GO:0000004	unknown
12	Afu7g05910	purine permease, putative	-0,020	0,735	-0,892	-0,288	-0,198	-0,200	-0,092	-0,352	-0,223	1,110	-0,003	-0,231	GO:0006810	transport
12	Afu7g08520	hypothetical protein	NaN	-0,692	-0,673	-0,274	-0,344	-0,191	-0,074	-0,339	-0,148	-0,014	-0,303	-0,121	GO:0000004	unknown
12	Afu1g11880	acetoacetyl-CoA synthase	0,011	-0,609	-0,418	-0,231	-0,592	-0,273	0,068	-0,461	-0,443	-0,304	-0,554	-0,268	GO:0006629	lipid metabolism
12	Afu6g12200	hypothetical protein	NaN	-0,326	-0,853	-0,486	-0,536	-0,155	0,030	-0,207	-0,550	-0,325	-0,388	-0,158	GO:0000004	unknown
12	Afu2g01850	phytanoyl-CoA dioxygenase family protein	0,019	-0,370	-0,916	-0,516	-0,370	-0,091	0,009	-0,229	-0,497	-0,376	-0,131	-0,165	GO:0000004	unknown
12	Afu1g13320	conserved hypothetical protein	-0,273	-0,380	-1,000	-0,316	-0,579	-0,234	-0,089	-0,488	-0,317	-0,627	-0,269	-0,361	GO:0000004	unknown
12	Afu4g06840	G-patch DNA repair protein (Drt111), putative	0,071	-0,132	-0,921	-0,198	-0,066	-0,099	0,021	-0,320	-0,523	-0,343	-0,171	-0,329	GO:0006259	DNA metabolism
12	Afu5g13210	alpha-1,2-mannosyltransferase (Alg2), putative	-0,117	-0,099	-0,929	-0,198	-0,189	-0,188	-0,020	-0,554	-0,537	-0,182	-0,105	-0,221	GO:0006464	protein modification
12	Afu6g07780	Copper fist DNA binding domain protein	0,003	-0,317	-1,263	-0,015	-0,208	-0,116	-0,004	-0,428	-0,314	-0,349	-0,072	-0,331	GO:0045449	regulation of transcription
12	Afu3g12810	transcriptional corepressor of histone genes (Hir3), putative	-0,127	-0,303	-0,950	0,077	-0,363	-0,218	0,011	-0,414	-0,548	-0,479	-0,096	-0,339	GO:0006259	DNA metabolism
12	Afu7g00220	plasma membrane hexose transporter, putative	NaN	-0,302	-0,825	-0,240	-0,101	-0,094	-0,051	-0,450	-0,789	-0,664	-0,407	-0,326	GO:0006810	transport
12	Afu5g12090	conserved hypothetical protein	-0,066	-0,025	-0,951	-0,157	-0,220	-0,166	-0,022	-0,496	-0,676	-0,544	-0,273	-0,431	GO:0000004	unknown
12	Afu2g17540	pigment biosynthesis oxidase Abr1/brown 1	NaN	-0,103	-1,077	-0,343	-0,139	-0,208	-0,015	-0,577	-0,855	-0,469	-0,297	-0,612	GO:0019748	secondary metabolism
12	Afu6g07590	Neurodap1-pending protein	0,079	-0,437	-1,088	-0,565	-0,116	-0,096	0,004	-0,582	-0,565	-0,511	-0,158	-0,387	GO:0000004	unknown
12	Afu5g06640	hypothetical protein	NaN	-0,317	-1,153	-0,369	-0,318	-0,227	0,012	-0,531	-0,662	-0,801	-0,191	-0,568	GO:0000004	unknown
12	Afu3g08480	sucrose transporter, putative	-0,020	-0,566	0,056	0,062	-0,237	-0,228	0,006	-0,607	-0,363	-0,400	-0,134	-0,352	GO:0006810	transport
12	Afu5g12550	N-acetylglucosaminyl-phosphatidylinositol deacetylase, putative	0,036	0,178	-1,140	-0,478	-0,317	-0,161	0,019	-0,005	-0,351	-0,272	-0,204	-0,142	GO:0006464	protein modification
12	Afu7g04750	hypothetical protein	NaN	0,406	-0,707	-0,360	-0,192	-0,006	0,029	0,096	-0,139	-0,148	-0,119	-0,088	GO:0000004	unknown
12	Afu2g17450	3-hydroxyanthranilate 3,4-dioxygenase	NaN	-0,634	-0,576	-0,156	-0,408	-0,012	0,073	0,292	-0,036	-0,050	-0,064	0,078	GO:0006732	coenzyme metabolism
12	Afu4g14000	tripeptidyl peptidase A	-0,001	-0,342	-0,968	-0,562	-0,402	-0,068	0,076	-0,015	-0,079	-0,036	0,046	-0,100	GO:0030163	protein catabolism
12	Afu5g10060	cytochrome b5 reductase, putative	NaN	-0,116	-0,452	-0,593	-0,519	-0,032	-0,086	-0,166	-0,205	-0,127	-0,035	-0,225	GO:0006091	energy pathways
12	Afu2g04570	BNR/Asp-box repeat domain protein	NaN	-0,126	-0,317	-0,556	-0,190	0,079	0,018	-0,054	0,031	-0,125	-0,071	0,030	GO:0000004	unknown
12	Afu6g02470	fumarate hydratase, putative	-0,005	-0,274	-1,302	-0,153	-0,014	0,184	-0,011	-0,307	-0,704	-0,308	-0,253	-0,078	GO:0006091	energy pathways
12	Afu6g11270	synaptobrevin-like protein	-0,092	-0,161	-1,160	-0,061	-0,049	0,090	0,013	-0,464	-0,455	-0,221	0,121	0,127	GO:0046965	intracellular transport
12	Afu3g09390	AMMECR1	-0,137	0,002	-0,992	-0,460	-0,042	0,489	0,076	-0,486	-0,706	-0,352	-0,100	0,126	GO:0000004	unknown
12	Afu8g00680	glycosyl transferase, group 2 family protein	0,011	-0,219	-0,695	-0,240	-0,048	0,275	0,026	-0,543	-0,582	-0,088	0,010	0,089	GO:0005975	carbohydrate metabolism
12	Afu2g17930	integral membrane protein	NaN	-0,700	-0,637	0,229	0,351	0,128	-0,030	-0,501	-0,492	-0,274	-0,241	0,080	GO:0000004	unknown
12	Afu1g15110	conserved hypothetical protein	0,066	-0,077	-0,604	0,147	NaN	0,051	0,059	-0,098	-0,375	-0,106	-0,042	-0,058	GO:0000004	unknown
12	Afu8g05380	hypothetical protein	-0,128	-0,193	-0,397	0,647	NaN	-0,081	0,015	-0,048	-0,177	-0,192	-0,074	0,012	GO:0000004	unknown
12	Afu1g00770	conserved hypothetical protein	NaN	-0,359	-0,142	-0,539	0,137	0,793	-0,007	-0,186	-0,353	-0,443	-0,278	0,528	GO:0000004	unknown
12	Afu1g17630	FAD/FMN-containing protein	0,057	-0,202	-0,322	-0,212	0,186	0,650	0,010	-0,500	-0,511	-0,672	-0,504	-0,163	GO:0000004	unknown
12	Afu5g05720	conserved hypothetical protein	NaN	0,031	-0,725	-0,439	-0,165	0,142	-0,079	-0,177	-0,902	-0,600	-0,523	-0,195	GO:0005975	carbohydrate metabolism
12	Afu5g09130	polysaccharide deacetylase family protein	NaN	-0,284	-0,805	-0,248	0,076	0,144	0,080	-0,270	-0,737	-0,460	-0,015	-0,137	GO:0005975	carbohydrate metabolism
12	Afu8g01290	hypothetical protein	NaN	-0,098	-0,832	-0,170	0,259	0,097	0,044	-0,220	-0,911	-0,510	-0,353	-0,339	GO:0000004	unknown
12	Afu5g08100	hypothetical protein	-0,083	0,187	-0,738	-0,216	0,098	0,523	0,039	-0,269	-0,774	-0,987	-0,404	-0,052	GO:0000004	unknown
12	Afu6g14360	cytochrome P450, putative	-0,067	-0,376	-1,203	0,166	0,370	0,289	0,018	-0,942	-0,190	0,463	0,243	0,163	GO:0000004	unknown
12	Afu1g16510	heme/steroid binding domain protein, putative	0,063	-0,070	-1,417	0,445	0,567	0,072	0,021	-0,608	-0,819	-0,754	0,037	-0,534	GO:0000004	unknown
13	Afu5g02320	hypothetical protein	0,013	-0,494	-1,569	-1,272	-1,505	-1,667	0,000	-0,396	-0,843	-1,444	-1,406	-1,400	GO:0000004	unknown
13	Afu1g06350	virulence related protein (Cap20), putative	-0,032	-0,154	-1,435	-0,979	-1,307	-1,825	-0,002	-0,337	-1,254	-0,456	-1,196	-1,551	GO:0000004	unknown
13	Afu2g03700	HMG-CoA reductase	-0,002	-1,056	-1,701	-1,236	-1,397	-1,252	0,010	-1,168	-0,979	-0,622	-1,054	-1,244	GO:0006629	lipid metabolism
13	Afu3g10660	hydroxymethylglutaryl-CoA synthase	-0,013	-0,948	-1,227	-0,592	-1,650	-1,069	-0,006	-1,086	-0,374	-0,310	-0,792	-0,490	GO:0006629	lipid metabolism
13	Afu1g13800	mfs-multidrug-resistance transporter	-0,037	-0,890	-0,853	-1,157	-1,463	-1,529	0,007	-1,223	-0,296	-0,880	-0,493	-0,914	GO:0006810	transport
13	Afu6g07470	conserved hypothetical protein	0,000	-1,011	-1,001	-1,398	-1,698	-1,110	0,001	-0,782	-0,539	-0,871	-0,810	-0,975	GO:0000004	unknown
13	Afu5g10920	DUF221 domain protein, putative	-0,011	-1,290	-0,855	-0,417	-0,799	-1,183	-0,019	-1,330	-1,181	-0,924	-1,153	-1,862	GO:0000004	unknown
13	Afu6g14090	CFEM domain protein, putative	-0,028	-0,271	-0,491	0,243	-0,680	-1,421	-0,075	-0,486	-1,025	-1,278	-1,085	-1,721	GO:0000004	unknown
13	Afu8g04050	NDT80_PhoG domain protein PcaG	0,019	-0,914	-0,284	-0,625	-1,018	-1,202	-0,003	-0,711	-0,617	0,155	-0,709	-1,097	GO:0045449	regulation of transcription
13	Afu1g00580	NDT80_PhoG domain protein PcaG	-0,041	-0,678	0,038	-0,500	-0,926	-1,113	0,051	-0,657	-0,467	0,158	-0,803	-1,226	duplicated cluster	duplicated cluster
13	Afu8g00970	hypothetical protein	0,064	-0,914	-0,289	-0,104	-0,786	-1,238	-0,036	-0,344	-0,092	-0,078	-0,293	-0,671	GO:0000004	unknown
13	Afu2g10850	C6 finger domain protein, putative	-0,052	-1,129	-0,125	-0,246	-0,637	-0,878	-0,069	-0,557	0,252	0,051	-0,570	-0,924	GO:0045449	regulation of transcription
13	Afu2g13770	C2H2 finger domain protein FlbC	-0,088	-0,909	0,452	-0,465	-0,808	-1,036	-0,037	-0,620	0,086	-0,339	-0,418	-0,767	GO:0045449	regulation of transcription
13	Afu1g09540	negative regulator of cdc42p	-0,036	-0,394	-0,595	-0,542	-0,840	-0,803	-0,083	-0,274	-0,598	-0,466	-0,316	-0,594	GO:0006464	protein modification
13	Afu5g01820	DUF221 domain protein, putative	-0,031	-0,618	-0,713	-0,425	-0,670	-0,805	-0,030	-0,334	-0,546	-0,360	-0,451	-0,495	GO:0000004	unknown
13	Afu2g07670	palmitoyltransferase (SidR), putative	0,007	-0,631	-0,908	-0,379	-0,697	-0,875	-0,022	-0,504	-0,667	-0,673	-0,586	-0,675	GO:0006464	protein modification
13	Afu2g00990	glycerophosphoryl diester phosphodiesterase family protein	NaN	-0,707	-0,699	-0,389	-0,633	-0,564	-0,020	-0,582	-0,444	-0,475	-0,512	-0,560	GO:0006629	lipid metabolism
13	Afu5g10910	hypothetical protein	-0,049	-0,306	-0,213	-0,340	-0,724	-0,713	-0,010	-0,545	-0,294	-0,284	-0,669	-0,992	GO:0000004	unknown
13	Afu5g12050	DNA ligase, putative	-0,214	0,135	-0,495	-0,176	-0,646	-0,582	-0,020	-0,236	-0,546	-0,357	-0,736	-0,953	GO:0006259	DNA metabolism
13	Afu7g05450	SUN domain protein (Uth1), putative	0,008	-0,021	-0,496	-0,614	-1,128	-1,088	0,013	-0,059	-0,107	-0,482	-0,649	-0,864	GO:0051301	cell division
13	Afu7g02460	hypothetical protein	-0,018	-0,085	-0,145	-0,797	-0,966	-1,151	-0,057	0,160	-0,239	-0,555	-0,636	-0,862	GO:0000004	unknown
13	Afu4g14190	hypothetical protein	-0,059	0,021	0,041	-0,584	-1,104	-1,093	0,029	-0,194	-0,172	-0,658	-0,625	-0,799	GO:0000004	unknown
13	Afu3g01500	integral membrane protein	0,048	0,212	-0,656	-0,436	-1,431	-1,382	0,033	-0,143	-0,522	-0,468	-0,401	-0,863	GO:0000004	unknown
13	Afu2g08380	diacylglycerol O-acyltransferase (DgaT), putative	-0,045	-0,229	-1,021	-0,735	-1,119	-1,156	-0,021	-0,174	-0,371	-0,585	-0,596	-0,694	GO:0006629	lipid metabolism
13	Afu6g14340	glucooligosaccharide oxidase, putative	-0,067	-0,356	-0,825	-0,945	-1,100	-1,221	0,061	-0,229	-0,597	-0,912	-0,478	-0,540	GO:0005975	carbohydrate metabolism
13	Afu4g14180	hypothetical protein	-0,048	-0,309	-0,669	-0,840	-1,245	-1,140	-0,002	-0,124	-0,621	-0,735	-0,582	-0,581	GO:0000004	unknown
13	Afu5g03540	pyridine nucleotide-disulphide oxidoreductase, class II, putative	0,022	-0,416	-0,552	-0,517	-1,212	-1,377	0,000	-0,081	-0,229	-0,558	-0,428	-0,805	GO:0006139	nucleobase, nucleoside, nucleotide and nucleic acid metabolism
13	Afu2g05360	C6 transcription factor, putative	0,007	-0,270	-0,672	-0,659	-1,247	-0,876	-0,042	-0,334	-0,001	-0,383	-0,483	-0,766	GO:0045449	regulation of transcription
13	Afu6g06740	endoplasmic reticulum calcium ATPase, putative	-0,017	-0,013	-1,097	-0,476	-1,007	-1,110	0,021	-0,529	-1,226	-0,507	-0,435	-1,097	GO:0006810	transport

13	Afu3g01850	porphyromonas-type peptidyl-arginine deiminase superfamily	0,034	-0,739	-1,199	-0,656	-0,978	-1,262	0,076	-0,452	-0,569	-0,463	-0,656	-1,070	GO:0000004	unknown
13	Afu1g05790	GPI anchored serine-rich protein	-0,029	-0,428	-0,803	-0,865	-1,185	-1,110	0,021	-0,363	-0,591	-0,794	-1,140	-0,903	GO:0000004	unknown
13	Afu5g02870	oxidoreductase, short-chain dehydrogenase/reductase family	0,025	-0,288	-0,971	-0,790	-0,959	-0,790	0,049	-0,217	-0,753	-0,745	-0,805	-0,906	GO:0006629	lipid metabolism
13	Afu3g11040	SNARE-dependent exocytosis protein (Sro7), putative	-0,055	-0,461	-1,037	-0,589	-0,943	-1,055	-0,030	-0,450	-0,732	-0,915	-0,866	-1,130	GO:0046982	intracellular transport
13	Afu1g12190	conserved hypothetical protein	0,048	-0,514	-1,260	-0,789	-0,860	-0,984	-0,039	-0,464	-0,814	-0,785	-0,878	-0,936	GO:0000004	unknown
13	Afu3g08620	csh3 protein	-0,001	-0,504	-1,001	-0,726	-1,101	-1,322	0,001	-0,060	-0,974	-0,884	-0,909	-1,011	GO:0000004	unknown
13	Afu7g00580	conserved hypothetical protein	0,014	-0,712	-0,797	-1,206	-1,275	-1,503	-0,012	-0,327	-0,879	-0,461	-0,876	-0,849	GO:0000004	unknown
13	Afu1g13980	conserved hypothetical protein	0,015	-0,943	-0,603	-0,357	-0,841	-0,945	-0,039	-0,713	-0,757	-0,800	-0,684	-1,033	GO:0000004	unknown
13	Afu1g09580	conserved hypothetical protein	0,011	-0,773	-0,595	-0,665	-0,829	-0,876	0,021	-0,575	-0,468	-1,025	-0,656	-0,737	GO:0000004	unknown
13	Afu1g13970	MFS transporter, putative	-0,117	-1,034	-0,789	-0,668	-1,031	-0,665	-0,038	-1,070	-0,816	-0,931	-1,023	-0,875	GO:0006810	transport
13	Afu6g00640	integral membrane protein	-0,002	-0,408	-0,500	-0,734	-0,882	-0,960	-0,020	-0,063	-0,485	-0,657	-0,820	-1,111	GO:0000004	unknown
13	Afu3g07890	endo alpha-1,4 polygalactosaminidase, putative	-0,052	-0,510	-0,423	-0,450	-0,577	-1,014	-0,023	-0,207	-0,967	-0,835	-0,665	-0,982	GO:0005975	carbohydrate metabolism
13	Afu1g03330	hypothetical protein	-0,027	-0,179	-0,593	-0,584	-0,952	-0,796	0,025	-0,633	-0,851	-0,714	-0,920	-1,104	GO:0000004	unknown
13	Afu1g09590	conserved hypothetical protein	0,022	0,053	-0,649	-0,565	-0,992	-1,186	0,079	-0,441	-0,518	-0,830	-0,743	-0,945	GO:0000004	unknown
13	Afu2g09070	DUF221 domain protein, putative	-0,010	-0,118	-0,463	-0,570	-0,900	-0,728	0,059	-0,568	-0,437	-1,110	-0,846	-0,889	GO:0000004	unknown
13	Afu3g00350	hypothetical protein	0,050	-0,349	-0,638	-0,864	-0,897	-0,740	-0,033	-0,057	-0,634	-1,234	-1,220	-1,053	GO:0000004	unknown
13	Afu4g00750	hypothetical protein	NaN	-0,463	-0,969	-0,401	-0,384	-0,281	-0,041	-0,009	-0,460	-0,280	-1,128	-1,530	GO:0000004	unknown
14	Afu4g09110	cytochrome c peroxidase, putative	NaN	3,532	4,519	2,964	2,061	2,117	0,025	3,920	4,650	5,246	4,380	4,654	GO:0006091	energy pathways
14	Afu6g09200	conserved hypothetical protein	NaN	3,964	4,589	2,388	1,813	1,379	-0,026	3,906	4,744	4,967	3,474	3,440	GO:0000004	unknown
14	Afu4g12530	cccA protein	NaN	2,770	4,146	2,939	1,511	0,665	0,015	5,069	3,535	4,339	3,454	3,178	GO:0006810	transport
14	Afu3g02270	mycelial catalase Cat1	NaN	3,045	4,222	2,349	0,737	0,500	0,330	3,735	NaN	3,812	2,871	3,123	GO:0006950	response to stress
14	Afu4g09140	ornithine aminotransferase	0,202	2,984	3,564	1,737	0,766	1,156	-0,069	2,978	2,912	2,981	1,816	1,748	GO:0006519	amino acid
14	Afu5g02020	aldehyde reductase (GliO), putative	0,075	3,298	3,869	1,627	0,387	0,313	-0,030	2,645	2,940	3,406	2,059	1,178	GO:0000004	unknown
14	Afu2g15960	nucleotide binding protein Nbp35, putative	NaN	3,751	2,438	1,056	1,008	1,296	-0,005	3,953	2,840	3,118	2,163	2,235	GO:0000004	unknown
14	Afu4g10690	Iron-sulfur cluster assembly accessory protein	0,015	2,940	2,838	1,958	1,488	1,454	0,013	3,919	3,037	3,368	2,656	2,783	GO:0006464	protein modification
14	Afu5g06270	5-aminolevulinic acid synthase	NaN	3,244	2,584	1,380	1,607	1,714	-0,043	3,789	2,425	2,550	2,758	2,697	GO:0006732	coenzyme metabolism
14	Afu5g13800	transcriptional regulator, putative	NaN	2,282	2,904	0,279	0,177	0,166	0,027	3,826	2,646	2,916	2,434	2,140	GO:0045449	regulation of transcription
14	Afu1g06610	NADH-quinone oxidoreductase, 23 kDa subunit, putative	0,053	2,042	2,605	1,602	1,255	1,226	-0,048	2,398	2,362	2,442	1,917	2,177	GO:0006091	energy pathways
14	Afu2g11260	3-isopropylmalate dehydratase, putative	-0,058	1,917	2,186	1,183	0,925	1,305	-0,026	2,995	2,216	2,549	2,045	2,642	GO:0006519	amino acid
14	Afu5g07750	ferrochelatase precursor	-0,140	2,768	2,344	1,044	1,212	1,161	-0,048	2,767	2,820	2,736	2,367	2,522	GO:0000065	unknown
14	Afu3g05870	IFRD domain protein	NaN	2,954	2,510	0,985	0,713	0,517	0,103	3,238	2,566	2,727	1,861	1,738	GO:0000004	unknown
14	Afu4g07780	mevalonate kinase	-0,041	2,608	2,239	1,001	0,763	0,705	-0,037	3,339	2,389	2,542	1,868	1,804	GO:0006629	lipid metabolism
14	Afu2g13110	cytochrome c	0,023	1,411	2,039	2,107	2,033	1,757	-0,065	1,492	2,155	3,131	2,918	2,670	GO:0006091	energy pathways
14	Afu1g15590	succinate dehydrogenase membrane anchor subunit, putative	-0,062	1,902	2,508	2,500	1,857	1,864	-0,044	1,269	1,741	2,424	2,780	2,343	GO:0006091	energy pathways
14	Afu5g10370	succinate dehydrogenase iron-sulphur protein	-0,077	2,141	2,420	2,243	1,740	1,607	-0,018	2,103	1,838	2,454	2,432	2,456	GO:0006091	energy pathways
14	Afu2g14210	mitochondrial dihydroxy acid dehydratase, putative	0,022	1,867	3,444	2,415	2,094	1,692	0,044	2,851	3,776	3,098	2,386	2,442	GO:0006519	amino acid
14	Afu5g06070	ABC multidrug transporter Mdr1	0,158	2,388	3,353	2,249	1,472	1,408	-0,007	2,656	4,192	3,887	2,730	1,920	GO:0006810	transport
14	Afu3g07810	succinate dehydrogenase, flavoprotein subunit	-0,011	1,946	3,332	2,432	1,992	1,908	-0,011	2,376	3,107	3,337	2,901	3,082	GO:0006091	energy pathways
14	Afu2g09130	NADH-ubiquinone dehydrogenase 24 kDa subunit, putative	-0,212	2,603	3,339	2,259	1,900	1,945	-0,002	2,560	3,007	2,994	2,854	2,918	GO:0006091	energy pathways
14	Afu5g12840	hydroxyacylglutathione hydrolase, putative	0,251	2,674	3,574	1,958	1,324	1,456	-0,023	2,488	2,884	3,093	2,612	2,696	GO:0005975	carbohydrate metabolism
14	Afu3g10110	electron transfer flavoprotein-ubiquinone oxidoreductase	0,197	2,310	2,629	2,001	1,761	1,631	-0,025	2,257	2,685	2,744	2,661	2,336	GO:0006732	coenzyme metabolism
14	Afu6g12930	mitochondrial aconitate hydratase, putative	-0,030	2,273	2,669	2,092	1,710	1,619	0,012	2,592	2,961	3,301	2,632	2,806	GO:0006091	energy pathways
14	Afu1g10310	RNase L inhibitor of the ABC superfamily, putative	-0,072	2,341	2,960	2,166	1,664	1,494	-0,044	2,988	3,144	2,837	2,681	2,262	GO:0016070	RNA metabolism
14	Afu1g17070	FYVE domain protein, putative	NaN	2,615	2,708	2,094	1,884	1,618	-0,145	3,053	2,646	2,882	2,312	2,227	GO:0000004	unknown
15	Afu6g07670	cytochrome c oxidase assembly protein cox15	-0,050	1,319	1,275	1,624	1,712	1,669	0,005	0,885	1,107	2,079	2,478	2,389	GO:0006091	energy pathways
15	Afu1g10770	neutral amino acid permease, putative	NaN	1,947	2,831	0,115	0,444	0,423	0,142	1,543	1,517	0,826	0,493	0,200	GO:0006810	transport
15	Afu5g09910	pnitroreductase family protein, putative	-0,246	1,719	1,963	0,337	0,567	0,882	-0,077	0,942	1,581	1,172	1,029	1,192	GO:0006629	lipid metabolism
15	Afu4g06890	14-alpha sterol demethylase Cyp51A	-0,029	2,362	2,312	-0,026	0,480	1,245	-0,020	1,931	1,906	1,206	1,049	1,416	GO:0006629	lipid metabolism
15	Afu2g15590	sulfite reductase [NADPH], putative	-0,012	1,325	2,765	0,378	1,447	1,116	0,008	1,308	2,166	1,025	1,615	1,201	GO:0045229	cell wall and envelope biogenesis
15	Afu4g08120	protease inhibitor (Tfs1), putative	NaN	0,975	1,718	0,811	0,121	-0,040	-0,061	1,153	1,541	2,450	1,240	0,738	GO:0000004	unknown
15	Afu4g12510	hypothetical protein	-0,054	1,764	1,674	0,955	0,686	0,404	0,070	1,613	1,103	1,994	1,078	0,521	GO:0000004	unknown
15	Afu1g04690	HIT finger domain protein, putative	-0,092	1,569	2,002	0,829	0,992	0,423	-0,070	1,890	1,247	1,323	1,112	1,028	GO:0000004	unknown
15	Afu1g03550	mitochondrial dihydroxy acid dehydratase, putative	0,148	1,472	1,906	0,810	0,665	0,554	0,007	1,849	1,984	1,342	1,023	1,093	GO:0006519	amino acid
15	Afu6g04700	imidazoleglycerol-phosphate dehydratase	0,037	1,745	2,095	0,399	0,526	0,677	0,069	1,728	1,707	1,574	1,042	1,346	GO:0006519	amino acid
15	Afu5g14870	protein kinase, putative	NaN	1,365	1,718	0,259	-0,176	0,016	-0,031	1,677	1,482	1,843	0,922	1,186	GO:0000004	unknown
15	Afu4g11960	Iron only hydrogenase large subunit, C-terminal domain containing protein	0,003	1,781	1,450	0,120	-0,021	0,188	0,074	1,824	1,215	1,724	0,758	0,920	GO:0006464	protein modification
15	Afu3g12630	cytochrome b561, putative	0,017	1,928	1,679	-0,122	-0,351	0,063	-0,012	2,117	1,741	2,010	1,204	1,348	GO:0006091	energy pathways
15	Afu7g07040	hypothetical protein	0,027	1,679	2,410	0,434	-0,111	-0,258	-0,290	2,096	1,409	1,698	1,263	1,045	GO:0000004	unknown
15	Afu6g00470	plasma membrane zinc ion transporter, putative	-0,105	2,136	1,372	0,993	0,119	-0,201	-0,034	1,921	1,408	2,296	1,465	1,450	GO:0006810	transport
15	Afu6g09190	metallopeptidase family M24, putative	-0,004	1,419	1,787	0,832	0,474	0,219	-0,027	2,326	1,806	2,395	1,924	2,049	GO:0030163	protein catabolism
15	Afu6g03070	extracellular dioxygenase, putative	NaN	1,758	2,312	0,759	0,296	0,147	0,054	1,957	1,682	2,470	1,655	1,116	GO:0000004	unknown
15	Afu6g07320	drug resistance transporter, EmrB/QacA family, putative	-0,322	2,124	2,525	1,031	0,991	0,690	0,008	2,080	1,402	2,365	1,609	1,554	GO:0006810	transport
15	Afu8g01710	antigenic thaumatin domain protein, putative	0,056	0,969	2,359	1,276	0,771	-0,128	-0,042	1,570	2,124	1,606	1,255	0,953	GO:0000004	unknown
15	Afu2g13720	RNA-polymerase I	-0,066	0,531	2,353	1,396	1,117	0,766	0,009	1,210	1,996	1,640	1,550	1,323	GO:0016070	RNA metabolism
15	Afu5g06780	carbamoyl-phosphate synthase, small subunit	-0,076	1,425	1,427	1,537	1,266	0,631	-0,019	1,890	1,563	2,051	1,892	1,744	GO:0006519	amino acid
15	Afu5g12000	hypothetical protein	NaN	2,012	1,789	1,055	1,169	0,891	0,151	1,704	1,744	1,664	1,355	1,196	GO:0000004	unknown
15	Afu5g04370	NADH-quinone oxidoreductase, subunit G, putative	0,012	1,488	2,072	1,408	0,961	0,949	0,022	1,338	1,544	1,953	1,675	1,499	GO:0006091	energy pathways
15	Afu5g11550	tRNA(m5U54)methyltransferase	-0,095	1,328	2,282	1,364	1,414	0,941	0,022	1,757	1,753	1,645	1,658	1,382	GO:0016070	RNA metabolism
15	Afu2g15780	Ca2+ dependent mitochondrial carrier protein, putative	0,089	1,760	2,085	1,465	1,353	0,784	0,002	1,807	1,738	1,815	1,591	1,280	GO:0006810	transport
15	Afu7g06630	cytochrome P450 monooxygenase, putative	NaN	0,887	2,071	1,739	1,497	1,136	0,239	1,070	1,125	1,967	1,549	1,434	GO:0000004	unknown
15	Afu2g10580	hypothetical protein	0,048	1,073	1,855	1,633	1,423	1,137	-0,034	1,133	1,377	2,076	1,827	1,902	GO:0000004	unknown
15	Afu2g13710	NADH-ubiquinone oxidoreductase 49 kDa subunit, putative	-0,018	0,987	2,232	1,657	1,317	1,264	-0,016	0,940	1,706	1,981	1,856	1,787	GO:0006091	energy pathways

15	Afu1g14200	mitochondrial processing peptidase beta subunit, putative	0,036	0,772	2,101	1,677	1,428	1,330	0,002	0,810	1,587	2,091	1,853	1,770	GO:0006464	protein modification
15	Afu5g10610	ubiquinol-cytochrome c reductase iron-sulfur subunit precursor	-0,081	1,383	2,122	2,009	1,587	1,366	0,000	1,013	1,418	2,053	2,082	1,967	GO:0006091	energy pathways
15	Afu1g15180	conserved hypothetical protein	NaN	2,608	2,454	0,353	0,217	0,196	-0,008	2,842	1,497	1,366	0,766	0,837	GO:0000004	unknown
15	Afu5g11290	D-amino acid oxidase	-0,046	2,521	1,332	-0,140	0,210	0,527	0,001	1,950	1,606	1,382	1,031	1,339	GO:0006519	amino acid
15	Afu8g05530	soluble fumarate reductase (Osm1), putative	-0,066	2,365	1,656	0,228	0,235	0,659	0,064	2,186	1,402	1,613	0,952	1,033	GO:0006091	energy pathways
15	Afu2g11680	HD family hydrolase, putative	NaN	2,502	1,682	0,335	0,679	0,721	0,124	2,520	0,987	1,749	1,257	1,252	GO:0000004	unknown
15	Afu5g03060	hypothetical protein	-0,146	2,482	1,321	0,614	0,681	0,668	-0,011	2,217	1,813	1,968	1,306	1,297	GO:0000004	unknown
15	Afu6g07200	conserved hypothetical protein	NaN	2,253	1,954	0,658	0,598	0,105	0,323	2,691	1,419	1,927	1,865	1,564	GO:0000004	unknown
15	Afu6g07100	diphthamide biosynthesis protein 2	0,091	2,639	1,571	0,603	0,906	0,852	0,015	2,928	1,695	1,883	1,779	1,628	GO:0006464	protein modification
15	Afu3g13140	7alpha-cephem-methoxylase P8 chain, putative	-0,504	2,010	2,080	0,435	0,702	0,916	-0,079	2,626	1,893	1,579	1,219	1,599	GO:0000004	unknown
15	Afu6g12920	C2H2 finger domain protein, putative	-0,094	1,758	1,926	0,858	1,001	0,350	-0,001	2,474	2,029	1,689	1,498	1,243	GO:0045449	regulation of transcription
15	Afu1g13510	C6 transcription factor (FacB), putative	0,020	2,353	1,855	1,252	0,870	0,590	0,047	2,559	1,939	2,179	1,385	1,346	GO:0045449	regulation of transcription
15	Afu3g13100	hypothetical protein	-0,069	2,335	2,132	1,037	0,772	0,721	-0,003	2,518	1,816	1,942	1,566	1,289	GO:0000004	unknown
15	Afu5g06090	type-III integral membrane protein (Ytp1), putative	-0,032	2,853	1,812	0,710	0,001	0,063	0,065	2,978	2,200	2,519	1,760	1,412	GO:0000004	unknown
15	Afu6g10880	acyl-CoA dehydrogenase, putative	NaN	2,790	2,960	0,416	0,302	0,380	-0,012	2,377	2,496	2,225	1,384	1,286	GO:0006629	lipid metabolism
15	Afu5g11260	siderophore transcription factor SreA	NaN	2,822	2,926	2,018	1,470	1,677	-0,050	0,942	1,065	1,145	1,253	1,627	GO:0045449	regulation of transcription
15	Afu5g06680	4-aminobutyrate aminotransferase	NaN	3,197	2,866	0,571	0,328	1,286	-0,021	1,603	1,047	1,174	1,290	1,554	GO:0000004	unknown
15	Afu8g01670	bifunctional catalase-peroxidase Cat2	NaN	2,493	3,317	1,117	0,657	0,755	-0,011	1,457	2,023	1,756	1,108	1,369	GO:0006950	response to stress
15	Afu6g09350	C6 finger domain protein, putative	0,177	1,763	2,187	1,384	1,780	1,513	0,142	2,566	2,129	1,887	1,794	1,928	GO:0045449	regulation of transcription
15	Afu3g00180	short chain dehydrogenase, putative	NaN	2,263	2,873	0,819	0,616	1,397	0,020	1,704	1,967	2,882	1,643	1,909	GO:0000004	unknown
15	Afu4g11050	NADH-ubiquinone oxidoreductase, subunit F, putative	-0,053	1,232	2,486	1,475	1,114	1,050	0,028	1,492	2,215	2,303	2,025	2,010	GO:0006091	energy pathways
15	Afu5g09680	succinate dehydrogenase cytochrome b560 subunit	0,040	1,622	2,227	2,050	1,100	1,265	0,101	1,456	2,074	2,036	1,920	1,843	GO:0000004	unknown
15	Afu5g08890	homoaconitase LysF	0,128	1,422	2,305	1,902	1,587	1,182	-0,022	1,482	2,290	2,342	2,213	1,584	GO:0006519	amino acid
15	Afu6g04290	phosphoethanolamine N-methyltransferase, putative	-0,051	1,970	2,242	1,489	0,995	1,063	0,110	1,946	2,260	2,280	2,047	1,997	GO:0006464	protein modification
15	Afu8g00790	hypothetical protein	NaN	1,922	3,425	1,635	1,246	1,328	0,057	1,802	2,279	2,336	1,604	2,139	GO:0000004	unknown
16	Afu1g01920	hypothetical protein	-0,141	2,662	0,514	-0,032	0,072	-0,118	0,047	1,974	0,216	0,815	0,405	0,257	GO:0000004	unknown
16	Afu4g10120	C6 finger domain protein, putative	0,015	1,062	-0,007	-0,066	0,488	0,866	0,049	1,228	0,255	0,933	0,732	0,792	GO:0045449	regulation of transcription
16	Afu4g01580	ankyrin repeat protein	NaN	0,517	1,318	0,059	-0,159	-0,140	-0,038	0,229	3,143	2,323	-0,175	-0,060	GO:0000004	unknown
16	Afu5g14890	hypothetical protein	-0,018	0,880	1,472	0,784	-0,084	-1,927	-0,035	1,227	1,709	1,868	1,078	0,089	GO:0000004	unknown
16	Afu7g06680	AAA family ATPase, putative	NaN	0,739	1,542	0,085	-0,337	-0,389	0,087	0,224	1,367	1,891	0,358	0,001	GO:0000004	unknown
16	Afu8g06760	integral membrane protein	-0,031	0,797	0,884	0,178	0,033	-0,611	0,038	0,253	1,579	0,735	0,312	0,147	GO:0000004	unknown
16	Afu3g00130	conserved hypothetical protein	NaN	1,083	1,083	0,506	0,364	-0,051	0,018	0,135	1,863	0,970	0,432	0,369	GO:0000004	unknown
16	Afu6g10120	oxidoreductase, zinc-binding	-0,031	1,274	1,545	0,398	0,199	0,533	-0,003	1,175	1,774	1,316	0,208	0,577	GO:0000004	unknown
16	Afu1g16880	ABC multidrug transporter, putative	NaN	1,115	2,100	0,398	0,016	0,077	0,142	1,104	1,322	1,422	0,595	0,538	GO:0006810	transport
16	Afu1g06810	aconitate hydratase, mitochondrial	NaN	0,339	1,716	0,526	0,068	0,307	0,099	0,944	1,542	1,044	0,716	0,331	GO:0000004	unknown
16	Afu5g10760	alpha-1,2-mannosyltransferase (Kre2), putative	-0,094	0,986	1,223	0,608	0,385	0,151	0,075	1,162	1,110	0,980	0,643	0,320	GO:0006464	protein modification
16	Afu1g10300	hypothetical protein	0,050	0,731	1,239	0,742	0,377	0,027	-0,053	1,228	1,003	0,946	0,546	0,561	GO:0000004	unknown
16	Afu4g06610	hypothetical protein	NaN	1,097	1,494	0,569	0,345	0,298	-0,005	1,258	1,301	1,223	0,838	0,492	GO:0000004	unknown
16	Afu8g02500	glutathione S-transferase	NaN	1,211	0,363	-0,040	-0,319	-0,272	0,042	1,695	1,235	1,424	0,811	0,980	GO:0000004	unknown
16	Afu5g07760	tetracycline-efflux transporter, putative	0,011	2,554	1,008	0,642	0,051	0,021	0,041	1,332	0,461	0,956	0,239	0,112	GO:0000004	unknown
16	Afu1g15560	hypothetical protein	0,000	2,196	1,156	0,542	0,167	-0,361	0,106	1,296	0,981	1,131	0,722	0,199	GO:0000004	unknown
16	Afu2g04060	NADH:flavin oxidoreductase/NADH oxidase family protein	NaN	1,549	1,637	-0,036	-0,270	-0,583	-0,111	1,650	0,922	1,235	0,312	0,131	GO:0000004	unknown
16	Afu2g07480	C6 finger domain protein, putative	NaN	1,540	1,202	0,194	-0,215	-0,283	-0,048	1,625	1,093	1,172	0,460	0,399	GO:0045449	regulation of transcription
16	Afu5g13810	transulfuration enzyme family protein, putative	NaN	2,190	1,872	0,122	-0,067	-0,120	0,058	1,283	1,019	0,801	0,370	0,544	GO:0000004	unknown
16	Afu3g12390	hypothetical protein	NaN	2,168	1,501	0,272	-0,037	-0,082	0,019	1,743	1,224	1,085	0,364	0,328	GO:0000004	unknown
16	Afu4g06190	fungal specific transcription factor, putative	NaN	1,780	0,564	0,332	0,206	0,311	0,022	1,603	0,721	1,104	0,551	0,645	GO:0045449	regulation of transcription
16	Afu3g09810	conserved hypothetical protein	-0,018	1,551	1,362	0,153	0,072	0,077	0,082	1,821	0,998	1,365	0,641	0,867	GO:0047012	intracellular transport
16	Afu6g01980	haemolysin-III family protein	NaN	1,582	1,281	0,313	0,173	-0,018	-0,081	1,570	0,889	1,253	0,818	0,822	GO:0000004	unknown
16	Afu3g06740	C6 transcription factor (Gal4), putative	0,332	1,382	1,139	0,430	0,322	0,155	-0,064	1,485	1,014	1,013	0,712	0,650	GO:0045449	regulation of transcription
16	Afu6g04230	RING finger protein	-0,074	1,870	0,991	0,010	0,199	0,139	-0,032	1,309	1,028	0,628	0,462	0,372	GO:0006412	protein biosynthesis
16	Afu5g09830	cation efflux family protein, putative	-0,076	2,017	1,212	-0,060	0,069	0,115	-0,003	1,651	0,922	0,995	0,396	0,420	GO:0006810	transport
16	Afu3g06730	MFS sugar transporter, putative	0,044	2,254	1,177	0,193	0,020	0,132	0,008	1,611	0,886	0,571	0,485	0,416	GO:0006810	transport
16	Afu3g05590	DNA helicase, putative	0,001	1,962	1,031	0,328	0,382	0,108	-0,052	1,821	0,738	0,830	0,776	0,799	GO:0006259	DNA metabolism
16	Afu1g10080	C2H2 transcription factor, putative	NaN	2,022	1,074	0,288	0,319	0,269	-0,013	2,019	0,613	0,757	0,742	0,630	GO:0045449	regulation of transcription
16	Afu5g12020	C6 transcription factor (NirA), putative	NaN	2,070	1,536	0,407	0,458	0,452	0,069	1,737	0,957	0,853	0,591	0,572	GO:0045449	regulation of transcription
16	Afu7g01740	sugar transporter, putative	-0,166	1,286	1,518	-0,007	0,124	0,007	-0,009	1,225	0,734	0,580	0,473	0,389	GO:0006810	transport
16	Afu1g05260	DNA-directed DNA polymerase theta, putative	-0,313	1,322	1,105	0,521	-0,035	0,056	0,053	1,216	0,985	1,101	0,462	0,384	GO:0006259	DNA metabolism
16	Afu4g10680	conserved hypothetical protein	0,028	1,489	1,125	0,357	-0,035	0,217	0,027	1,369	0,673	0,687	0,481	0,366	GO:0000004	unknown
16	Afu3g03610	hypothetical protein	-0,094	1,393	0,979	0,450	0,282	0,372	0,073	1,125	0,658	0,842	0,736	0,584	GO:0000004	unknown
16	Afu7g01490	MFS peptide transporter, putative	-0,053	1,721	1,162	0,638	-0,017	-0,749	-0,005	0,988	0,764	1,783	0,614	0,398	GO:0006810	transport
16	Afu6g00480	hypothetical protein	NaN	1,588	1,481	0,384	0,059	-0,392	0,173	1,600	1,335	1,497	0,966	0,999	GO:0000004	unknown
16	Afu7g00480	ABC multidrug transporter, putative	-0,049	0,592	1,917	0,331	-0,353	-0,935	0,010	-0,023	1,067	0,646	0,059	-0,370	GO:0006810	transport
16	Afu3g03260	hypothetical protein	-0,026	1,429	1,698	-0,249	-0,927	-1,231	0,052	0,986	0,558	0,439	-0,373	-0,399	GO:0000004	unknown
16	Afu6g02170	metalloreductase, putative	-0,017	0,646	1,024	0,064	-0,642	-0,970	-0,033	0,993	0,779	0,708	0,133	-0,151	GO:0006810	transport
16	Afu3g08960	epoxide hydrolase	0,080	1,304	1,687	0,323	-0,335	-0,480	-0,028	0,770	0,971	0,655	-0,015	-0,179	GO:0006629	lipid metabolism
16	Afu5g09480	ABC transporter, putative	-0,085	1,924	2,257	1,052	0,482	-0,363	0,037	0,680	0,727	0,387	0,114	-0,187	GO:0006810	transport
16	Afu4g08240	alcohol dehydrogenase, zinc-containing	0,003	1,924	1,243	0,368	-0,457	0,191	-0,025	0,497	0,547	0,467	0,203	0,365	GO:0006091	energy pathways
16	Afu4g08170	succinate-semialdehyde dehydrogenase, putative	-0,012	2,604	1,814	-0,037	-0,177	0,487	-0,025	0,354	0,280	0,184	0,392	0,506	GO:0006519	amino acid
16	Afu1g15350	homoserine acetyltransferase family protein	NaN	1,530	1,309	-0,394	-0,139	-0,381	-0,036	0,434	0,390	1,078	0,123	NaN	GO:0006519	amino acid
16	Afu3g02360	FAD dependent oxidoreductase superfamily	NaN	0,939	0,811	0,029	-0,410	-0,184	0,063	1,066	0,396	0,306	0,001	0,102	GO:0000004	unknown
16	Afu2g09940	hypothetical protein	-0,012	0,848	1,087	0,146	-0,098	-0,309	-0,016	1,344	0,659	0,312	0,223	-0,007	GO:0000004	unknown
16	Afu2g03410	hypothetical protein	0,000	0,520	0,971	-0,072	-0,270	-0,199	-0,116	0,598	0,569	0,250	0,044	-0,147	GO:0000004	unknown

16	Afu8g04470	MFS transporter, putative	0,060	0,984	0,966	0,112	-0,088	-0,104	0,066	0,304	0,569	0,191	-0,095	-0,039	GO:0006810	transport
16	Afu1g10590	GPI anchored protein, putative	NaN	0,776	1,158	0,305	0,101	-0,128	-0,009	0,321	0,194	0,388	0,182	-0,021	GO:0000004	unknown
16	Afu2g14200	protein kinase, putative	NaN	0,656	0,353	0,291	-0,212	-0,110	-0,121	0,579	0,498	0,444	0,103	0,037	GO:0006464	protein modification
16	Afu2g04150	C6 transcription factor, putative	-0,076	0,856	0,720	-0,090	-0,098	-0,003	-0,036	0,672	0,408	0,479	0,235	0,202	GO:0045449	regulation of transcription
16	Afu8g05040	dihydrodipicolinate synthetase family protein	NaN	0,880	0,553	0,203	0,033	-0,094	0,068	0,842	0,275	0,407	0,140	0,311	GO:0000004	unknown
16	Afu7g07130	hypothetical protein	NaN	1,069	0,777	0,210	-0,368	-0,019	-0,072	0,626	0,169	0,510	0,170	-0,058	GO:0000004	unknown
16	Afu2g03820	carboxyphosphonoenolpyruvate phosphonomutase, putative	0,021	1,067	0,546	0,284	-0,009	-0,154	-0,034	0,354	0,130	0,701	0,247	0,068	GO:0006091	energy pathways
16	Afu5g02620	cytochrome P450, putative	NaN	0,923	0,682	0,279	0,108	0,067	0,029	0,463	0,196	0,403	0,175	0,068	GO:0000004	unknown
16	Afu4g06580	hypothetical protein	NaN	0,517	0,942	0,001	-0,176	-0,100	-0,304	0,571	0,162	0,500	0,260	0,394	GO:0000004	unknown
16	Afu4g10450	hypothetical protein	NaN	0,773	0,690	0,301	0,086	0,015	-0,436	0,603	0,371	0,262	0,286	0,227	GO:0000004	unknown
16	Afu7g01760	hypothetical protein	NaN	1,007	1,135	-0,268	0,288	-0,171	0,046	0,648	0,634	0,219	0,028	-0,151	GO:0000004	unknown
16	Afu7g01750	DUF218 domain protein	0,096	1,285	1,204	0,176	0,114	-0,289	0,066	0,892	0,550	0,240	0,264	-0,182	GO:0000004	unknown
16	Afu3g13940	DUF1212 domain membrane protein	0,055	1,429	1,734	0,158	0,011	-0,208	-0,027	0,376	0,468	0,257	0,083	-0,154	GO:0000084	unknown
16	Afu6g07600	oxidoreductase, FAD-binding, putative	NaN	1,365	1,337	0,155	0,423	0,318	0,076	0,637	0,200	0,321	0,075	0,158	GO:0000004	unknown
16	Afu2g00180	neutral amino acid permease	-0,023	1,480	0,374	-0,014	-0,187	0,084	-0,029	1,510	1,115	0,401	0,396	0,468	GO:0006810	transport
16	Afu5g07740	DSB repair complex subunit Ku70, putative	0,123	1,280	0,956	0,213	0,168	0,262	-0,002	1,256	1,284	0,601	0,084	0,156	GO:0006259	DNA metabolism
16	Afu5g15010	arsenite permease (ArsB), putative	-0,116	0,983	0,665	0,366	-0,441	-0,302	0,069	0,744	0,947	0,908	0,523	0,323	GO:0006810	transport
16	Afu2g10540	hypothetical protein	NaN	0,948	0,587	0,314	-0,338	0,153	0,012	0,502	0,622	0,851	0,341	0,223	GO:0000004	unknown
16	Afu4g06300	hypothetical protein	0,092	1,200	0,593	0,125	0,081	0,061	0,048	0,801	0,490	0,873	0,230	0,120	GO:0000004	unknown
16	Afu2g08800	amino acid permease (Dip5), putative	0,009	1,217	0,586	0,036	0,095	-0,059	0,068	0,898	0,345	1,229	0,252	0,292	GO:0006810	transport
16	Afu1g07750	FUN19 protein	0,043	1,203	0,307	-0,127	-0,195	0,022	-0,039	0,693	0,693	0,429	0,314	0,349	GO:0000004	unknown
16	Afu5g07110	integral membrane protein	-0,016	1,243	0,698	0,063	0,229	0,145	0,095	0,761	0,658	0,529	0,336	0,317	GO:0000004	unknown
16	Afu6g04690	hypothetical protein	NaN	1,237	0,623	0,105	0,105	0,181	-0,042	0,733	0,544	0,628	0,487	0,339	GO:0000004	unknown
16	Afu6g08750	delta-1-pyrroline-5-carboxylate dehydrogenase	NaN	1,344	0,659	0,106	0,093	0,363	0,140	0,690	0,235	0,471	0,321	0,644	GO:0006519	amino acid
16	Afu1g16830	C6 transcription factor, putative	NaN	1,177	0,731	0,196	0,176	0,039	0,052	1,273	0,651	1,008	0,517	0,425	GO:0045449	regulation of transcription
16	Afu6g07090	maleylacetate reductase, putative	NaN	1,088	0,415	0,053	0,312	0,127	-0,019	1,239	0,696	0,878	0,604	0,750	GO:0006091	energy pathways
16	Afu1g15040	hypothetical protein	NaN	0,879	0,480	0,170	0,214	0,203	0,051	0,964	0,682	0,848	0,819	0,444	GO:0000004	unknown
16	Afu5g09800	hypothetical protein	NaN	0,864	0,663	0,039	-0,122	0,607	NaN	0,870	0,459	0,899	0,638	0,514	GO:0000004	unknown
16	Afu5g08590	endo-1,4-beta-xylanase, putative	NaN	0,852	0,749	0,034	0,440	0,251	0,098	1,086	0,698	0,608	0,019	0,418	GO:0005975	carbohydrate metabolism
16	Afu5g08600	homoserine O-acetyltransferase	-0,106	0,858	0,816	-0,184	0,745	0,567	-0,004	0,906	0,389	0,283	0,427	0,390	GO:0006519	amino acid
16	Afu5g14230	C6 transcription factor, putative	-0,084	0,781	0,842	0,013	0,387	0,400	-0,038	0,346	0,652	0,703	0,367	0,718	GO:0045449	regulation of transcription
16	Afu6g11450	C6 transcription factor, putative	NaN	0,585	0,787	0,108	0,384	0,179	0,261	0,775	0,367	0,558	0,332	0,404	GO:0045449	regulation of transcription
16	Afu7g06640	hypothetical protein	-0,221	0,769	0,854	0,194	0,238	0,177	-0,072	0,465	0,242	0,324	0,227	0,355	GO:0000004	unknown
16	Afu5g07570	pyruvate carboxylase, putative	NaN	0,496	0,824	0,346	0,208	0,237	0,051	0,535	0,463	0,381	0,185	0,380	GO:0006732	coenzyme metabolism
16	Afu3g09100	hypothetical protein	-0,063	0,847	0,606	0,139	0,308	0,140	-0,003	0,408	0,534	0,548	0,326	0,221	GO:0000004	unknown
16	Afu4g00920	MFS pantothenate transporter, putative	NaN	0,752	0,759	0,459	0,328	0,108	0,056	0,391	0,338	0,449	0,414	0,249	GO:0006810	transport
16	Afu3g08040	polymerase (RNA) II (DNA directed) polypeptide D	-0,040	0,678	0,598	0,673	0,310	0,097	0,079	0,646	0,296	0,580	0,290	-0,006	GO:0016070	RNA metabolism
16	Afu5g10620	CBF/NF-Y family transcription factor, putative	0,032	1,010	0,531	0,369	0,584	0,134	-0,064	0,774	0,425	0,479	0,464	0,218	GO:0016070	RNA metabolism
16	Afu2g03500	sugar transporter, putative	NaN	0,315	0,765	0,728	0,271	-0,270	0,069	0,531	0,529	0,553	0,424	0,286	GO:0006810	transport
16	Afu3g00790	methylaspartate ammonia-lyase, putative	-0,057	0,463	0,684	0,647	0,100	0,087	-0,041	0,592	0,505	0,757	0,693	0,683	GO:0006091	energy pathways
16	Afu5g11080	MSF multidrug transporter, putative	-0,049	0,597	1,283	0,296	0,165	-0,085	0,014	0,432	1,153	0,572	0,201	0,055	GO:0006810	transport
16	Afu4g05890	conserved hypothetical protein	-0,078	0,217	1,212	0,322	0,196	-0,027	0,068	0,450	0,859	0,757	0,252	0,356	GO:0000004	unknown
16	Afu1g12220	hypothetical protein	NaN	0,374	1,149	0,177	-0,192	-0,235	0,018	0,693	0,754	1,011	0,505	0,521	GO:0000004	unknown
16	Afu8g05760	dehydrogenase	NaN	0,395	1,470	0,495	0,264	-0,017	0,013	0,899	0,973	0,940	0,731	0,713	GO:0000004	unknown
16	Afu6g11510	flavodoxin and radical SAM domain protein	-0,299	0,942	1,309	0,453	0,174	0,468	0,273	0,694	1,115	0,798	0,561	0,163	GO:0006091	energy pathways
16	Afu6g08390	conserved hypothetical protein	-0,133	0,771	1,550	0,468	0,276	0,196	0,048	0,715	0,785	0,228	0,735	0,578	GO:0000004	unknown
16	Afu1g02820	Y20 protein	NaN	0,889	1,286	0,271	0,193	0,287	-0,071	0,627	0,565	0,415	0,158	0,469	GO:0006091	energy pathways
16	Afu3g03600	carboxyl esterase A	-0,029	0,892	1,200	0,115	0,068	0,059	0,046	0,732	0,864	0,823	0,285	0,393	GO:0006629	lipid metabolism
16	Afu8g04860	GPI anchored glycoprotein, putative	NaN	0,672	1,478	0,428	0,122	0,112	0,114	0,552	0,862	0,741	0,325	0,554	GO:0000004	unknown
16	Afu1g04710	PP-loop ATPase superfamily protein, putative	-0,036	0,652	1,086	0,271	0,299	0,414	-0,036	0,717	0,516	0,595	0,531	0,443	GO:0000004	unknown
16	Afu1g13190	GRISEA protein, putative	-0,022	0,705	1,096	0,479	0,385	0,255	0,080	0,999	0,584	0,887	0,540	0,633	GO:0045449	regulation of transcription
16	Afu3g05860	exonuclease	0,197	0,698	1,050	0,559	0,450	0,176	0,063	0,607	0,773	0,675	0,494	0,332	GO:0016070	RNA metabolism
16	Afu1g06380	RNA polymerase III subunit CII	0,138	0,653	1,348	0,524	0,719	0,244	-0,090	0,693	0,710	0,417	0,486	0,239	GO:0016070	RNA metabolism
16	Afu4g07080	dual specificity phosphatase, putative	NaN	0,701	1,304	0,440	0,607	0,527	-0,055	0,451	0,696	0,525	0,513	0,342	GO:0000004	unknown
16	Afu4g09510	conserved hypothetical protein	NaN	1,770	1,075	0,203	0,245	0,620	-0,018	0,520	0,340	0,711	0,584	0,789	GO:0000004	unknown
16	Afu5g12150	PH domain protein	0,111	1,456	0,813	0,357	0,459	0,547	0,004	0,762	0,247	0,332	0,335	0,322	GO:0000004	unknown
16	Afu5g05600	forkhead transcription factor (Sep1), putative	0,055	1,380	0,571	0,370	0,514	0,591	-0,045	0,810	0,535	0,545	0,292	0,447	GO:0045449	regulation of transcription
16	Afu6g11160	isopentenyl-diphosphate delta-isomerase	0,027	1,783	0,654	0,742	0,707	0,500	-0,013	0,896	0,516	0,555	0,539	0,316	GO:0019748	secondary metabolism
16	Afu2g06120	hypothetical protein	-0,112	1,241	1,065	0,476	0,501	0,473	-0,013	0,955	0,559	0,310	0,514	0,568	GO:0000004	unknown
16	Afu1g09130	conserved hypothetical protein	-0,014	1,084	1,168	0,725	0,744	0,430	0,017	0,918	0,421	0,540	0,373	0,492	GO:0000004	unknown
16	Afu6g12750	rhomboid family protein, putative	-0,095	1,235	1,084	0,778	0,495	0,277	-0,249	0,898	0,901	0,873	0,601	0,226	GO:0006464	protein modification
16	Afu2g15790	C-type cyclin (Ume3), putative	NaN	1,066	1,047	0,683	0,594	0,252	-0,036	0,903	0,578	0,788	0,703	0,476	GO:0007049	cell cycle
16	Afu3g10120	TATA-box binding protein	-0,015	1,267	1,203	0,752	0,610	0,170	-0,020	0,951	0,698	0,610	0,569	0,222	GO:0016070	RNA metabolism
16	Afu5g11990	alpha-1,3-mannosyltransferase (Alg3), putative	-0,053	1,318	1,245	0,793	0,677	0,457	0,055	0,796	0,665	0,591	0,679	0,330	GO:0006464	protein modification
17	Afu5g14210	glucose-repressible gene protein-related protein	0,031	0,979	0,617	0,728	0,968	1,209	-0,024	0,297	-0,494	-0,960	0,037	0,724	GO:0000004	unknown
17	Afu5g08290	aldo-keto reductase, putative	NaN	-0,053	0,093	0,230	-0,054	0,566	0,028	-0,013	0,374	-0,153	-0,523	0,063	GO:0006629	lipid metabolism
17	Afu2g03240	hypothetical protein	NaN	-0,080	0,026	0,229	0,258	0,456	0,029	-0,037	-0,110	-0,208	0,169	0,158	GO:0000004	unknown
17	Afu4g08130	mitochondrial hypoxia responsive domain protein	0,038	-0,029	-0,164	0,208	0,236	0,612	-0,075	-0,069	-0,227	0,134	0,171	0,086	GO:0000004	unknown
17	Afu1g01960	conserved hypothetical protein	NaN	0,048	-0,005	0,174	0,096	0,681	-0,158	-0,043	-0,026	-0,053	0,063	0,207	GO:0000004	unknown
17	Afu2g08900	voltage-gated chloride channel (ClcA), putative	-0,022	-0,020	-0,004	0,265	0,016	0,521	-0,090	-0,178	0,054	0,242	0,096	0,220	GO:0019725	homeostasis
17	Afu6g13500	conserved hypothetical protein	NaN	-0,125	0,372	0,507	0,798	0,513	-0,069	-0,053	-0,001	-0,190	0,114	0,194	GO:0000004	unknown
17	Afu1g11610	3-dehydroquinate dehydratase, type II	-0,089	-0,144	0,026	0,581	0,645	0,670	0,087	0,018	-0,033	0,082	0,320	0,374	GO:0005975	carbohydrate metabolism

17	Afu5g03310	hypothetical protein	-0,084	-0,044	0,239	0,377	0,888	0,618	-0,003	0,230	-0,088	0,087	0,459	0,346	GO:0000004	unknown
17	Afu1g15280	polysaccharide deacetylase family protein	NaN	0,030	0,401	0,111	0,350	0,691	0,018	-0,292	-0,171	-0,522	-0,104	-0,303	GO:0005975	carbohydrate metabolism
17	Afu4g06070	translation initiation regulator (Gcn20), putative	-0,005	0,384	0,381	0,392	0,430	0,447	-0,030	-0,266	0,124	-0,162	0,115	-0,062	GO:0006412	protein biosynthesis
17	Afu2g02820	Yippee zinc-binding protein, putative	0,048	0,826	-0,585	-0,116	0,302	0,762	-0,011	0,359	-0,103	-0,037	0,249	0,779	GO:0000004	unknown
17	Afu3g10530	protein serine/threonine kinase (Ran1), putative	-0,066	0,938	-0,209	-0,017	-0,189	0,512	-0,031	-0,273	-0,155	0,035	0,080	0,446	GO:0006464	protein modification
17	Afu6g10040	fructosyl amine: oxygen oxidoreductase	NaN	0,361	-0,221	-0,150	0,315	0,611	0,058	0,122	-0,319	-0,110	0,189	0,252	GO:0006519	amino acid
17	Afu3g06040	glutaryl-CoA dehydrogenase, putative	-0,041	0,644	-0,041	-0,097	0,348	0,870	-0,026	0,182	-0,245	-0,284	-0,060	0,220	GO:0000004	unknown
17	Afu8g04110	DUF895 domain membrane protein	-0,016	0,685	-0,060	0,058	0,288	0,352	0,133	-0,111	-0,285	-0,073	-0,031	0,075	GO:0000004	unknown
17	Afu1g00440	DUF895 domain membrane protein	-0,048	0,841	-0,092	0,397	0,389	0,506	0,146	-0,080	-0,274	-0,096	0,010	0,005	duplicated cluster	duplicated cluster
17	Afu4g08250	Raffinose synthase or seed imbibition protein Sip1, putative	NaN	0,279	0,130	0,226	0,378	0,428	0,035	0,087	0,005	-0,016	0,193	0,420	GO:0005975	carbohydrate metabolism
17	Afu6g00540	conserved hypothetical protein	0,783	0,490	0,315	0,163	0,355	0,630	0,136	0,146	0,189	-0,279	0,162	0,525	GO:0000004	unknown
17	Afu1g11000	C6 transcription factor, putative	NaN	0,399	0,353	0,171	0,314	0,168	0,019	0,008	-0,306	0,008	0,153	0,221	GO:0045449	regulation of transcription
17	Afu2g16960	CAF1 family ribonuclease, putative	NaN	0,563	0,364	0,359	0,622	0,330	-0,049	0,035	-0,127	0,110	0,411	0,246	GO:0016070	RNA metabolism
17	Afu2g05390	Domain found in Dishevelled, Egl-10, and Pleckstrin domain protein	0,095	0,392	0,001	0,453	0,556	0,736	0,018	0,182	-0,135	-0,040	0,113	0,298	GO:0000004	unknown
17	Afu4g12490	guanine nucleotide exchange factor VPS9, putative	0,183	0,648	0,427	0,236	0,300	0,654	0,092	0,321	-0,055	-0,137	0,218	0,654	GO:0046913	intracellular transport
17	Afu1g10800	thioesterase family protein	-0,059	0,863	0,115	0,101	0,302	0,436	0,026	0,380	-0,089	0,077	0,225	0,200	GO:0006629	lipid metabolism
17	Afu5g03460	thymidylate kinase	-0,051	0,766	0,438	0,557	0,384	0,542	0,079	0,286	-0,150	0,066	0,145	0,147	GO:0006259	DNA metabolism
17	Afu6g06830	hypothetical protein	-0,121	1,005	0,293	0,313	0,704	0,771	-0,014	0,371	0,009	0,398	0,128	0,247	GO:0016070	RNA metabolism
17	Afu1g10220	hypothetical protein	-0,076	0,535	-0,119	0,150	0,153	0,217	0,054	0,073	0,195	0,241	0,329	0,357	GO:0000004	unknown
17	Afu1g16970	ADP-ribosylation factor family protein	0,003	0,599	0,138	0,183	0,118	0,150	-0,051	0,156	0,143	0,169	0,193	0,101	GO:0046911	intracellular transport
17	Afu3g05790	hypothetical protein	NaN	0,815	0,333	0,334	0,100	0,308	-0,054	0,494	0,249	0,323	0,181	0,364	GO:0000004	unknown
17	Afu6g14330	5-oxo-L-prolinase, putative	0,032	0,069	0,154	0,601	0,261	0,134	0,030	-0,425	-0,272	0,764	0,429	-0,124	GO:0000004	unknown
17	Afu2g11220	proline permease, putative	NaN	-0,105	0,788	0,500	0,174	0,602	0,351	0,464	-0,177	0,631	0,246	0,562	GO:0006810	transport
17	Afu5g12770	metallo-beta-lactamase family protein	NaN	0,716	0,975	0,471	0,628	0,660	-0,071	0,171	0,252	0,143	0,417	0,208	GO:0019748	secondary metabolism
17	Afu8g05950	hypothetical protein	-0,083	0,394	0,902	0,258	0,792	0,704	0,007	0,477	0,467	0,236	0,186	0,120	GO:0000004	unknown
17	Afu1g15490	MFS multidrug transporter, putative	NaN	0,585	0,944	0,373	0,225	0,231	-0,168	-0,037	-0,240	0,026	0,147	0,187	GO:0006810	transport
17	Afu4g07090	C2H2 finger domain protein, putative	NaN	0,474	0,981	0,234	0,267	0,371	-0,027	0,195	0,150	-0,003	0,165	-0,156	GO:0045449	regulation of transcription
17	Afu6g02430	hypothetical protein	NaN	0,028	0,949	0,393	0,369	0,328	-0,044	0,316	0,300	0,266	0,218	0,142	GO:0000004	unknown
17	Afu5g02260	ABC multidrug transporter, putative	NaN	0,189	0,789	0,202	0,223	0,441	-0,072	0,020	0,348	0,380	0,331	0,228	GO:0006810	transport
17	Afu5g01060	hypothetical protein	-0,089	0,161	0,771	0,357	0,243	0,046	-0,063	0,039	0,393	0,434	0,359	0,171	GO:0000004	unknown
17	Afu2g03050	forkhead domain protein	0,038	0,173	0,916	0,077	0,200	0,180	0,024	0,487	0,436	0,269	0,369	0,410	GO:0045449	regulation of transcription
17	Afu5g08200	hypothetical protein	0,000	-0,004	0,180	0,790	0,464	0,365	0,005	-0,119	0,255	0,240	0,553	0,377	GO:0000004	unknown
17	Afu2g14460	oxidoreductase, short-chain dehydrogenase/reductase family	NaN	0,256	0,089	0,327	0,616	0,356	-0,024	0,174	0,066	0,374	0,381	0,370	GO:0006629	lipid metabolism
17	Afu1g02950	RNA recognition motif. (a.k.a. RRM, RBD, or RNP domain) protein	NaN	0,295	0,091	0,562	0,463	0,338	0,074	0,189	0,138	0,294	0,300	0,290	GO:0016070	RNA metabolism
17	Afu4g14740	serine/threonine protein kinase (Ark1), putative	NaN	0,154	-0,052	0,872	0,251	0,342	NaN	0,222	0,018	0,258	0,250	0,150	GO:0000004	unknown
17	Afu3g07610	hypothetical protein	NaN	0,192	0,130	0,963	0,287	-0,111	0,106	0,214	0,097	0,075	0,121	-0,031	GO:0000004	unknown
17	Afu5g04100	PQ loop repeat protein	0,256	0,207	0,452	0,611	0,440	0,188	-0,148	-0,023	0,120	0,163	0,273	-0,094	GO:0000004	unknown
17	Afu2g04970	Kelch repeats protein, putative	0,187	0,053	0,406	0,466	0,637	0,231	-0,025	-0,147	0,352	-0,051	0,251	-0,111	GO:0000004	unknown
17	Afu2g11920	hypothetical protein	-0,012	0,019	0,689	0,639	0,339	0,240	0,023	0,062	0,323	0,026	0,200	0,052	GO:0000004	unknown
17	Afu8g00720	amino acid transporter, putative	NaN	0,169	0,637	0,896	0,330	-0,091	-0,002	-0,049	0,028	0,111	0,283	-0,071	GO:0019748	secondary metabolism
17	Afu1g02350	MFS transporter, putative	NaN	0,661	0,615	0,617	0,516	0,131	-0,063	0,081	0,120	0,291	0,292	0,202	GO:0006810	transport
17	Afu2g06020	mitochondrial respiratory complex I chaperone (Cia84), putative	-0,090	0,314	0,830	0,606	0,772	0,446	-0,095	0,058	0,389	0,271	0,352	0,174	GO:0006091	energy pathways
17	Afu4g12030	hypothetical protein	NaN	0,059	0,658	0,524	0,650	0,241	0,140	0,053	0,202	0,515	0,351	0,144	GO:0000004	unknown
17	Afu1g11720	conserved hypothetical protein	NaN	0,348	0,285	0,296	0,745	-0,053	0,089	0,117	0,258	0,474	0,527	0,142	GO:0000004	unknown
17	Afu3g11210	YagE family protein	-0,157	0,145	0,393	0,526	0,740	0,220	-0,059	0,321	0,336	0,637	0,585	0,442	GO:0000004	unknown
17	Afu3g11230	hypothetical protein	-0,085	0,192	0,523	0,524	0,610	0,327	-0,076	0,322	0,489	0,828	0,704	0,503	GO:0000004	unknown
17	Afu5g03330	conserved hypothetical protein	0,067	0,774	0,246	-0,011	0,079	0,379	0,025	0,306	0,182	0,586	0,683	1,003	GO:0000004	unknown
17	Afu8g02190	hypothetical protein	NaN	0,857	0,560	0,408	0,433	0,381	-0,248	0,315	0,247	0,424	0,533	1,059	GO:0000004	unknown
17	Afu2g12500	major facilitator superfamily	-0,114	1,039	0,401	0,524	0,933	0,527	-0,014	-0,054	-0,150	-0,007	0,477	0,236	GO:0006810	transport
17	Afu1g17550	HET domain protein	NaN	1,022	0,453	0,866	1,016	1,017	0,059	0,016	-0,158	0,381	0,703	0,511	GO:0000004	unknown
17	Afu1g02960	hypothetical protein	NaN	0,765	0,428	0,899	1,127	0,603	-0,012	0,229	0,059	0,314	0,549	0,423	GO:0000004	unknown
17	Afu7g06270	cyanamide hydratase	NaN	1,194	0,835	0,322	0,741	0,812	0,094	0,091	0,047	0,189	0,208	0,721	GO:0006950	response to stress
17	Afu7g05350	delta-9 fatty acid desaturase; stearoyl-CoA desaturase	0,040	1,516	-0,227	0,416	0,585	0,739	-0,017	0,330	-0,112	0,162	0,265	0,421	GO:0006629	lipid metabolism
17	Afu5g13410	SNF7 family protein	-0,047	1,162	0,353	0,510	0,676	0,864	-0,021	0,737	0,314	0,344	0,548	0,629	GO:0046918	intracellular transport
17	Afu8g05580	coenzyme A transferase PsecoA	NaN	0,886	0,273	0,675	0,957	1,334	0,034	0,048	-0,150	0,100	0,699	1,161	GO:0006732	coenzyme metabolism
17	Afu5g00600	NPP1 domain protein, putative	NaN	0,870	0,567	0,504	0,710	0,944	-0,014	0,345	0,053	0,486	0,749	1,207	GO:0000004	unknown
17	Afu4g09870	hypothetical protein	0,042	0,508	0,066	0,812	0,963	1,124	0,002	-0,164	-0,662	-0,211	0,381	0,240	GO:0000004	unknown
17	Afu3g12760	hypothetical protein	NaN	0,334	-0,142	0,778	0,836	1,030	0,006	-0,018	-0,336	-0,313	0,453	0,783	GO:0000004	unknown
17	Afu3g09260	hypothetical protein	-0,049	0,100	-0,340	0,784	0,648	0,508	0,059	-0,083	-0,053	0,108	0,524	0,629	GO:0000004	unknown
17	Afu1g05840	hypothetical protein	-0,027	-0,415	-0,091	0,718	0,671	0,712	0,065	-0,206	-0,105	0,366	0,455	0,577	GO:0000004	unknown
17	Afu4g10130	alpha-amylase (Amy1), putative	NaN	-0,122	0,060	0,285	0,722	0,637	0,097	-0,195	-0,034	0,192	0,539	0,769	GO:0005975	carbohydrate metabolism
17	Afu4g09910	conserved hypothetical protein	NaN	-0,229	0,033	0,376	0,711	0,711	0,069	-0,163	0,133	0,446	0,655	0,944	GO:0000004	unknown
17	Afu3g02610	MFS transporter, putative	NaN	-0,248	-0,275	0,232	0,941	0,964	0,159	NaN	-0,106	-0,094	0,366	0,545	GO:0006810	transport
17	Afu2g05400	sugar hydrolase, putative	NaN	0,043	-0,078	0,380	0,985	0,853	0,033	-0,204	-0,228	0,013	0,487	0,809	GO:0045229	cell wall and envelope biogenesis
17	Afu3g11060	THUMP domain protein	NaN	-0,115	-0,107	0,640	0,895	0,842	-0,085	-0,056	0,108	0,024	0,463	0,711	GO:0000004	unknown
17	Afu4g13770	glycosyl hydrolase, putative	NaN	0,094	0,018	0,396	0,741	1,146	-0,029	-0,187	0,163	0,139	0,293	0,577	GO:0045229	cell wall and envelope biogenesis
17	Afu3g14570	histidine acid phosphatase, putative	NaN	-0,132	-0,031	0,202	0,941	1,015	-0,035	-0,138	0,128	0,246	0,487	0,760	GO:0006810	transport
17	Afu1g07210	conserved hypothetical protein	NaN	0,119	-0,163	0,287	0,564	1,172	-0,059	-0,258	-0,232	0,010	0,301	0,893	GO:0000004	unknown
17	Afu7g01290	conserved hypothetical protein	0,056	0,055	-0,047	0,534	0,708	1,126	-0,054	-0,427	-0,059	0,216	0,649	0,962	GO:0000004	unknown
17	Afu7g06280	conserved hypothetical protein	NaN	0,911	-0,186	0,194	0,636	0,929	0,011	-0,103	-0,151	-0,029	0,303	0,525	GO:0000004	unknown
17	Afu7g01700	hypothetical protein	0,092	0,554	-0,400	0,513	0,745	0,816	0,021	0,070	0,161	0,069	0,431	0,598	GO:0000004	unknown
17	Afu6g02190	transcriptional activator (PtaC), putative	0,072	0,542	-0,100	0,629	0,907	1,066	-0,041	0,234	-0,134	0,198	0,394	0,554	GO:0045449	regulation of transcription

17	Afu6g00560	hypothetical protein	0,081	0,323	0,057	0,326	0,880	1,251	0,059	0,393	-0,083	0,139	0,183	0,671	GO:0000004	unknown
17	Afu6g14410	amidase family protein	NaN	0,527	0,200	0,360	0,876	1,262	-0,129	-0,142	0,087	0,121	0,514	0,663	GO:0006091	energy pathways
17	Afu6g00400	conserved hypothetical protein	NaN	0,171	0,120	0,475	0,698	0,857	-0,036	-0,041	0,035	0,498	0,848	1,030	GO:0000004	unknown
17	Afu7g05880	hypothetical protein	0,010	0,240	0,077	0,450	0,993	1,038	0,005	-0,217	0,290	0,348	0,722	0,799	GO:0000004	unknown
17	Afu7g05890	hypothetical protein	-0,030	0,248	0,126	0,314	0,950	0,921	0,123	-0,111	0,384	0,435	0,625	0,706	GO:0000004	unknown
17	Afu2g05990	conserved hypothetical protein	-0,022	0,276	0,118	0,291	0,669	0,797	-0,021	-0,021	0,243	0,043	0,380	0,461	GO:0000004	unknown
17	Afu2g04370	glutamate carboxypeptidase	0,004	0,164	0,367	0,223	0,765	0,812	0,053	0,188	0,219	0,182	0,420	0,621		
17	Afu5g13850	small oligopeptide transporter, OPT family	NaN	0,170	0,383	0,332	0,633	0,744	0,000	-0,169	0,036	0,401	0,337	0,640	GO:0006810	transport
17	Afu4g00350	hypothetical protein	0,022	0,195	0,341	0,568	0,665	0,554	-0,018	0,040	0,146	0,418	0,526	0,582	GO:0000004	unknown
17	Afu2g04360	WD repeat protein	0,142	0,314	0,506	0,397	1,056	1,089	-0,024	-0,077	0,338	0,120	0,350	0,446	GO:0000004	unknown
17	Afu6g10720	alpha-ketoglutarate-dependent taurine dioxygenase	NaN	0,398	0,821	0,459	0,788	0,909	-0,030	0,167	0,118	0,121	0,332	0,605	GO:0000004	unknown
17	Afu3g08460	60s ribosomal protein l37b	0,039	-0,083	0,247	1,122	1,383	0,695	-0,027	-0,064	0,074	0,324	0,650	0,373	GO:0006412	protein biosynthesis
17	Afu7g01270	carbamoyl-phosphate synthase, large subunit	0,087	0,001	0,102	0,898	1,062	0,940	-0,007	-0,539	0,118	0,370	0,712	0,628	GO:0006519	amino acid
17	Afu2g01590	non-classical export protein (Nce2), putative	NaN	-0,154	0,226	1,012	0,927	0,544	-0,012	-0,366	0,100	0,494	0,833	0,876	GO:0006810	transport
17	Afu4g07270	serine carboxypeptidase, putative	NaN	0,127	0,409	0,898	0,689	0,843	0,047	-0,166	0,158	0,557	0,368	0,569	GO:0007165	signal transduction
17	Afu7g06780	hypothetical protein	-0,002	0,007	0,644	1,100	0,935	0,781	-0,037	0,083	0,082	0,429	0,392	0,469	GO:0000004	unknown
17	Afu5g08220	hypothetical protein	-0,035	1,001	0,038	0,974	1,012	0,768	0,012	-0,329	0,610	0,297	0,590	0,334	GO:0000004	unknown
17	Afu8g05970	TRI7, putative	0,103	0,580	0,556	1,004	0,828	0,902	-0,101	-0,082	0,526	0,759	0,829	0,698	GO:0019748	secondary metabolism
18	Afu1g13210	uracil permease	NaN	0,432	0,279	0,949	0,291	0,030	-0,004	-0,044	0,062	3,582	0,689	-0,062	GO:0006139	nucleobase, nucleoside, nucleotide and nucleic acid metabolism
18	Afu2g03860	plasma membrane low affinity zinc ion transporter, putative	0,025	1,592	1,637	1,680	1,970	2,566	0,041	1,001	0,730	0,831	1,311	2,356	GO:0006810	transport
18	Afu5g01380	conserved hypothetical protein	NaN	0,464	0,446	0,114	0,770	0,908	-0,008	0,333	0,644	0,968	2,063	3,306	GO:0000004	unknown
18	Afu4g14380	conserved hypothetical protein	-0,060	-0,424	1,374	0,606	-0,102	1,343	-0,002	0,334	1,331	2,217	2,116	1,552	GO:0000004	unknown
18	Afu3g01900	conserved hypothetical protein	-0,030	1,290	0,261	1,492	1,557	1,438	-0,006	0,245	0,477	0,252	0,659	0,651	GO:0000004	unknown
18	Afu5g01390	hypothetical protein	NaN	0,384	0,423	0,627	0,609	0,879	0,027	0,227	0,325	0,625	0,921	1,714	GO:0000004	unknown
18	Afu2g12510	hypothetical protein	NaN	0,933	0,728	0,189	0,977	1,516	-0,057	0,573	0,402	0,546	1,123	1,068	GO:0000004	unknown
18	Afu4g06790	ubiquinol-cytochrome c reductase complex 14 kDa protein	-0,007	0,419	0,562	0,717	0,968	0,846	0,022	0,453	0,506	0,700	0,803	0,866	GO:0006091	energy pathways
18	Afu3g06170	mating type protein MAT1-2-1	NaN	0,785	0,892	1,177	1,268	1,026	-0,030	0,135	0,324	0,468	0,805	0,598	GO:0007165	signal transduction
18	Afu5g07490	steroid monooxygenase [imported]	NaN	0,859	0,573	1,085	1,087	1,153	-0,006	0,350	0,244	0,727	0,997	0,848	GO:0006629	lipid metabolism
18	Afu5g07480	hypothetical protein	-0,044	0,907	0,308	1,116	1,185	1,166	0,023	0,331	0,034	0,800	1,147	1,057	GO:0000004	unknown
18	Afu5g13840	NUDIX domain, putative	-0,080	0,854	0,728	1,029	1,456	1,124	0,043	0,001	0,896	1,116	0,921	1,068	GO:0000004	unknown
18	Afu2g17000	PT repeat family protein	-0,037	0,444	0,900	1,203	1,482	1,618	-0,163	0,067	0,341	0,792	1,190	1,268	GO:0000004	unknown
18	Afu2g04190	conserved hypothetical protein	-0,331	1,634	0,502	0,813	0,699	1,213	-0,028	0,556	0,334	0,622	0,669	1,181	GO:0000004	unknown
18	Afu5g12080	hypothetical protein	-0,265	1,328	0,634	0,590	0,616	0,975	-0,007	0,998	0,503	0,516	0,563	0,776	GO:0000004	unknown
18	Afu5g13560	FHA domain protein	-0,116	1,555	0,066	0,975	1,023	1,188	-0,033	1,180	0,475	0,900	0,946	1,199	GO:0000004	unknown
18	Afu7g03770	hypothetical protein	-0,099	1,756	0,294	0,470	0,922	1,324	0,032	1,243	0,300	0,378	0,478	1,264	GO:0000004	unknown
18	Afu5g07780	squalene monooxygenase Erg1	-0,034	2,072	0,156	0,619	0,876	1,023	0,012	1,638	0,369	0,695	0,907	0,947	GO:0006629	lipid metabolism
18	Afu4g11400	cell surface receptor/MFS transporter (FLVCR), putative	0,105	0,668	0,468	1,182	0,916	0,934	-0,028	0,436	0,669	1,099	1,634	1,450	GO:0045229	cell wall and envelope biogenesis
18	Afu4g11270	hypothetical protein	0,001	0,429	0,978	0,654	0,785	1,226	0,013	0,242	0,988	1,612	1,441	1,665	GO:0000004	unknown
18	Afu5g04210	ubiquinol-cytochrome C reductase complex core protein 2, putative	0,011	0,997	1,193	1,657	1,441	1,494	-0,025	0,217	0,828	1,370	1,829	1,616	GO:0006091	energy pathways
18	Afu4g12520	hypothetical protein	-0,027	0,435	1,041	0,790	0,481	0,085	0,035	0,208	1,056	1,928	0,827	0,169	GO:0000004	unknown
18	Afu4g14640	low affinity iron transporter, putative	-0,123	0,687	1,047	1,372	0,728	0,123	0,054	0,465	0,883	0,803	1,310	0,225	GO:0006810	transport
18	Afu2g02290	hypothetical protein	0,135	0,801	0,691	0,729	0,615	0,403	0,057	0,820	0,547	0,917	1,012	0,814	GO:0006412	protein biosynthesis
18	Afu3g04120	conserved hypothetical protein	0,021	0,372	1,012	0,820	0,496	0,241	0,022	0,665	0,700	0,979	0,888	0,388	GO:0006259	DNA metabolism
18	Afu6g12490	BolA domain protein	-0,049	0,743	1,239	0,468	0,487	0,389	-0,016	0,630	0,677	0,927	0,778	0,736	GO:0000004	unknown
18	Afu1g12110	NADH:ubiquinone oxidoreductase (NADH dehydrogenase),14 kDa	-0,083	0,764	1,118	0,960	0,845	0,611	0,006	0,580	0,878	1,272	0,918	0,736	GO:0006091	energy pathways
18	Afu5g02750	cytochrome c oxidase subunit Va, putative	-0,001	0,946	1,146	0,998	0,922	1,135	-0,058	0,439	0,586	0,863	0,837	0,860	GO:0006091	energy pathways
18	Afu6g02960	NADH-ubiquinone oxidoreductase 105 kDa subunit	-0,020	0,961	0,878	1,025	1,029	0,904	0,012	0,583	0,634	1,146	0,942	0,838	GO:0006091	energy pathways
18	Afu1g11930	protein kinase, putative	0,020	1,062	0,976	0,991	0,977	0,848	-0,011	0,635	0,772	0,958	0,916	0,949	GO:0006464	protein modification
18	Afu6g04620	NADH-ubiquinone oxidoreductase B14 subunit, putative	0,126	0,913	1,051	1,050	0,972	0,923	-0,039	0,746	0,784	0,961	0,999	1,104	GO:0006091	energy pathways
18	Afu5g10560	cytochrome c oxidase subunit V	-0,007	0,912	1,156	0,776	0,767	0,982	-0,021	0,660	0,810	1,042	0,922	1,215	GO:0006091	energy pathways
18	Afu1g07380	NADH-dependent glutamate synthase (GLT1), putative	-0,051	0,248	1,798	1,347	0,628	0,667	0,058	0,124	1,522	2,277	1,302	1,162	GO:0006519	amino acid
18	Afu2g11810	conserved hypothetical protein	0,012	0,031	1,713	1,034	0,947	0,342	0,220	0,441	1,659	1,087	1,383	0,965	GO:0016070	RNA metabolism
18	Afu4g08110	translation elongation factor G1, putative	-0,031	0,442	1,558	1,401	1,274	0,758	-0,091	0,729	1,353	1,617	1,673	1,128	GO:0006412	protein biosynthesis
18	Afu8g01770	hypothetical protein	0,022	0,659	1,618	1,247	0,722	0,740	-0,002	0,650	1,387	1,828	1,023	0,941	GO:0000004	unknown
18	Afu3g04110	rRNA processing protein (Bystin), putative	-0,008	0,596	1,654	1,258	0,828	0,322	0,006	0,905	1,654	1,400	1,430	0,708	GO:0016070	RNA metabolism
18	Afu2g05880	ammonium transporter MEAA	0,043	0,800	1,954	1,647	1,046	0,549	0,000	0,717	0,929	2,075	0,936	0,358	GO:0006810	transport
18	Afu1g10610	hypothetical protein	-0,042	1,570	1,316	0,085	0,851	0,553	-0,030	1,731	1,187	0,628	1,117	1,264	GO:0000004	unknown
18	Afu6g13900	conserved hypothetical protein	NaN	0,973	1,309	0,288	0,289	0,303	-0,030	1,058	0,993	1,211	0,880	0,977	GO:0000004	unknown
18	Afu6g04430	ARD/ARD family protein, putative	0,095	0,777	1,497	0,226	0,501	0,772	0,042	1,082	1,339	0,871	0,467	0,997	GO:0000004	unknown
18	Afu4g09130	mannosyltransferase, putative	NaN	1,017	0,999	0,925	0,633	0,483	0,081	1,235	0,881	1,195	0,846	0,745	GO:0006464	protein modification
18	Afu2g08340	hypothetical protein	NaN	1,353	1,336	0,643	0,859	0,708	-0,002	1,194	0,640	0,887	1,101	0,850	GO:0000004	unknown
18	Afu2g01650	conserved hypothetical protein	-0,036	1,647	0,950	0,599	0,625	0,438	0,031	1,241	0,708	0,979	0,720	0,856	GO:0000004	unknown
18	Afu5g02690	C6 transcription factor, putative	0,101	1,753	0,900	0,581	0,441	0,366	0,007	1,343	0,846	1,129	0,926	0,925	GO:0045449	regulation of transcription
18	Afu4g06280	hypothetical protein	NaN	1,317	1,491	0,778	0,296	0,382	0,243	1,637	0,853	1,186	0,791	0,877	GO:0000004	unknown
18	Afu4g13010	conserved hypothetical protein	0,099	1,487	1,376	0,753	0,570	0,546	0,005	1,359	1,129	1,069	0,739	0,601	GO:0000004	unknown
18	Afu4g08490	acyl-CoA dehydrogenase, putative	-0,142	1,718	1,413	0,607	0,522	0,579	0,021	1,295	0,988	1,128	0,857	0,855	GO:0006629	lipid metabolism
18	Afu1g03780	C6 finger domain protein, putative	-0,106	1,091	1,714	0,754	0,599	0,257	0,067	0,902	0,990	0,897	0,864	0,536	GO:0045449	regulation of transcription
18	Afu2g07720	cytochrome b5, putative	0,037	0,865	1,564	0,853	0,513	0,507	-0,041	0,727	1,252	1,324	0,908	0,849	GO:0006091	energy pathways
18	Afu3g10150	hypothetical protein	0,034	1,250	1,631	1,096	0,929	0,538	-0,072	1,140	0,928	1,218	0,980	0,708	GO:0000004	unknown
18	Afu6g04630	conserved hypothetical protein	0,070	1,042	1,845	0,991	1,118	0,532	0,011	1,366	1,136	1,088	1,035	0,639	GO:0000004	unknown
18	Afu1g07170	Na/H antiporter, putative	-0,032	1,292	1,439	0,744	0,585	0,481	-0,022	1,530	1,245	1,198	0,927	0,797	GO:0019725	homeostasis
18	Afu1g04590	ada3 protein, putative	-0,004	1,350	1,480	0,960	0,752	0,440	-0,009	1,430	1,198	1,324	1,059	0,919	GO:0006259	DNA metabolism

18	Afu6g08430	conserved hypothetical protein	0,045	1,216	1,674	0,747	0,738	0,579	0,055	1,390	1,430	1,290	0,852	0,813	GO:0000004	unknown
18	Afu4g09880	conserved hypothetical protein	0,034	1,384	1,718	1,007	0,899	0,663	-0,010	1,224	1,239	1,305	0,827	0,842	GO:0000004	unknown
18	Afu4g06900	asparagine synthetase (glutamine-hydrolyzing) 2	0,052	1,041	1,391	0,521	1,121	1,036	-0,083	0,993	2,036	1,123	0,842	0,984	GO:0006519	amino acid
18	Afu1g12290	possible NADH-ubiquinone oxidoreductase	0,003	1,038	1,225	1,068	0,939	0,848	0,010	1,287	1,046	1,419	1,327	1,466	GO:0006091	energy pathways
18	Afu5g12040	hypothetical protein	-0,116	1,224	0,992	0,581	0,538	0,757	-0,019	1,196	1,239	1,401	1,075	1,157	GO:0000004	unknown
18	Afu1g04680	NifU-related protein	-0,043	1,564	1,045	0,646	0,828	0,919	-0,044	1,460	1,380	1,090	1,233	1,123	GO:0019725	homeostasis
18	Afu4g05860	mitochondrial NADH-ubiquinone oxidoreductase 20 kD subunit, putative	0,056	1,450	1,572	1,250	1,107	1,264	-0,068	0,812	1,208	1,391	1,647	1,367	GO:0006091	energy pathways
18	Afu6g02020	hypothetical protein	-0,047	1,888	1,088	1,161	0,987	1,087	-0,005	0,936	1,379	1,287	1,172	1,211	GO:0007049	cell cycle
18	Afu2g04710	cytochrome b5, putative	-0,065	1,697	1,494	1,183	0,782	0,817	-0,020	1,225	1,282	1,296	1,281	0,923	GO:0006091	energy pathways
19	Afu3g06530	ATP sulphurylase	-0,004	-0,604	1,232	-1,670	0,544	0,511	0,012	0,041	0,402	-0,780	0,131	0,190	GO:0006519	amino acid
19	Afu6g11090	hypothetical protein	NaN	-0,189	0,864	-0,891	1,181	0,838	0,064	0,381	0,272	-1,029	-0,160	0,108	GO:0000004	unknown
19	Afu6g11100	basic amino acid transporter, putative	0,016	-0,243	1,022	-0,982	0,806	0,607	0,080	0,233	0,013	-1,115	-0,128	-0,131	GO:0006810	transport
19	Afu1g05020	sulfate transporter, putative	-0,031	0,390	1,295	-0,721	0,785	1,053	-0,019	0,524	-0,006	-1,062	0,103	-0,196	GO:0006810	transport
19	Afu3g02080	allantoate permease	-0,189	0,499	2,084	-0,258	0,281	0,374	0,004	0,304	-0,117	-0,392	-0,173	-0,054	GO:0006810	transport
19	Afu5g04250	homocysteine synthase, putative	0,035	0,481	2,086	-0,466	0,654	0,892	0,003	0,551	0,874	-0,744	-0,577	0,031	GO:0006519	amino acid
19	Afu3g06540	3'-phosphoadenosine-5'-phosphosulfate reductase	0,045	1,406	1,312	-1,059	1,106	0,651	-0,030	2,105	0,565	-0,533	0,321	0,334	GO:0006519	amino acid
20	Afu7g04290	amino acid permease (Gap1), putative	-0,089	2,080	0,144	-0,655	-0,971	-0,937	-0,053	0,812	-1,373	1,042	-0,611	-1,360	GO:0006810	transport
20	Afu6g12180	conserved hypothetical protein	0,036	1,409	0,109	-0,266	-1,065	-0,243	-0,023	0,953	-0,307	-1,937	-1,554	-0,914	GO:0000004	unknown
20	Afu2g00320	sterol delta 5,6-desaturase, putative	-0,005	1,642	0,506	-1,407	-0,518	-0,107	0,008	0,795	-0,603	-2,000	-0,762	-0,776	GO:0006629	lipid metabolism
20	Afu3g00820	putative exported protein	0,148	1,986	-0,531	-0,708	-0,276	0,300	0,023	1,581	-0,393	-0,944	-0,182	0,224	GO:0000004	unknown
20	Afu3g00810	cholestenol delta-isomerase, putative	-0,053	1,948	-0,228	-1,144	-0,309	0,751	-0,003	1,426	-0,722	-1,085	-0,277	0,426	GO:0000004	unknown
20	Afu1g13350	transporter, putative	0,103	1,038	-0,727	-0,194	-0,470	-0,777	0,005	0,641	-0,474	-0,791	-0,450	-0,365	GO:0006810	transport
20	Afu2g11030	hypothetical protein	-0,303	1,092	-0,852	-0,784	-0,706	-0,384	-0,043	0,441	-0,266	-0,476	-0,437	NaN	GO:0000004	unknown
20	Afu5g03290	hypothetical protein	-0,147	1,581	-1,043	-0,757	-0,095	0,121	0,013	0,834	-0,500	-0,828	-0,327	-0,171	GO:0000004	unknown
20	Afu7g04740	hypothetical protein	0,040	1,242	-1,065	-0,495	-0,244	-0,042	-0,048	0,416	-0,292	-0,212	-0,060	-0,112	GO:0000004	unknown
20	Afu2g06240	deoxycytidylate deaminase, putative	0,022	1,423	-0,705	-0,508	-0,171	-0,183	0,111	0,669	-0,325	-0,460	-0,037	-0,187	GO:0000004	unknown
20	Afu7g04760	gamma-glutamyltranspeptidase	-0,007	1,283	-0,649	-0,283	-0,082	0,063	-0,013	0,835	-0,332	-0,116	-0,111	-0,011	GO:0045229	cell wall and envelope biogenesis
20	Afu1g16710	fatty acid elongase (Gig30), putative	-0,013	1,470	-0,741	-0,330	-0,050	-0,232	-0,071	1,077	-0,647	0,202	0,254	0,186	GO:0006629	lipid metabolism
20	Afu6g03060	monosaccharide transporter	-0,031	1,085	-0,017	0,352	-0,469	-0,795	-0,043	-0,041	-0,530	-0,623	-0,308	-1,004	GO:0006810	transport
20	Afu2g14590	MFS monosaccharide transporter, putative	-0,072	1,701	-0,085	0,167	-0,375	-0,442	-0,015	0,160	-0,012	-0,633	-0,142	-0,299	GO:0006810	transport
20	Afu6g06590	DnaJ chaperone (Caj1), putative	0,027	0,990	-0,326	-0,005	-0,464	-0,543	0,009	0,512	-0,084	-0,133	-0,162	-0,044	GO:0006464	protein modification
20	Afu5g09070	hypothetical protein	NaN	0,688	-0,308	-0,005	-0,249	-0,310	0,036	0,362	-0,514	-0,275	-0,078	-0,443	GO:0000004	unknown
20	Afu3g13160	hypothetical protein	-0,021	1,086	0,145	-0,131	-0,414	-0,673	0,050	0,452	-0,028	-0,341	-0,145	-0,590	GO:0000004	unknown
20	Afu2g13830	conserved hypothetical protein	-0,154	1,343	0,745	-0,050	-0,208	-0,426	-0,021	0,246	-0,142	-0,539	-0,339	-0,744	GO:0000004	unknown
20	Afu8g02460	hypothetical protein	NaN	1,215	0,554	0,163	-0,177	-0,019	0,104	0,280	-0,135	-0,208	-0,279	-0,308	GO:0000004	unknown
20	Afu2g05630	hypothetical protein	-0,008	1,776	-0,325	-0,655	-0,299	0,003	-0,024	1,056	0,245	0,384	-0,003	0,189	GO:0000004	unknown
20	Afu1g03730	MFS transporter, putative	0,010	1,398	-0,164	-0,316	-0,242	0,012	-0,028	0,665	0,326	0,075	-0,169	0,154	GO:0006810	transport
20	Afu1g09300	hypothetical protein	NaN	1,096	0,099	-0,246	-0,336	-0,290	-0,027	0,907	0,099	0,121	-0,076	0,031	GO:0000004	unknown
20	Afu6g03660	onanonoxo-7-onima-8-eninoihtemlysoneda	-0,122	1,346	0,239	-0,761	-0,434	0,035	-0,040	0,874	0,348	0,032	-0,202	0,220	GO:0006732	coenzyme metabolism
20	Afu2g09630	hypothetical protein	0,037	1,631	0,410	-0,654	-0,690	-0,571	0,020	1,224	0,094	-0,194	-0,078	-0,059	GO:0000004	unknown
20	Afu6g02850	C6 finger domain protein, putative	-0,159	1,162	0,679	-0,380	-0,324	0,134	-0,032	1,566	0,233	-0,107	-0,569	0,022	GO:0045449	regulation of transcription
20	Afu5g06340	BAR domain protein	0,190	1,197	0,444	-0,214	-0,326	-0,226	0,138	1,405	0,046	-0,014	-0,045	0,120	GO:0000004	unknown
20	Afu5g07000	NAD binding Rossmann fold oxidoreductase, putative	-0,039	1,156	1,000	-0,496	-0,715	-0,189	-0,018	0,106	-0,018	-0,565	-0,779	-0,237	GO:0000004	unknown
20	Afu4g12870	methylmalonate-semialdehyde dehydrogenase, putative	-0,010	1,629	1,133	-0,470	-0,549	-0,053	-0,036	0,639	0,119	0,023	-0,305	-0,002	GO:0000004	unknown
20	Afu2g10240	NAD binding Rossmann fold oxidoreductase, putative	-0,126	1,062	0,636	-1,017	-0,701	-0,395	-0,073	0,276	0,513	0,165	-0,213	0,166	GO:0000004	unknown
20	Afu2g13850	protein phosphatase regulatory subunit (Gac1), putative	-0,010	1,322	0,996	0,134	-0,327	-0,935	0,001	0,918	-0,106	-0,225	0,018	-0,506	GO:0006464	protein modification
20	Afu3g02220	DUF427 domain protein	NaN	1,092	0,660	-0,173	-0,856	-1,022	NaN	0,663	0,514	NaN	-0,176	-0,363	GO:0000004	unknown
20	Afu2g14640	F-box domain protein	NaN	0,676	0,951	0,389	0,239	-0,017	NaN	0,383	-0,029	-0,243	-0,124	-0,022	GO:0000004	unknown
20	Afu3g06180	DNA lyase	-0,033	1,514	-0,205	0,186	0,381	0,387	-0,041	0,854	-0,530	-0,372	0,011	0,045	GO:0006259	DNA metabolism
20	Afu1g04670	related to suppressor protein PSP1	0,040	1,514	0,210	0,097	0,088	0,281	-0,062	1,050	-0,127	0,005	0,277	0,246	GO:0000004	unknown
20	Afu1g01600	deoxyribodipyrimidine photolyase	-0,041	1,291	0,080	-0,068	0,111	0,039	0,017	0,453	0,196	-0,483	-0,183	0,077	GO:0006259	DNA metabolism
20	Afu8g02120	conserved hypothetical protein	0,065	1,034	-0,105	-0,177	-0,166	-0,181	-0,012	0,709	-0,370	-0,409	-0,296	-0,185	GO:0000004	unknown
20	Afu7g02510	hypothetical protein	-0,038	0,813	0,208	-0,221	-0,257	-0,249	-0,096	0,471	-0,053	-0,319	-0,285	-0,086	GO:0000004	unknown
20	Afu7g04040	hexokinase, putative	-0,049	1,037	0,233	0,027	-0,054	-0,157	0,042	0,454	0,078	-0,072	-0,051	-0,155	GO:0005975	carbohydrate metabolism
20	Afu5g11980	MFS efflux transporter, putative	NaN	0,727	-0,064	-0,104	-0,198	-0,247	0,021	0,375	0,037	0,026	-0,158	0,044	GO:0006810	transport
20	Afu3g10870	hypothetical protein	-0,007	0,722	0,224	0,020	-0,053	-0,139	-0,024	0,101	-0,081	0,121	-0,010	-0,024	GO:0000004	unknown
20	Afu2g02130	fatty acid desaturase, putative	-0,141	1,211	-0,113	-0,002	-0,033	0,121	0,025	0,418	-0,296	-0,101	-0,094	-0,152	GO:0006629	lipid metabolism
20	Afu2g07610	alcohol dehydrogenase PAN2	NaN	0,772	-0,103	0,150	0,114	0,398	-0,106	0,588	-0,229	-0,103	0,020	0,058	GO:0006629	lipid metabolism
20	Afu4g07200	conserved hypothetical protein	-0,186	1,096	0,116	-0,033	-0,246	-0,133	-0,017	0,770	0,318	0,609	0,118	0,163	GO:0000004	unknown
20	Afu3g06660	NIPSNAP1 protein	-0,123	0,888	0,520	-0,276	-0,360	-0,173	-0,010	0,651	0,191	0,167	-0,127	0,230	GO:0000004	unknown
20	Afu4g14350	conserved hypothetical protein	NaN	0,766	0,677	-0,104	-0,207	-0,045	-0,192	0,391	0,079	0,242	-0,034	0,078	GO:0000004	unknown
20	Afu1g02330	conserved hypothetical protein	-0,053	1,121	0,667	-0,170	-0,239	-0,129	0,061	0,418	0,167	-0,060	-0,037	-0,049	GO:0000004	unknown
20	Afu2g14790	hypothetical protein	NaN	1,223	0,667	-0,037	-0,148	-0,280	0,058	0,613	0,224	0,227	0,191	-0,055	GO:0000004	unknown
20	Afu7g04140	L-galactose dehydrogenase (L-GalDH), putative	-0,003	1,080	0,469	0,019	0,111	0,297	0,063	0,659	-0,118	-0,154	0,055	0,128	GO:0006732	coenzyme metabolism
20	Afu3g01440	DUF1212 domain membrane protein	0,039	0,985	0,606	-0,129	-0,059	0,015	-0,027	0,776	0,059	0,030	0,195	0,277	GO:0000004	unknown
20	Afu4g12540	hypothetical protein	0,085	1,026	0,495	-0,062	-0,083	0,094	0,036	0,812	0,166	0,278	0,032	0,397	GO:0000004	unknown
20	Afu6g06480	hypothetical protein	NaN	0,837	0,619	0,234	0,058	-0,040	0,122	0,650	-0,024	0,145	0,032	-0,010	GO:0000004	unknown
20	Afu8g02710	C6 transcription factor, putative	0,003	1,061	0,673	0,334	0,068	-0,039	-0,034	0,578	0,064	0,209	0,060	0,046	GO:0045449	regulation of transcription
20	Afu2g12960	hypothetical protein	0,056	1,056	0,160	0,228	0,346	0,160	0,020	0,515	0,054	0,140	0,204	0,101	GO:0000004	unknown
20	Afu5g01990	BYS1 domain protein, putative	-0,073	1,019	0,253	0,281	0,359	0,230	0,012	0,316	0,022	-0,253	-0,021	0,027	GO:0000004	unknown
20	Afu4g05870	oxidoreductase, short-chain dehydrogenase/reductase family	0,053	0,868	0,344	0,375	-0,039	0,109	0,054	0,329	-0,006	-0,046	-0,058	-0,089	GO:0006629	lipid metabolism
20	Afu7g06290	Pfs, NACHT, and Ankyrin domain protein	NaN	0,860	0,390	0,166	0,265	0,116	-0,050	0,140	-0,052	0,024	0,088	-0,126	GO:0000004	unknown

21	Afu3g00730	conserved hypothetical protein	0,002	0,570	0,051	-1,150	-0,926	-1,728	-0,010	0,744	1,342	1,300	0,816	0,929	GO:0000004	unknown
21	Afu5g07320	poly(ADP)-ribose polymerase PARP, putative	NaN	1,130	0,667	-0,774	-0,997	-1,250	-0,076	0,516	2,800	0,187	-1,028	-1,270	GO:0016070	RNA metabolism
21	Afu1g05550	conserved hypothetical protein	0,014	-0,867	1,073	-0,577	-1,135	-2,190	-0,047	-0,371	0,507	-0,668	-0,589	-1,407	GO:0000004	unknown
21	Afu3g03290	hypothetical protein	0,033	-0,194	-0,508	-0,583	-0,865	-2,104	0,009	0,773	1,789	0,385	-0,175	-0,940	GO:0000004	unknown
21	Afu7g00280	conserved hypothetical protein	0,015	-0,360	-0,054	-0,203	-0,589	-1,200	-0,024	-0,306	0,919	0,286	-0,277	-0,680	GO:0000004	unknown
21	Afu4g00200	F-box domain protein	0,029	0,001	-0,432	-0,457	-0,816	-0,771	-0,067	-0,147	1,502	0,275	-0,731	-0,952	GO:0000004	unknown
21	Afu2g05060	alternative oxidase	0,034	-1,359	-0,419	-0,160	-1,272	-1,449	0,025	-0,991	1,246	0,838	-0,034	-0,288	GO:0006091	energy pathways
21	Afu2g09510	hypothetical protein	0,044	-0,949	-0,266	-1,050	-1,014	-1,111	-0,069	-0,311	1,126	0,612	-0,260	-0,414	GO:0000004	unknown
21	Afu1g14800	hypothetical protein	-0,015	0,062	-0,676	-1,657	-1,928	-1,996	-0,049	0,104	0,648	0,534	-0,922	-0,680	GO:0000004	unknown
21	Afu1g12350	extracellular fruiting body protein, putative	0,026	-0,068	-0,140	-1,007	-1,864	-1,421	-0,070	0,309	0,439	-0,150	-0,510	-0,300	GO:0000004	unknown
21	Afu7g01930	sexual development protein EsdC, putative	-0,007	-0,118	0,447	-0,118	-1,627	-2,793	0,007	0,282	0,591	0,370	-0,632	-1,200	GO:0007165	signal transduction
21	Afu4g08380	hypothetical protein	0,017	0,270	-0,112	-0,552	-1,182	-1,988	-0,029	0,553	0,451	0,206	-0,445	-0,583	GO:0000004	unknown
21	Afu4g08370	conserved hypothetical protein	-0,038	0,330	0,202	-0,252	-1,260	-2,297	0,050	0,296	0,396	0,139	-0,484	-0,717	GO:0000004	unknown
21	Afu1g04430	conserved hypothetical protein	0,010	0,257	0,479	-0,444	-1,148	-1,906	-0,095	0,363	0,443	0,068	-0,309	-0,717	GO:0000004	unknown
21	Afu5g08800	hypothetical protein	-0,004	0,314	0,651	-0,350	-1,121	-1,822	0,034	0,641	0,549	0,162	-0,336	-0,470	GO:0000004	unknown
21	Afu2g01720	conserved hypothetical protein	-0,027	0,608	0,241	-0,507	-1,519	-1,577	0,092	0,338	0,967	1,314	-0,198	-0,511	GO:0000004	unknown
21	Afu2g01710	GPI anchored protein, putative	-0,005	0,376	0,041	-1,046	-1,252	-1,053	0,005	0,471	1,246	0,738	-0,213	-0,318	GO:0000004	unknown
21	Afu2g14490	endoglucanase, putative	-0,022	0,346	0,894	-0,552	-1,553	-1,848	-0,034	1,325	1,405	0,199	-0,448	-0,187	GO:0005975	carbohydrate metabolism
21	Afu5g03010	conserved hypothetical protein	-0,146	0,499	1,144	-0,273	-1,237	-1,495	-0,034	1,132	1,480	0,462	-0,161	0,008	GO:0000004	unknown
21	Afu3g09690	extracellular thaumatin domain protein, putative	-0,036	0,308	0,904	0,293	-0,874	-1,400	0,038	0,334	0,804	0,025	-0,235	-0,693	GO:0000004	unknown
21	Afu6g11890	dynamin GTPase, putative	-0,077	0,025	0,803	0,141	-0,515	-1,232	-0,017	0,225	0,809	0,219	0,098	0,068	GO:0006810	transport
21	Afu2g10280	ketoreductase	0,015	0,673	1,272	-0,151	-0,895	-0,666	0,043	0,554	0,990	0,836	-0,476	-0,654	GO:0006629	lipid metabolism
22	Afu3g14540	heat shock protein HSP30, putative	-0,015	0,538	-0,105	-0,163	1,271	2,287	-0,027	0,257	0,695	0,562	1,606	2,803	GO:0006464	protein modification
22	Afu3g12190	RING finger domain protein, putative	-0,040	-0,635	-1,022	1,571	1,437	1,761	0,026	-1,263	-0,142	0,058	0,334	0,747	GO:0000004	unknown
22	Afu8g01690	hypothetical protein	-0,056	-1,375	-1,456	1,264	1,896	2,129	-0,009	-1,200	-0,617	0,307	0,872	1,303	GO:0000004	unknown
22	Afu5g06240	alcohol dehydrogenase, putative	0,013	-0,491	-1,908	1,133	1,851	2,762	-0,029	-0,888	-0,763	-0,301	0,888	1,086	GO:0006091	energy pathways
22	Afu8g05990	hypothetical protein	0,026	0,072	-1,711	1,341	2,213	2,324	-0,018	-1,143	-1,332	-0,535	0,684	0,478	GO:0000004	unknown
22	Afu6g09740	thioredoxin reductase GliT	NaN	0,036	-0,232	1,204	2,447	2,342	-0,050	-0,462	-0,397	0,352	1,628	1,662	GO:0019748	secondary metabolism
22	Afu3g11070	pyruvate decarboxylase PdcA, putative	-0,010	-0,109	-1,266	1,400	2,225	2,805	0,041	-0,213	-0,096	0,328	1,078	1,615	GO:0006091	energy pathways
22	Afu3g00590	Asp-hemolysin	0,043	-0,009	-0,849	1,059	2,121	2,612	0,001	-0,378	-0,418	-0,031	0,546	1,235	GO:0000004	unknown
22	Afu5g13750	caleosin domain protein	0,083	0,849	-0,632	1,878	1,902	1,709	0,028	-0,251	0,267	0,835	1,304	1,254	GO:0006629	lipid metabolism
22	Afu6g09100	hypothetical protein	-0,011	0,881	0,252	1,149	1,496	1,885	-0,040	0,191	0,196	0,526	1,004	1,471	GO:0000004	unknown
22	Afu1g07220	6-phosphofructo-2-kinase 1	-0,047	-0,283	-0,500	0,248	1,051	2,269	-0,031	-1,002	-0,415	0,027	0,912	1,777	GO:0006091	energy pathways
22	Afu5g10940	conserved hypothetical protein	-0,174	-0,545	-0,893	-0,187	0,572	1,806	-0,006	-0,758	-0,454	-0,232	0,416	1,084	GO:0000004	unknown
22	Afu4g08960	GPI anchored protein, putative	0,004	0,475	-0,274	0,267	1,423	2,191	-0,040	0,212	0,179	-0,073	0,728	1,359	GO:0000004	unknown
22	Afu5g10270	heat shock protein, HSP20 family	NaN	-0,041	0,208	0,084	0,710	1,995	0,001	0,107	0,255	0,241	0,521	1,906	GO:0006464	protein modification
22	Afu5g06910	DUF636 domain protein	NaN	0,525	-0,623	-0,030	0,592	1,587	-0,043	-0,087	0,036	0,113	0,591	1,587	GO:0000004	unknown
22	Afu3g15050	Flavin-binding monooxygenase, putative	-0,091	-0,456	0,597	0,238	1,173	1,172	-0,022	-0,069	-0,081	0,050	0,824	2,076	GO:0000004	unknown
22	Afu6g13330	conserved hypothetical protein	0,028	1,084	-1,112	1,264	1,552	2,130	-0,004	-0,111	-1,022	-0,619	0,115	0,577	GO:0000004	unknown
22	Afu7g05580	hypothetical protein	0,030	1,024	-1,065	1,445	1,611	1,760	-0,009	0,014	-0,243	-0,431	0,154	0,414	GO:0006629	lipid metabolism
22	Afu7g02220	hypothetical protein	-0,016	0,838	-0,375	1,176	1,664	1,676	-0,025	-0,050	-0,436	-0,226	0,257	0,480	GO:0000004	unknown
22	Afu2g14110	sulfur metabolite repression control protein SconB, putative	-0,228	0,922	-0,089	0,636	1,344	1,475	-0,034	0,322	-0,412	0,006	0,596	0,861	GO:0006464	protein modification
22	Afu2g10960	alcohol dehydrogenase, putative	-0,014	1,012	-0,213	0,649	1,220	1,551	-0,030	-0,353	-0,323	-0,039	0,614	0,503	GO:0005975	carbohydrate metabolism
22	Afu3g07310	conserved hypothetical protein	-0,023	0,867	-0,445	0,539	1,225	1,666	-0,028	0,009	-0,430	-0,335	0,199	0,742	GO:0000004	unknown
22	Afu5g01910	aldo-keto reductase (AKR), putative	0,015	0,271	-0,120	0,764	1,133	1,733	-0,013	-0,341	-0,462	-0,608	0,304	0,379	GO:0000004	unknown
22	Afu4g09250	hypothetical protein	-0,064	0,216	-1,289	0,846	1,364	1,711	-0,016	-0,691	-0,854	-0,372	0,215	0,611	GO:0000004	unknown
22	Afu6g01950	hypothetical protein	-0,222	0,321	-0,867	1,052	1,169	1,446	-0,049	-0,388	-0,705	-0,397	0,372	0,174	GO:0000004	unknown
22	Afu5g01800	DNA repair protein rad14	-0,008	-0,657	-1,281	0,852	1,293	1,546	-0,006	-0,561	-0,566	-0,143	0,286	0,618	GO:0006259	DNA metabolism
22	Afu7g05070	FAD dependent oxidoreductase, putative	0,041	0,383	-1,390	0,943	1,477	2,084	0,011	-0,362	-0,412	-0,143	0,391	1,119	GO:0000004	unknown
22	Afu1g07480	coproporphyrinogen III oxidase, putative	0,035	0,651	-1,489	0,790	1,347	1,699	-0,002	-0,178	-0,278	-0,071	0,302	0,573	GO:0019725	homeostasis
22	Afu1g09030	hypothetical protein	0,001	0,196	-1,294	0,365	1,538	1,897	0,003	-0,213	-0,261	0,299	0,724	1,099	GO:0000004	unknown
22	Afu3g03580	Transferase family superfamily	-0,068	0,126	-0,875	1,103	1,855	1,785	-0,033	-0,581	-0,011	0,024	0,600	0,933	GO:0000004	unknown
22	Afu4g00660	sensor histidine kinase/response regulator, putative	NaN	0,412	-0,969	1,365	1,694	1,829	-0,081	-0,809	-0,506	-0,199	0,923	1,014	GO:0006464	protein modification
22	Afu7g01010	alcohol dehydrogenase, putative	-0,124	-0,321	-0,625	0,846	1,800	2,026	-0,026	-0,720	-0,472	-0,325	0,751	0,885	GO:0006091	energy pathways
22	Afu5g09970	67 kDa myosin-cross-reactive antigen family protein	NaN	-0,282	-0,828	0,367	1,580	2,206	-0,016	-0,981	-0,629	-0,209	0,625	0,872	GO:0000004	unknown
22	Afu3g00380	beta-galactosidase, putative	NaN	-0,013	0,030	0,954	1,220	1,030	-0,010	-0,558	-0,572	0,028	1,052	1,077	GO:0005975	carbohydrate metabolism
22	Afu2g08290	conserved hypothetical protein	-0,174	0,487	-0,860	0,468	0,978	1,279	-0,036	-0,242	-0,276	0,230	0,382	0,758	GO:0000004	unknown
22	Afu3g03570	67 kDa myosin-cross-reactive antigen family protein	0,024	0,187	-0,493	0,662	1,415	1,611	-0,012	-0,316	0,046	0,100	0,736	1,174	GO:0000004	unknown
22	Afu2g13690	ubiquitin conjugating enzyme, putative	-0,046	0,018	-0,813	0,636	1,386	1,648	-0,055	-0,626	-0,612	-0,277	0,814	1,067	GO:0006464	protein modification
22	Afu2g17660	C4-dicarboxylate transporter/malic acid transport protein, putative	-0,056	-0,030	-0,745	0,479	1,189	1,703	-0,023	-0,677	-0,504	-0,292	0,465	1,123	GO:0006810	transport
22	Afu3g06020	glyoxalase family protein family	0,033	-0,122	-0,836	0,536	1,054	1,369	-0,021	-0,615	-0,040	-0,135	0,171	0,805	GO:0000004	unknown
22	Afu6g12220	isochorismatase family hydrolase, putative	NaN	0,308	-0,824	0,695	0,917	1,299	-0,018	-0,492	-0,318	-0,460	0,446	0,968	GO:0000004	unknown
22	Afu5g13740	protein phosphatase 2C, putative	NaN	-0,003	-0,600	0,114	0,816	0,872	0,113	-0,591	-0,261	0,043	0,809	1,343	GO:0006464	protein modification
22	Afu1g06020	DnaJ domain protein	-0,096	-0,171	-0,624	0,561	0,855	1,289	0,030	-0,473	-0,093	0,353	0,744	1,181	GO:0006464	protein modification
22	Afu1g07400	hypothetical protein	0,025	-0,643	-0,709	0,593	0,760	1,050	-0,001	-0,475	0,083	0,255	0,632	1,065	GO:0000004	unknown
22	Afu2g09220	hypothetical protein	0,088	-0,348	-0,839	0,294	0,655	1,187	0,041	-0,603	-0,107	0,053	0,528	0,937	GO:0000004	unknown
22	Afu3g00500	integral membrane protein	-0,034	-0,409	-0,380	0,546	0,849	1,312	-0,074	-0,371	-0,201	0,085	0,488	0,819	GO:0000004	unknown
22	Afu5g06230	gaba-specific permease	NaN	-0,365	-0,582	0,521	0,953	1,322	-0,019	-0,356	-0,265	0,085	0,594	0,811	GO:0006810	transport
22	Afu4g13780	polyphenol monooxygenase, putative	0,202	-0,251	-0,360	0,224	0,839	1,264	0,228	-0,543	-0,279	-0,074	0,416	0,683	GO:0000004	unknown
22	Afu3g02600	hypothetical protein	NaN	0,269	-0,291	0,393	1,203	1,282	0,029	-0,247	-0,297	-0,102	0,588	1,014	GO:0000004	unknown
22	Afu8g06020	glutamate decarboxylase	0,106	0,159	-0,374	0,320	1,005	1,316	0,011	-0,084	-0,204	-0,095	0,547	0,996	GO:0006519	amino acid
22	Afu6g13980	prenyltransferase, putative	NaN	-0,015	-0,274	0,372	0,846	1,340	-0,017	-0,047	0,037	0,125	0,541	1,142	GO:0019748	secondary metabolism

22	Afu6g14000	Tri7-like toxin biosynthesis protein, putative	NaN	-0,175	-0,247	0,143	0,933	1,272	0,029	-0,017	-0,086	0,238	0,608	0,928	GO:0019748	secondary metabolism
22	Afu3g12940	C6 transcription factor, putative	0,105	0,144	-0,103	0,665	0,855	1,323	-0,005	-0,261	-0,219	-0,006	0,299	0,685	GO:0019748	secondary metabolism
22	Afu1g12840	nitrite reductase	-0,091	1,427	-1,147	0,805	1,239	1,380	-0,036	-0,058	-0,141	0,308	0,544	0,632	GO:0000004	unknown
22	Afu3g08900	tubulin-specific chaperone c, putative	-0,091	1,532	-1,025	0,873	1,107	1,538	-0,036	0,429	-0,437	0,182	0,792	0,805	GO:0006464	protein modification
22	Afu2g09650	aspartate transaminase, putative	-0,008	1,006	-0,670	0,356	0,874	1,340	-0,008	0,626	0,392	0,474	0,521	1,132	GO:0006091	energy pathways
23	Afu8g07210	hydroxymethylglutaryl-CoA synthase	-0,030	-0,414	-0,144	2,018	0,109	0,257	0,023	-0,339	-0,231	1,582	1,186	1,611	GO:0006629	lipid metabolism
23	Afu2g08280	NADP-dependent malic enzyme	-0,005	-0,574	0,433	2,119	0,372	0,368	-0,055	-0,689	1,634	2,554	1,043	0,997	GO:0006519	amino acid
23	Afu2g09860	purine-cytosine permease	NaN	-0,414	0,919	0,744	0,668	0,533	0,006	-0,161	0,298	2,237	0,684	0,220	GO:0006139	nucleobase, nucleoside, nucleotide and nucleic acid metabolism
23	Afu2g16860	membrane transporter	-0,107	-0,341	1,145	0,056	-0,250	0,222	0,005	-0,019	1,532	1,510	0,515	0,215	GO:0006810	transport
23	Afu4g07630	microtubule associated protein (Ytm1), putative	-0,018	-1,387	1,363	1,159	1,021	0,289	0,023	-1,057	0,870	0,633	0,962	0,292	GO:0000004	unknown
23	Afu1g02210	60S ribosome biogenesis protein Brx1, putative	-0,036	-1,047	0,991	1,049	0,847	0,218	-0,009	-0,678	0,729	0,512	0,889	0,326	GO:0006412	protein biosynthesis
23	Afu2g16040	rRNA biogenesis protein RRP5, putative	-0,006	-1,122	1,120	0,767	0,498	-0,040	-0,043	-0,753	0,920	0,571	0,731	0,323	GO:0016070	RNA metabolism
23	Afu8g04790	ribosome biogenesis protein, putative	-0,013	-0,979	0,918	0,773	0,591	0,047	-0,011	-0,750	0,715	0,612	0,985	0,265	GO:0016070	RNA metabolism
23	Afu2g02700	mitochondrial DnaJ chaperone (Tim14), putative	0,056	-0,998	0,729	0,745	0,636	0,221	-0,046	-0,347	0,517	1,001	0,573	0,431	GO:0006259	DNA metabolism
23	Afu4g01010	C6 transcription factor, putative	NaN	-0,315	1,076	0,545	0,347	-0,154	-0,049	-0,286	0,376	0,849	0,405	0,385	GO:0045449	regulation of transcription
23	Afu3g14090	C2H2 finger domain protein, putative	0,027	0,092	2,086	0,205	0,436	0,551	-0,081	-0,065	1,123	0,524	0,250	0,361	GO:0045449	regulation of transcription
23	Afu5g07810	DUF914 domain membrane protein	0,043	-0,510	1,335	1,026	0,341	0,037	-0,056	-0,061	1,217	0,410	0,519	0,026	GO:0000004	unknown
23	Afu5g13070	hypothetical protein	0,032	-0,131	1,662	0,725	0,705	0,175	-0,143	-0,266	1,212	0,466	0,637	0,146	GO:0000004	unknown
23	Afu7g05210	CTP synthase	-0,014	-0,524	1,158	0,953	0,998	0,410	-0,048	-0,153	1,246	0,681	0,777	0,401	GO:0006139	nucleobase, nucleoside, nucleotide and nucleic acid metabolism
23	Afu3g06400	hypothetical protein	-0,077	-0,365	1,531	0,897	0,964	0,033	0,002	-0,066	1,033	0,880	0,971	0,282	GO:0000004	unknown
23	Afu4g07500	small nucleolar ribonucleoprotein complex subunit, putative	-0,144	-0,669	1,485	0,739	0,652	0,215	0,001	-0,038	0,795	0,527	0,706	0,324	GO:0016070	RNA metabolism
23	Afu6g09820	transcription factor AATF/Che-1, putative	NaN	-0,420	1,437	0,853	0,792	0,285	0,016	-0,006	0,846	0,651	0,783	0,484	GO:0046999	intracellular transport
23	Afu8g05430	hypothetical protein	-0,029	-0,791	1,552	0,936	0,823	0,044	-0,032	0,022	0,835	0,717	0,808	0,422	GO:0016070	RNA metabolism
23	Afu5g11620	pre-mRNA splicing factor RNA helicase (Prp43), putative	-0,099	-0,879	1,278	0,942	0,840	0,342	-0,001	-0,258	0,820	0,713	0,808	0,371	GO:0016070	RNA metabolism
23	Afu1g02630	periodic tryptophan protein (Pwp1), putative	-0,006	-0,721	1,341	0,894	0,911	0,294	-0,023	-0,283	0,953	0,456	0,880	0,455	GO:0016070	RNA metabolism
23	Afu2g13980	ATP dependent RNA helicase (Dbp9), putative	-0,049	-0,553	1,507	0,852	0,971	0,383	-0,110	-0,136	0,691	0,448	0,696	0,251	GO:0016070	RNA metabolism
23	Afu2g05480	DNA-directed RNA polymerase I 13.1 kDa polypeptide, putative	-0,105	-0,603	1,331	0,933	0,961	0,528	-0,036	0,058	0,803	0,647	0,829	0,367	GO:0006259	DNA metabolism
23	Afu6g14260	conserved hypothetical protein	0,055	-0,611	1,534	1,305	1,155	0,504	-0,056	-0,149	1,038	0,567	1,076	0,498	GO:0016070	RNA metabolism
23	Afu5g05710	pseudouridylate synthase family protein	-0,024	-0,628	1,702	1,402	1,431	0,654	-0,028	-0,400	1,191	0,508	1,065	0,328	GO:0016070	RNA metabolism
23	Afu1g04370	preribosome assembly and transport protein SRP40, putative	-0,040	-0,868	1,796	1,387	0,973	0,184	0,066	-0,221	1,272	0,683	0,892	0,414	GO:0016070	RNA metabolism
23	Afu6g11310	bifunctional pyrimidine biosynthesis protein (PyrABCN), putative	0,010	-0,592	1,534	1,023	0,960	0,242	-0,006	-0,601	0,879	0,602	1,047	0,324	GO:0006139	nucleobase, nucleoside, nucleotide and nucleic acid metabolism
23	Afu1g10560	GTP binding protein, putative	-0,057	-0,718	1,607	1,028	0,981	0,390	-0,068	-0,318	1,316	0,716	0,990	0,586	GO:0016070	RNA metabolism
23	Afu1g06220	ATP-dependent RNA helicase , putative	0,070	-0,879	1,624	1,083	0,970	0,425	0,002	-0,182	1,034	0,778	1,027	0,542	GO:0016070	RNA metabolism
23	Afu2g03930	small nucleolar ribonucleoprotein complex subunit, putative	-0,074	-0,553	1,654	1,080	0,926	0,404	0,081	-0,432	1,100	0,669	0,768	0,478	GO:0016070	RNA metabolism
23	Afu6g03750	amidophosphoribosyltransferase	-0,053	-0,181	1,864	1,086	0,991	0,545	0,005	0,013	1,235	0,755	1,113	0,630	GO:0000004	unknown
23	Afu1g02590	RNA polymerase I specific transcription initiation factor RRN3 superfamily	-0,052	-0,244	1,906	1,070	0,737	0,217	0,029	0,502	1,256	0,932	0,988	0,412	GO:0016070	RNA metabolism
23	Afu6g08920	assimilatory sulfite reductase	0,032	-0,445	1,165	0,414	1,406	1,209	0,008	0,193	0,691	0,298	1,178	0,871	GO:0000004	unknown
23	Afu3g12300	Ribosomal L22e protein family	0,047	-0,254	0,431	1,228	1,351	0,812	-0,050	-0,111	0,347	0,578	0,625	0,440	GO:0006412	protein biosynthesis
23	Afu4g13450	RNA methyltransferase, TrmH family family	NaN	-0,356	1,140	0,965	1,050	0,347	-0,064	-0,530	0,446	0,809	0,875	0,249	GO:0016070	RNA metabolism
23	Afu2g14180	conserved hypothetical protein	NaN	-0,168	0,963	1,020	0,707	0,476	0,003	-0,451	0,077	0,223	0,635	0,101	GO:0000004	unknown
23	Afu3g01060	hypothetical protein	-0,034	-0,307	1,258	1,263	1,101	0,493	-0,039	-0,397	-0,072	0,250	0,484	0,488	GO:0000004	unknown
23	Afu1g02610	conserved hypothetical protein	-0,011	-0,659	1,093	1,145	0,943	0,214	0,033	-0,321	0,474	0,025	0,394	-0,042	GO:0016070	RNA metabolism
23	Afu1g06230	ssf2 protein	-0,065	-0,824	0,935	0,912	0,852	0,316	0,018	-0,599	0,661	0,021	0,627	-0,101	GO:0016070	RNA metabolism
23	Afu4g08190	ribosome biogenesis protein Pescadillo, putative	-0,018	-0,740	1,200	0,940	0,888	0,301	0,009	-0,506	0,888	0,311	0,644	0,043	GO:0016070	RNA metabolism
23	Afu6g11070	DEAD box RNA helicase (Hca4), putative	-0,216	-0,657	1,352	0,830	0,764	0,291	-0,029	-0,218	0,545	0,035	0,676	0,167	GO:0016070	RNA metabolism
23	Afu1g15030	SDA1 domain protein	-0,009	-0,580	1,521	1,166	1,184	0,371	-0,066	-0,437	0,841	0,177	0,629	0,216	GO:0007049	cell cycle
23	Afu2g17060	60S ribosome subunit biogenesis protein (Nip7), putative	-0,036	-0,465	1,249	1,151	1,205	0,357	-0,084	-0,387	0,704	0,179	0,878	-0,008	GO:0006412	protein biosynthesis
23	Afu1g01990	WD repeat protein	0,100	-0,937	1,309	0,669	0,691	0,112	-0,002	-0,388	0,820	0,454	0,465	0,115	GO:0000004	unknown
23	Afu3g09340	PDCD2_C domain protein, putative	0,003	-0,510	1,349	0,622	0,645	0,225	-0,015	-0,285	0,588	0,246	0,479	0,139	GO:0000004	unknown
23	Afu1g14770	tRNA dihydrouridine synthase, putative	NaN	-0,533	1,166	0,531	0,723	0,185	-0,006	-0,363	0,754	0,446	0,663	0,225	GO:0016070	RNA metabolism
23	Afu2g01330	exosome complex subunit Rrp46, putative	-0,023	-0,519	0,802	0,778	0,652	0,114	-0,066	-0,202	0,543	0,416	0,588	0,100	GO:0016070	RNA metabolism
23	Afu1g14220	fibrillarin	-0,042	-0,700	0,712	0,831	0,644	0,232	-0,106	-0,569	0,675	0,445	0,828	0,181	GO:0016070	RNA metabolism
23	Afu7g01550	mitochondrial exoribonuclease Cyt-4, putative	-0,081	-0,666	0,944	0,814	0,711	0,312	-0,072	-0,489	0,520	0,394	0,748	0,325	GO:0016070	RNA metabolism
23	Afu4g07570	WD repeat protein	-0,017	-0,763	1,008	1,006	1,047	0,262	0,021	-0,288	0,642	0,364	0,620	0,330	GO:0006412	protein biosynthesis
23	Afu1g12730	mitochondrial large ribosomal subunit protein L3, putative	-0,066	-0,547	0,878	0,803	0,789	0,566	-0,054	-0,275	0,625	0,421	0,629	0,507	GO:0006412	protein biosynthesis
23	Afu2g16170	DNA-directed RNA polymerase III RPC4, putative	0,150	-0,570	0,947	0,847	0,764	0,386	0,079	-0,257	0,588	0,338	0,550	0,435	GO:0006259	DNA metabolism
23	Afu2g13020	mitochondrial dicarboxylate carrier, putative	NaN	-0,716	0,967	0,561	0,463	0,278	0,041	-0,242	0,649	0,489	0,648	0,554	GO:0046907	intracellular transport
23	Afu2g12150	midasin, putative	NaN	-0,445	0,884	0,737	0,500	0,090	-0,041	-0,398	0,800	0,714	0,598	0,388	GO:0016070	RNA metabolism
23	Afu3g06320	conserved hypothetical protein	0,143	-0,461	1,092	0,815	0,557	0,313	0,009	-0,118	0,937	0,624	0,605	0,353	GO:0000004	unknown
23	Afu4g13410	conserved hypothetical protein	-0,069	-0,473	0,555	0,763	0,881	0,550	-0,008	-0,189	0,420	0,233	0,556	0,451	GO:0006412	protein biosynthesis
23	Afu6g04440	glucose inhibited division protein A	-0,036	-0,230	0,836	0,817	0,589	0,570	0,013	0,191	0,548	0,725	0,665	0,573	GO:0006412	protein biosynthesis
23	Afu5g05930	RNA processing factor 1	-0,022	-0,517	1,086	0,961	0,899	0,595	0,058	-0,044	0,544	0,383	0,901	0,394	GO:0016070	RNA metabolism
23	Afu6g04610	DNA-directed RNA polymerase I and III 14 KDA polypeptide	0,017	-0,419	1,012	0,792	0,914	0,311	-0,028	-0,120	0,720	0,657	1,022	0,513	GO:0006259	DNA metabolism
23	Afu2g14670	eukaryotic translation initiation factor 3 subunit EifCd, putative	0,084	-0,310	1,311	0,826	0,968	0,539	-0,038	-0,138	0,772	0,191	0,591	0,459	GO:0006412	protein biosynthesis
23	Afu4g12110	TPR domain protein	-0,003	-0,412	1,123	0,892	1,041	0,483	0,021	-0,287	0,796	0,284	0,703	0,325	GO:0000004	unknown
23	Afu2g05560	exonuclease, putative	-0,071	-0,479	1,293	1,028	1,194	0,640	-0,057	-0,230	0,747	0,420	0,804	0,186	GO:0016070	RNA metabolism
23	Afu2g05430	Uridine kinase	-0,071	-0,275	1,314	1,053	1,250	0,817	-0,021	-0,092	0,813	0,526	0,685	0,393	GO:0006139	nucleobase, nucleoside, nucleotide and nucleic acid metabolism
23	Afu1g09440	ribosomal protein S23 (S12)	-0,026	-0,051	0,981	1,073	1,047	0,790	0,184	-0,057	0,452	0,453	0,629	0,429	GO:0006412	protein biosynthesis
23	Afu1g09710	hypothetical protein	0,011	-0,020	0,781	0,683	0,823	0,318	0,078	0,216	0,882	0,265	0,647	0,125	GO:0000004	unknown
23	Afu1g02460	DNA-directed RNA polymerase III 130 kd polypeptide	-0,024	-0,325	1,651	0,619	0,573	0,259	0,044	-0,013	0,931	0,481	0,471	0,432	GO:0006259	DNA metabolism
23	Afu7g01850	RNA polymerase Rpc34 subunit superfamily	0,000	-0,493	1,128	0,524	0,722	0,256	-0,032	0,049	0,732	0,671	0,557	0,425	GO:0006259	DNA metabolism
23	Afu3g06580	WD repeat protein	0,037	-0,350	1,453	0,554	0,634	0,309	-0,022	0,115	0,810	0,711	0,691	0,380	GO:0000004	unknown

23	Afu5g13610	conserved hypothetical protein	-0,012	-0,131	1,058	0,668	0,666	0,078	0,013	-0,076	0,675	0,371	0,482	0,070	GO:0000004	unknown
23	Afu5g06520	low-temperature viability protein ltv1	NaN	-0,205	1,304	0,618	0,397	0,142	-0,015	-0,015	0,635	0,366	0,436	0,145	GO:0000004	unknown
23	Afu1g06290	small nucleolar ribonucleoprotein complex subunit (SOF1), putative	-0,053	-0,056	1,446	0,697	0,599	0,271	-0,048	0,107	0,609	0,470	0,555	0,223	GO:0016070	RNA metabolism
23	Afu1g08870	hypothetical protein	0,032	-0,304	1,305	0,600	0,611	0,497	-0,052	0,220	0,679	0,349	0,425	0,167	GO:0000004	unknown
23	Afu1g00960	LINE-1 class reverse transcriptase, RNaseH, putative	-0,049	-0,080	1,199	0,514	0,786	0,643	-0,057	0,023	0,663	0,214	0,579	0,539	GO:0006313	DNA transposition
23	Afu2g11450	DUF691 domain protein	0,131	-0,082	0,979	0,717	0,888	0,433	-0,041	-0,040	0,514	0,184	0,665	0,262	GO:0016070	RNA metabolism
23	Afu2g14090	extragenic suppressor of the bimD6 mutation	0,142	-0,076	1,001	0,613	0,746	0,518	-0,005	0,118	0,375	0,133	0,236	0,167	GO:0016070	RNA metabolism
23	Afu1g05560	GTP binding protein, putative	0,025	-0,089	1,182	0,661	0,673	0,496	0,022	-0,054	0,340	0,200	0,443	0,215	GO:0000004	unknown
23	Afu5g00760	chitin synthase C	0,079	-0,045	1,092	0,717	0,739	0,285	-0,038	0,113	0,225	0,521	0,516	0,237	GO:0045229	cell wall and envelope biogenesis
23	Afu5g02890	conserved hypothetical protein	-0,078	0,081	1,192	0,782	0,796	0,424	-0,043	0,023	0,377	0,375	0,651	0,395	GO:0000004	unknown
23	Afu1g07080	tRNA-specific adenosine-34 deaminase subunit Tad3, putative	-0,036	-0,018	1,199	0,615	0,663	0,415	-0,071	0,158	0,435	0,475	0,528	0,456	GO:0016070	RNA metabolism
23	Afu6g02220	MFS toxin efflux pump, putative	-0,055	0,512	1,180	0,569	0,582	0,406	-0,042	-0,307	0,172	0,306	0,812	0,529	GO:0006810	transport
23	Afu1g13660	phenol 2-monooxygenase, putative	NaN	0,498	1,311	0,542	1,063	0,646	-0,083	-0,276	-0,079	0,384	0,806	0,871	GO:0000004	unknown
23	Afu3g08990	hypothetical protein	NaN	0,024	1,281	0,647	0,059	0,164	0,024	0,259	0,604	0,780	0,795	0,751	GO:0045229	cell wall and envelope biogenesis
23	Afu2g02190	Hypothetical nuclear protein	NaN	-0,179	1,559	0,780	0,448	0,180	0,076	NaN	0,491	0,374	0,231	-0,212	GO:0016070	RNA metabolism
23	Afu3g08530	MFS drug transporter, putative	0,006	-0,164	0,973	0,278	0,056	0,078	0,005	0,240	0,640	0,552	0,193	0,286	GO:0006810	transport
23	Afu3g02910	dihydroxy-acetone synthase, putative	NaN	-0,014	1,031	0,223	0,268	-0,086	0,032	0,348	0,762	0,763	0,311	0,169	GO:0006091	energy pathways
23	Afu5g02070	hypothetical protein	-0,166	-0,034	0,993	0,839	0,427	-0,070	0,040	0,138	0,451	0,365	0,309	0,078	GO:0000004	unknown
23	Afu5g02790	amino acid permease	-0,011	0,079	1,062	0,728	0,467	0,204	-0,061	0,136	0,416	0,219	0,296	0,343	GO:0006810	transport
23	Afu6g03040	MSF multidrug transporter, putative	NaN	-0,183	1,164	0,505	0,226	0,132	0,075	0,158	0,356	0,567	0,362	0,237	GO:0006810	transport
23	Afu5g03510	PQ loop repeat protein	-0,072	-0,015	0,816	0,603	0,286	0,163	0,011	0,303	0,491	0,511	0,398	0,397	GO:0000004	unknown
23	Afu4g11970	metallothionein-I gene transcription activator	-0,151	0,146	0,969	0,454	0,396	0,014	0,106	0,205	0,434	0,477	0,475	0,233	GO:0045449	regulation of transcription
23	Afu2g15090	hypothetical protein	-0,020	0,245	1,424	0,609	0,126	0,213	0,057	0,266	0,773	0,503	0,168	0,161	GO:0006950	response to stress
23	Afu1g15840	hypothetical protein	NaN	0,139	1,277	0,498	0,463	0,291	0,045	0,343	0,663	0,396	0,280	0,140	GO:0000004	unknown
23	Afu4g10520	CSL family zinc fnger-containing protein	NaN	0,124	1,176	0,311	0,540	0,353	-0,103	0,352	0,542	0,470	0,554	0,323	GO:0000004	unknown
23	Afu3g12000	hypothetical protein	0,128	0,107	1,312	0,493	0,353	0,043	-0,024	0,235	0,995	0,697	0,580	0,243	GO:0000004	unknown
23	Afu2g11730	Protein kinase domain-containing protein	-0,022	0,166	1,548	0,385	0,287	-0,147	0,022	0,542	1,281	0,820	0,528	0,193	GO:0000004	unknown
23	Afu3g05320	C2H2 finger domain protein, putative	0,002	0,326	1,573	0,541	0,592	0,213	-0,107	0,766	1,259	0,406	0,506	0,257	GO:0045449	regulation of transcription
23	Afu5g12910	small nuclear ribonucleoprotein SmD2, putative	0,003	0,421	0,940	0,748	0,918	0,319	-0,007	0,650	0,631	0,692	0,654	0,447	GO:0016070	RNA metabolism
23	Afu2g02150	ribosomal protein S10	-0,063	0,140	1,577	1,077	1,204	0,822	-0,049	0,434	0,825	0,710	0,835	0,511	GO:0006412	protein biosynthesis
23	Afu6g03050	oleate delta-12 desaturase	0,132	0,460	1,426	0,965	1,203	0,977	0,031	0,388	0,623	0,403	0,628	0,479	GO:0000004	unknown
23	Afu5g13600	vacuolar protein sorting vps16, putative	-0,052	-0,091	1,418	1,080	0,958	0,270	-0,121	-0,085	0,859	0,596	0,681	0,183	GO:0046915	intracellular transport
23	Afu3g00770	hypothetical protein	-0,022	0,108	1,406	0,971	1,045	0,142	0,049	-0,040	0,538	0,430	0,755	0,083	GO:0000004	unknown
23	Afu1g16290	ATP dependent RNA helicase (Rok1), putative	-0,132	0,189	1,185	0,909	0,774	0,260	-0,056	0,244	0,613	0,370	0,890	0,289	GO:0016070	RNA metabolism
23	Afu3g06380	hypothetical protein	-0,010	-0,153	1,399	1,042	0,937	0,287	0,035	0,290	0,931	0,746	0,858	0,428	GO:0000004	unknown
23	Afu7g02210	conserved hypothetical protein	-0,036	0,037	1,530	0,975	0,800	0,316	0,069	0,237	0,767	0,481	0,442	0,287	GO:0016070	RNA metabolism
23	Afu3g11110	Pumilio-family RNA binding repeat domain protein	0,011	0,002	1,525	0,914	0,735	0,288	0,009	0,375	0,800	0,701	0,477	0,467	GO:0016070	RNA metabolism
23	Afu1g01030	reverse transcriptase, putative	-0,005	-0,027	1,520	0,730	0,805	0,692	-0,069	0,014	0,940	0,521	0,812	0,573	GO:0006313	DNA transposition
23	Afu5g12850	las1 protein	0,446	0,266	1,370	0,762	0,856	0,556	0,006	0,521	0,796	0,785	0,869	0,410	GO:0007165	signal transduction
23	Afu5g09620	gcd14 protein	0,267	0,245	1,441	0,913	1,055	0,548	-0,006	0,610	1,022	0,617	0,772	0,555	GO:0016070	RNA metabolism
23	Afu6g04420	maintaining mitochondrial morphology protein MMM1	-0,058	0,301	1,408	0,838	0,678	0,538	-0,010	0,395	0,745	0,686	0,783	0,499	GO:0006996	organelle organization and biogenesis
23	Afu5g10860	hypothetical protein	NaN	0,376	1,426	0,907	0,815	0,441	0,044	0,409	1,026	0,615	1,102	0,655	GO:0000004	unknown
24	Afu1g13550	hypothetical protein	-0,025	0,366	-0,828	0,696	0,550	0,803	-0,024	0,196	-0,932	-1,590	-0,809	0,257	GO:0000004	unknown
24	Afu4g12590	conserved hypothetical protein	-0,258	1,252	-0,710	0,585	0,209	0,651	-0,017	-0,050	-0,875	-1,211	-0,773	-0,312	GO:0000004	unknown
24	Afu5g13100	hypothetical protein	0,185	1,015	-0,226	0,186	0,388	0,959	0,051	0,589	-0,442	-0,940	-0,295	0,495	GO:0000004	unknown
24	Afu7g02110	RanBPM	0,002	1,087	-1,243	-0,035	0,464	0,664	-0,022	0,007	-0,664	-0,537	-0,053	-0,037	GO:0007165	signal transduction
24	Afu7g00650	hypothetical protein	NaN	1,032	-0,640	-0,210	0,388	0,344	-0,056	-0,215	-0,311	-0,544	-0,107	-0,152	GO:0000004	unknown
24	Afu7g05920	stearic acid desaturase (SdeA), putative	-0,011	1,043	-1,252	0,081	0,155	0,213	-0,022	0,233	-0,283	0,119	0,218	0,050	GO:0006629	lipid metabolism
24	Afu1g12250	mitochondrial hypoxia responsive domain protein	0,249	0,557	-0,493	0,353	0,270	0,482	-0,018	-0,283	-0,620	-0,182	-0,263	-0,391	GO:0000004	unknown
24	Afu8g05140	oxidoreductin	0,005	1,701	-1,264	0,615	0,844	0,703	-0,043	0,135	-0,341	0,040	0,233	0,288	GO:0006464	protein modification
24	Afu7g01430	opsin 1	-0,011	0,775	-0,668	0,634	0,516	0,677	0,031	-0,501	-0,315	-0,391	0,354	0,518	GO:0006810	transport
24	Afu1g12830	nitrate reductase NiaD	0,115	1,263	-0,430	0,423	0,910	1,188	-0,038	-0,240	-0,499	-0,189	0,135	0,161	GO:0000004	unknown
24	Afu1g17040	D-lactate dehydrogenase	0,049	1,179	-0,583	0,658	0,780	1,327	0,039	-0,027	-0,211	-0,250	0,291	0,574	GO:0006519	amino acid
24	Afu6g03400	hypothetical protein	-0,016	0,347	-2,226	0,545	1,060	1,561	-0,017	-0,622	-1,146	-0,588	0,295	0,266	GO:0019748	secondary metabolism
24	Afu4g13630	PE repeat family protein	-0,019	0,069	-1,842	-0,267	0,227	0,857	-0,020	-0,237	-0,316	-0,386	-0,120	0,360	GO:0000004	unknown
24	Afu5g14680	hypothetical protein	0,023	-0,216	-2,129	0,304	0,600	1,059	0,011	-0,554	-0,929	-0,371	-0,250	0,113	GO:0000004	unknown
24	Afu3g07980	conserved hypothetical protein	-0,021	-0,235	-1,748	0,042	0,484	1,217	-0,062	-0,840	-0,791	-0,534	-0,364	0,423	GO:0000004	unknown
24	Afu2g09530	PUTATIVE SIGNAL PEPTIDE PROTEIN	0,027	-0,399	-1,545	0,249	0,727	1,336	-0,026	-0,748	-0,827	-0,586	0,012	0,326	GO:0000004	unknown
24	Afu6g10080	conserved hypothetical protein	0,031	0,118	-1,522	0,201	0,913	1,390	-0,017	-0,019	-0,733	-0,770	-0,008	0,368	GO:0000004	unknown
24	Afu5g11820	conserved hypothetical protein	0,060	-0,665	-0,870	0,238	0,303	0,712	-0,116	-0,758	-0,658	-0,524	0,038	-0,006	GO:0000004	unknown
24	Afu5g08640	hypothetical protein	0,013	-0,707	-1,373	0,809	0,745	0,909	-0,038	-1,369	-0,685	-0,311	0,264	0,320	GO:0000004	unknown
24	Afu5g14200	hypothetical protein	-0,436	-0,004	-0,526	0,835	0,771	0,865	-0,019	-0,748	-0,970	-0,515	0,317	0,287	GO:0000004	unknown
24	Afu3g10750	acetate kinase, putative	0,062	-0,110	-1,373	0,708	0,860	0,741	-0,002	-0,631	-0,841	-0,424	0,509	0,624	GO:0000004	unknown
24	Afu7g02030	cytochrome c oxidase assembly protein (Pet117), putative	-0,032	-0,290	-0,903	0,507	0,951	0,466	-0,082	-0,300	-0,484	-0,014	0,633	0,319	GO:0006091	energy pathways
24	Afu2g15490	TPR domain protein	0,015	0,046	-0,754	-0,100	0,695	1,103	-0,005	-0,399	-0,683	-0,404	-0,039	0,568	GO:0000004	unknown
24	Afu2g13870	mitochondrial carrier protein, putative	-0,297	0,688	-0,839	NaN	0,481	0,869	0,012	-0,255	-0,643	-0,375	0,129	0,383	GO:0046907	intracellular transport
24	Afu4g12930	ubiquinone biosynthesis monooxygenase (Coq6), putative	-0,025	0,566	-0,661	0,224	0,797	1,133	0,018	-0,411	-0,656	-0,545	-0,039	0,262	GO:0006732	coenzyme metabolism
24	Afu1g06820	conserved hypothetical protein	-0,042	0,199	-1,121	-0,075	0,224	0,711	-0,014	-0,508	-0,935	-0,492	-0,183	0,147	GO:0000004	unknown
24	Afu2g09670	SNARE protein (Ufe1), putative	NaN	0,333	-0,494	0,119	0,319	0,706	0,071	-0,627	-0,500	-0,521	-0,069	-0,131	GO:0047056	intracellular transport
24	Afu1g04970	Patatin-like serine hydrolase, putative	0,051	0,089	-0,749	0,206	0,240	0,486	0,038	-0,475	-0,490	-0,174	-0,076	0,124	GO:0000004	unknown
24	Afu2g17100	conserved hypothetical protein	0,095	0,012	-0,829	0,201	0,584	0,789	-0,063	-0,556	-0,747	-0,515	-0,161	0,041	GO:0000004	unknown
24	Afu4g06410	hypothetical protein	-0,001	0,049	-0,950	0,480	0,583	0,981	0,004	-0,540	-0,663	-0,522	0,033	0,419	GO:0000004	unknown

24	Afu6g04370	hypothetical protein	-0,071	-0,216	-0,748	0,302	0,667	0,598	0,009	-0,685	-0,701	-0,474	0,232	0,020	GO:0000004	unknown
24	Afu2g02430	conserved hypothetical protein	0,001	-0,069	-0,415	0,370	0,640	0,789	-0,001	-0,531	-0,698	-0,257	0,120	0,093	GO:0000004	unknown
24	Afu1g15270	ATP-dependent Clp protease, putative	0,019	0,290	-1,091	-0,203	0,310	0,999	-0,016	-0,690	-0,318	-0,295	0,407	0,558	GO:0030163	protein catabolism
24	Afu1g17350	CP2 transcription factor, putative	0,013	0,300	-0,954	-0,256	0,631	1,635	-0,037	-0,213	-0,324	-0,012	0,224	0,653	GO:0045449	regulation of transcription
24	Afu2g00440	hypothetical protein	NaN	-0,185	-0,504	0,185	0,510	0,883	-0,058	-0,276	-0,279	-0,282	0,251	1,060	GO:0000004	unknown
24	Afu2g05810	dienelactone hydrolase	0,000	-0,319	-0,682	0,161	0,310	0,677	-0,061	-0,411	-0,343	-0,196	0,192	0,526	GO:0000004	unknown
24	Afu5g10950	hypothetical protein	-0,025	-0,454	-0,211	0,157	0,291	0,897	-0,014	-0,570	-0,395	-0,120	0,143	0,473	GO:0000004	unknown
24	Afu6g12160	C6 transcription factor, putative	0,050	-0,582	-0,470	-0,111	0,627	1,243	0,035	-0,571	-0,137	-0,247	0,426	0,795	GO:0045449	regulation of transcription
24	Afu3g03560	pyridine nucleotide-disulfide oxidoreductase, putative	NaN	-0,132	-0,405	0,061	0,913	1,054	0,009	-0,505	-0,340	-0,375	0,381	0,732	GO:0000004	unknown
24	Afu2g05170	hypothetical protein	NaN	-0,186	-0,487	0,303	0,808	1,130	-0,035	-0,647	-0,244	-0,208	0,268	0,314	GO:0000004	unknown
24	Afu1g04450	siderophore biosynthesis protein, putative	-0,047	-0,157	-0,248	0,273	0,556	0,963	0,002	-0,600	-0,248	-0,199	0,233	0,498	GO:0019748	secondary metabolism
24	Afu3g13830	PH domain protein	-0,067	-0,129	-0,570	0,711	0,851	1,221	0,018	-0,677	-0,355	-0,169	0,171	0,512	GO:0045449	regulation of transcription
24	Afu6g02180	GNAT family acetyltransferase, putative	-0,130	-0,053	-0,614	0,653	0,818	0,961	-0,043	-0,617	-0,326	0,005	0,430	0,619	GO:0000004	unknown
24	Afu3g11550	LEA domain protein	-0,111	0,221	-0,139	0,536	0,882	1,029	-0,002	-0,196	-0,556	-0,471	0,184	0,814	GO:0006810	transport
24	Afu1g04550	HMG box protein, putative	-0,083	0,189	-0,406	0,013	0,871	0,759	-0,009	-0,078	-0,472	-0,502	-0,153	0,226	GO:0045449	regulation of transcription
24	Afu5g11760	hydroxymethylbilane synthase, putative	0,002	0,313	-0,540	-0,001	0,368	0,812	0,004	0,062	-0,470	-0,357	0,031	0,281	GO:0000004	unknown
24	Afu5g11770	hypothetical protein	0,040	0,451	-0,352	0,130	0,567	0,913	-0,065	0,071	-0,484	-0,410	-0,049	0,205	GO:0000004	unknown
24	Afu1g16480	acid phosphatase, putative	0,006	0,343	-0,411	0,060	0,697	0,789	-0,018	-0,036	-0,265	-0,397	0,143	0,222	GO:0000004	unknown
24	Afu5g03850	class III chitinase, putative	NaN	0,345	-0,458	0,581	0,597	0,592	-0,273	0,032	-0,244	-0,145	0,082	0,246	GO:0045229	cell wall and envelope biogenesis
25	Afu6g13560	hypothetical protein	NaN	-0,226	-0,106	1,128	-0,122	0,086	0,091	0,206	0,097	0,081	-0,089	-0,051	GO:0000004	unknown
25	Afu2g16340	hypothetical protein	0,082	-0,701	0,612	0,083	-0,161	-0,661	-0,047	-0,560	0,335	0,123	0,264	-0,191	GO:0000004	unknown
25	Afu1g05300	capsular associated protein, putative	0,084	-1,208	0,605	0,077	-0,217	-0,671	-0,057	-0,657	0,642	-0,014	-0,214	-0,402	GO:0045229	cell wall and envelope biogenesis
25	Afu6g02320	hypothetical protein	0,010	-1,138	0,803	-0,086	-0,205	-0,372	-0,056	-0,677	0,230	-0,194	-0,121	-0,372	GO:0000004	unknown
25	Afu6g11110	C6 zinc cluster transcription factor, putative	0,091	-0,844	0,607	0,004	-0,322	-0,446	0,028	-1,088	-0,174	-0,087	-0,262	-0,708	GO:0045449	regulation of transcription
25	Afu2g04270	mitochondrial inner membrane translocase subunit (TIM17), putative	-0,002	-1,096	-0,190	0,439	-0,111	-0,780	-0,012	-0,601	0,337	0,531	0,461	-0,068	GO:0046907	intracellular transport
25	Afu1g07140	c-24(28) sterol reductase	-0,072	-1,664	0,625	0,654	0,248	-0,079	-0,116	-1,116	0,456	0,219	0,156	-0,242	GO:0006629	lipid metabolism
25	Afu8g06090	amino acid permease, putative	0,023	-1,054	0,645	-0,110	0,144	0,309	0,152	-0,068	-0,173	-0,167	-0,181	-0,105	GO:0006810	transport
25	Afu2g11290	orotate phosphoribosyltransferase	0,042	-0,881	0,271	0,074	0,240	0,221	0,046	-0,445	0,138	-0,461	-0,086	-0,076	GO:0000004	unknown
25	Afu5g08300	alpha-actinin, sarcomeric (f-actin cross linking protein)	0,057	-0,974	0,716	0,242	0,093	-0,104	-0,015	-0,366	0,156	-0,021	0,044	-0,143	GO:0051301	cell division
25	Afu6g10730	putative nucleoporin c22g709c	-0,043	-0,961	-0,136	0,319	0,227	-0,127	0,017	-0,629	-0,437	-0,262	0,046	-0,222	GO:0046907	intracellular transport
25	Afu6g10970	conserved hypothetical protein	-0,013	-0,947	0,071	0,154	0,094	0,079	-0,018	-0,494	-0,265	0,007	-0,234	-0,241	GO:0000004	unknown
25	Afu5g08510	dynein family protein	-0,039	-0,942	-0,045	0,126	-0,013	-0,052	0,030	-0,489	-0,024	-0,107	-0,139	-0,228	GO:0051301	cell division
25	Afu2g12830	UDP-glucoronosyl and UDP-glucosyl transferase family	NaN	-1,059	0,018	0,028	-0,202	0,082	0,039	-0,086	-0,083	0,070	0,069	-0,011	GO:0000004	unknown
25	Afu6g08580	FKBP-type peptidyl-prolyl isomerase, putative	-0,014	-1,265	0,095	0,592	0,546	-0,057	0,049	-0,953	0,008	-0,136	0,367	-0,142	GO:0000004	unknown
25	Afu1g14180	methyltransferase (Ncl1), putative	0,049	-0,996	0,011	0,409	0,362	-0,143	0,011	-0,704	0,080	-0,105	0,363	-0,089	GO:0016070	RNA metabolism
25	Afu5g03090	small nucleolar ribonucleoprotein complex subunit (Pwp2), putative	-0,014	-1,042	0,511	0,510	0,218	-0,269	-0,042	-0,846	0,259	0,182	0,414	-0,062	GO:0016070	RNA metabolism
25	Afu1g13100	nucleoporin (Nup184), putative	-0,071	-0,937	0,000	0,241	-0,023	-0,189	0,017	-0,720	0,277	0,241	0,229	-0,020	GO:0046907	intracellular transport
25	Afu1g15790	ecylglycaid-PDC	-0,069	-0,852	0,485	0,311	-0,055	-0,147	-0,007	-0,494	0,246	0,395	-0,009	-0,076	GO:0006629	lipid metabolism
25	Afu2g11080	glucosyltransferase (Die2), putative	-0,159	-0,908	0,281	0,278	-0,049	-0,134	0,044	-0,645	-0,060	0,146	0,077	-0,163	GO:0006464	protein modification
25	Afu6g10790	MFS amine transporter, putative	-0,043	-0,951	0,120	0,599	0,059	-0,368	0,030	-0,472	0,046	0,210	0,212	-0,247	GO:0006810	transport
25	Afu2g02350	MYB DNA-binding domain protein	-0,050	-0,971	0,120	0,311	0,280	-0,006	0,029	-0,302	0,129	0,076	0,318	-0,010	GO:0000004	unknown
25	Afu2g09160	sua5 protein	-0,243	-0,636	0,264	0,421	0,194	-0,227	-0,029	-0,593	0,118	-0,113	0,255	-0,006	GO:0006412	protein biosynthesis
25	Afu4g12740	tRNA (adenine-N(1)-)-methyltransferase	-0,137	-0,760	0,387	0,466	0,227	-0,036	-0,057	-0,431	0,099	0,078	0,375	0,092	GO:0016070	RNA metabolism
25	Afu6g10460	ceramide synthase membrane component (Lag1), putative	0,344	-0,929	1,227	0,417	-0,020	-0,273	0,062	-0,536	0,488	0,341	0,102	-0,165	GO:0006629	lipid metabolism
25	Afu3g11880	hypothetical protein	-0,052	-0,974	1,172	0,916	0,242	-0,279	0,001	-0,504	0,428	0,201	0,285	-0,121	GO:0000004	unknown
25	Afu6g13440	choline sulfatase, putative	NaN	-0,609	0,989	0,331	0,123	0,183	0,099	-0,300	0,435	0,564	0,194	0,112	GO:0000004	unknown
25	Afu2g13130	3' exoribonuclease family protein (Rrp42), putative	0,071	-0,488	0,781	0,756	0,312	0,153	0,010	-0,172	0,351	0,253	0,376	0,087	GO:0016070	RNA metabolism
25	Afu2g03740	exosome complex endonuclease 1/ribosomal RNA processing protein, putative	-0,100	-0,585	1,033	0,653	0,570	0,184	0,088	-0,333	0,269	0,327	0,356	0,013	GO:0016070	RNA metabolism
25	Afu1g13200	conserved hypothetical protein	-0,028	-1,025	1,050	0,581	0,331	-0,159	0,044	-0,631	0,665	0,643	0,672	0,187	GO:0000004	unknown
25	Afu1g10470	conserved hypothetical protein	-0,090	-1,006	0,727	0,806	0,711	0,161	-0,100	-0,609	0,178	0,492	0,422	0,229	GO:0000004	unknown
25	Afu4g07540	small nucleolar ribonucleoprotein complex subunit, putative	-0,030	-1,076	0,913	0,877	0,758	0,073	-0,035	-0,946	0,543	0,398	0,762	0,172	GO:0016070	RNA metabolism
25	Afu2g13040	mitochondrial co-chaperone GrpE, putative	-0,027	-1,089	0,409	0,842	0,909	0,420	-0,029	-0,611	0,248	0,720	0,907	0,637	GO:0046907	intracellular transport
25	Afu3g09040	conserved hypothetical protein	0,026	-1,063	0,511	0,951	1,045	0,322	-0,034	-0,945	0,215	0,106	0,605	-0,146	GO:0000004	unknown
25	Afu3g14350	GTP binding protein Guf1, putative	0,086	-0,624	0,519	0,776	0,827	0,422	-0,071	-0,585	0,059	0,087	0,589	-0,060	GO:0000004	unknown
25	Afu4g11900	TIM54	0,139	-0,654	0,439	0,726	0,701	0,401	0,100	-0,526	-0,047	0,271	0,480	0,283	GO:0046907	intracellular transport
25	Afu5g13510	cell cycle control protein (Cwf8), putative	-0,028	-0,398	0,443	0,718	0,681	0,496	-0,005	-0,344	0,190	0,241	0,366	0,307	GO:0007049	cell cycle
25	Afu5g11740	mitochondrial ribosomal protein L23, putative	-0,014	-0,580	0,347	0,627	0,902	0,626	-0,026	-0,336	0,203	0,184	0,614	0,380	GO:0006412	protein biosynthesis
25	Afu2g16970	mitochondrial large ribosomal subunit L30, putative	0,078	-0,589	0,363	0,588	0,774	0,456	-0,088	-0,314	0,199	0,135	0,530	0,192	GO:0006412	protein biosynthesis
25	Afu5g12800	50S ribosomal protein L4	0,055	-0,533	0,383	0,537	0,809	0,528	-0,054	-0,267	0,255	-0,047	0,471	0,285	GO:0006412	protein biosynthesis
25	Afu2g02330	hypothetical protein	NaN	-0,254	0,617	0,558	0,567	0,346	0,051	-0,267	0,224	0,193	0,343	0,066	GO:0000004	unknown
25	Afu1g14990	ATP-dependent RNA helicase (Drs1), putative	-0,054	-0,915	0,661	0,640	0,606	0,167	0,102	-0,369	0,195	-0,062	0,158	-0,107	GO:0016070	RNA metabolism
25	Afu2g09080	arginine N-methyltransferase (Rmt2), putative	NaN	-0,829	0,723	0,491	0,671	0,263	-0,030	-0,501	0,357	0,072	0,321	0,151	GO:0006464	protein modification
25	Afu5g08380	hypothetical protein	-0,008	-0,916	0,389	0,528	0,835	0,543	0,037	-0,447	0,166	0,115	0,530	0,435	GO:0000004	unknown
25	Afu5g09670	RNase III domain protein	0,013	-0,987	0,268	0,660	0,703	0,375	-0,072	-0,558	0,304	0,305	0,587	0,382	GO:0000004	unknown
25	Afu1g09210	AAA family ATPase, putative	0,022	-0,846	0,348	0,644	0,489	0,116	0,012	-0,486	0,483	0,197	0,713	0,119	GO:0006412	protein biosynthesis
25	Afu1g05410	Krr1 family protein	-0,064	-0,924	0,760	0,820	0,779	0,212	-0,037	-0,421	0,569	0,135	0,606	0,267	GO:0006412	protein biosynthesis
25	Afu2g11750	mitochondrial DnaJ chaperone (Mdj1), putative	-0,003	-1,363	-0,348	0,588	0,580	0,252	0,040	-0,969	0,216	0,444	0,535	0,361	GO:0006259	DNA metabolism
25	Afu4g09960	conserved hypothetical protein	-0,025	-1,288	0,318	0,426	0,277	0,231	-0,057	-0,820	0,278	0,287	0,533	0,554	GO:0000004	unknown
25	Afu6g08890	thioesterase family protein	-0,037	-1,053	0,173	-0,008	0,235	0,214	0,083	-0,523	0,161	0,285	0,204	0,425	GO:0006629	lipid metabolism
25	Afu4g08940	hypothetical protein	0,065	-1,024	0,223	0,189	0,475	0,269	-0,030	-0,207	0,184	0,101	0,353	0,365	GO:0000004	unknown
25	Afu3g08720	hypothetical protein	0,034	-0,865	0,165	0,289	0,497	0,303	0,002	-0,360	0,095	0,385	0,466	0,379	GO:0000004	unknown
25	Afu1g10750	prolyl-tRNA synthetase	-0,111	-0,722	-0,297	0,383	0,667	0,319	-0,068	-0,669	-0,287	0,184	0,641	0,465	GO:0016070	RNA metabolism

Table S2: Distribution of the motifs HGATAR or ATCWGATAA in the regions upstream of the 49 putative SreA-regulated genes.

Gene			Motifs					
**Chromo-some**	**Locus tag**	**Description**	**strand**	**start**	**end**	**Motif consensus**	**Sequence found**	**patser score**
I	Afu1g03340	hypothetical protein	R	-1902	-1897	HGATAR	AGATAG	5,96
			D	-1618	-1613	HGATAR	TGATAA	5,96
			R	-1496	-1491	HGATAR	TGATAG	5,96
			R	-1064	-1059	HGATAR	CGATAG	5,96
			R	-278	-273	HGATAR	CGATAA	5,96
			D	-271	-266	HGATAR	AGATAA	5,96
	Afu1g03350	alpha-1,3-glucanase, putative	R	-1238	-1233	HGATAR	TGATAG	5,96
			R	-434	-426	ATCWGATAA	ATCTGATAT	8,23
			D	-264	-259	HGATAR	TGATAG	5,96
			D	-109	-104	HGATAR	TGATAA	5,96
	Afu1g03360	hypothetical protein	D	-1742	-1737	HGATAR	TGATAA	5,96
			R	-1528	-1523	HGATAR	TGATAA	5,96
			R	-1373	-1368	HGATAR	TGATAG	5,96
			D	-1206	-1198	ATCWGATAA	ATCTGATAT	8,23
			D	-399	-394	HGATAR	TGATAG	5,96
	Afu1g12240	MFS peptide transporter, putaitve	R	-1907	-1902	HGATAR	CGATAA	5,96
			D	-1885	-1880	HGATAR	AGATAG	5,96
			D	-1839	-1834	HGATAR	AGATAA	5,96
			R	-1594	-1589	HGATAR	TGATAA	5,96
			R	-1289	-1284	HGATAR	TGATAA	5,96
			D	-1264	-1259	HGATAR	AGATAG	5,96
			D	-791	-786	HGATAR	CGATAA	5,96
			R	-401	-396	HGATAR	CGATAG	5,96
			D	-249	-244	HGATAR	CGATAG	5,96
	Afu1g12690	ABC multidrug transporter Mdr4	R	-1965	-1960	HGATAR	TGATAG	5,96
			R	-1798	-1793	HGATAR	AGATAA	5,96
			R	-1712	-1707	HGATAR	TGATAA	5,96
			D	-1702	-1694	ATCWGATAA	ATCAGATTA	8,76
			D	-1694	-1689	HGATAR	AGATAA	5,96
			D	-1429	-1424	HGATAR	TGATAG	5,96
			R	-1119	-1111	ATCWGATAA	ATCTGATAA	9,95
			R	-1119	-1114	HGATAR	TGATAA	5,96
			D	-1117	-1109	ATCWGATAA	ATCAGATGA	8,48
			R	-905	-897	ATCWGATAA	ATCTGATTA	8,24
			R	-143	-138	HGATAR	CGATAA	5,96
	Afu1g12920	glycogen phosphorylase 1; possible glycogen phosphorylase	R	-1924	-1919	HGATAR	TGATAG	5,96
			R	-1895	-1890	HGATAR	AGATAA	5,96
			D	-1607	-1602	HGATAR	AGATAA	5,96
			R	-1565	-1560	HGATAR	AGATAA	5,96
			D	-1025	-1020	HGATAR	TGATAA	5,96
			R	-863	-858	HGATAR	TGATAA	5,96
			R	-740	-735	HGATAR	AGATAG	5,96
			D	-612	-607	HGATAR	TGATAA	5,96
			R	-532	-527	HGATAR	TGATAG	5,96
	Afu1g13990	conserved hypothetical protein	R	-1805	-1800	HGATAR	TGATAA	5,96
			R	-1200	-1195	HGATAR	AGATAG	5,96
			R	-995	-990	HGATAR	TGATAA	5,96
			R	-812	-807	HGATAR	AGATAG	5,96
			R	-261	-256	HGATAR	AGATAA	5,96
			R	-233	-228	HGATAR	TGATAG	5,96
			R	-118	-113	HGATAR	AGATAA	5,96
			R	-72	-67	HGATAR	CGATAA	5,96
	Afu1g17170	alpha-ketoglutarate-dependent taurine dioxygenase	D	-1704	-1699	HGATAR	AGATAA	5,96
			R	-1673	-1668	HGATAR	CGATAA	5,96
			D	-151	-146	HGATAR	TGATAG	5,96
			R	-91	-86	HGATAR	CGATAG	5,96
			R	-24	-19	HGATAR	TGATAA	5,96
	Afu1g17180	pyridine nucleotide-disulphide oxidoreductase, putative	R	-1771	-1766	HGATAR	TGATAA	5,96
			D	-1191	-1183	ATCWGATAA	ATCAGATTA	8,76
			R	-1177	-1172	HGATAR	AGATAG	5,96
			R	-1172	-1164	ATCWGATAA	ATCAGATTA	8,76
			D	-1084	-1079	HGATAR	CGATAA	5,96
			D	-747	-742	HGATAR	TGATAA	5,96
			D	-617	-612	HGATAR	AGATAA	5,96
			D	-527	-519	ATCWGATAA	ATCAGATAA	10,48
			D	-524	-519	HGATAR	AGATAA	5,96
			D	-500	-492	ATCWGATAA	ATCAGATAA	10,48
			D	-497	-492	HGATAR	AGATAA	5,96
			R	-358	-350	ATCWGATAA	ATCAGATGA	8,48
			D	-356	-348	ATCWGATAA	ATCTGATAA	9,95
			D	-353	-348	HGATAR	TGATAA	5,96

Gene			Motifs					
Chromo-some	Locus tag	Description	strand	start	end	Motif consensus	Sequence found	patser score
I	Afu1g17190	long-chain-fatty-acid-CoA ligase, putative	D	-1878	-1873	HGATAR	**CGATAG**	5,96
			D	-1835	-1830	HGATAR	**AGATAG**	5,96
			R	-1229	-1224	HGATAR	**AGATAA**	5,96
			R	-1229	-1221	ATCWGATAA	**ATCAGATAA**	10,48
			D	-1227	-1219	ATCWGATAA	**ATCTGATTA**	8,24
			R	-1216	-1208	ATCWGATAA	**ATCAGATGA**	8,48
			D	-1214	-1206	ATCWGATAA	**ATCTGATTA**	8,24
			D	-1124	-1119	HGATAR	**TGATAA**	5,96
			D	-997	-992	HGATAR	**AGATAA**	5,96
			D	-970	-965	HGATAR	**AGATAG**	5,96
			R	-907	-902	HGATAR	**TGATAG**	5,96
			D	-746	-741	HGATAR	**AGATAG**	5,96
			R	-444	-436	ATCWGATAA	**ATCTGATAA**	9,95
			R	-444	-439	HGATAR	**TGATAA**	5,96
			D	-442	-434	ATCWGATAA	**ATCAGATCA**	8,82
			R	-405	-400	HGATAR	**AGATAG**	5,96
			R	-405	-397	ATCWGATAA	**ATCAGATAG**	9,55
			D	-360	-352	ATCWGATAA	**ATCTGATAA**	9,95
			D	-357	-352	HGATAR	**TGATAA**	5,96
	Afu1g17200	nonribosomal peptide synthase, putative	R	-1376	-1368	ATCWGATAA	**ATCTGATAA**	9,95
			R	-1376	-1371	HGATAR	**TGATAA**	5,96
			D	-1331	-1323	ATCWGATAA	**ATCAGATAG**	9,55
			D	-1328	-1323	HGATAR	**AGATAG**	5,96
			R	-1294	-1286	ATCWGATAA	**ATCAGATCA**	8,82
			D	-1292	-1284	ATCWGATAA	**ATCTGATAA**	9,95
			D	-1289	-1284	HGATAR	**TGATAA**	5,96
			R	-987	-982	HGATAR	**AGATAG**	5,96
			D	-826	-821	HGATAR	**TGATAG**	5,96
			R	-763	-758	HGATAR	**AGATAG**	5,96
			R	-736	-731	HGATAR	**AGATAA**	5,96
			R	-609	-604	HGATAR	**TGATAA**	5,96
			R	-522	-514	ATCWGATAA	**ATCTGATTA**	8,24
			D	-520	-512	ATCWGATAA	**ATCAGATGA**	8,48
			R	-509	-501	ATCWGATAA	**ATCTGATTA**	8,24
			D	-507	-499	ATCWGATAA	**ATCAGATAA**	10,48
			D	-504	-499	HGATAR	**AGATAA**	5,96
	Afu1g17270	ferric-chelate reductase (Fre2), putative	D	-1090	-1085	HGATAR	**TGATAA**	5,96
			R	-836	-831	HGATAR	**AGATAG**	5,96
			D	-716	-711	HGATAR	**AGATAG**	5,96
			R	-288	-283	HGATAR	**AGATAG**	5,96
			R	-288	-280	ATCWGATAA	**ATCAGATAG**	9,55
			R	-274	-266	ATCWGATAA	**ATCTGATCA**	8,3
			D	-272	-264	ATCWGATAA	**ATCAGATTA**	8,76
			D	-264	-259	HGATAR	**AGATAA**	5,96
			R	-116	-111	HGATAR	**AGATAA**	5,96
II	Afu2g05730	siderochrome-iron transporter (MirC), putative	R	-1795	-1790	HGATAR	**CGATAA**	5,96
			D	-1456	-1451	HGATAR	**TGATAA**	5,96
			D	-1429	-1424	HGATAR	**TGATAG**	5,96
			D	-1238	-1233	HGATAR	**AGATAG**	5,96
			R	-1127	-1122	HGATAR	**AGATAA**	5,96
			R	-1113	-1108	HGATAR	**AGATAA**	5,96
			R	-970	-965	HGATAR	**TGATAA**	5,96
			R	-965	-957	ATCWGATAA	**ATCAGATAT**	8,75
			R	-686	-681	HGATAR	**CGATAA**	5,96
			D	-682	-677	HGATAR	**CGATAA**	5,96
			D	-456	-448	ATCWGATAA	**ATCAAATAA**	8,59
			D	-369	-364	HGATAR	**TGATAG**	5,96
			R	-259	-254	HGATAR	**AGATAA**	5,96
	Afu2g07680	L-ornithine N5-oxygenase SidA	R	-1856	-1851	HGATAR	**AGATAG**	5,96
			D	-1830	-1825	HGATAR	**AGATAA**	5,96
			D	-1312	-1307	HGATAR	**TGATAG**	5,96
			R	-1088	-1083	HGATAR	**TGATAG**	5,96
			R	-1023	-1018	HGATAR	**AGATAG**	5,96
			D	-200	-195	HGATAR	**TGATAG**	5,96
III	Afu3g01490	alcohol dehydrogenase, putative	D	-1675	-1670	HGATAR	**CGATAA**	5,96
			R	-1644	-1639	HGATAR	**TGATAA**	5,96
			R	-1281	-1273	ATCWGATAA	**ATCAGATCA**	8,82
			D	-1279	-1271	ATCWGATAA	**ATCTGATAC**	8,6
			D	-1125	-1120	HGATAR	**AGATAG**	5,96
			D	-975	-970	HGATAR	**TGATAA**	5,96
			D	-902	-897	HGATAR	**TGATAG**	5,96
			D	-778	-773	HGATAR	**CGATAA**	5,96
			R	-698	-693	HGATAR	**AGATAA**	5,96
	Afu3g03350	nonribosomal peptide synthase, putative	D	-881	-876	HGATAR	**TGATAA**	5,96
	Afu3g03360	hypothetical protein	D	-855	-847	ATCWGATAA	**ATCTGATAC**	8,6
			D	-630	-625	HGATAR	**TGATAA**	5,96
			R	-576	-571	HGATAR	**AGATAG**	5,96
			R	-127	-122	HGATAR	**CGATAA**	5,96

Gene			Motifs					
Chromo-some	Locus tag	Description	strand	start	end	Motif consensus	Sequence found	patser score
III	Afu3g03390	siderophore biosynthesis lipase/esterase, putative	D	-1455	-1450	HGATAR	TGATAA	5,96
			R	-1388	-1383	HGATAR	TGATAA	5,96
			R	-1379	-1371	ATCWGATAA	ATCTGATTA	8,24
			D	-1377	-1369	ATCWGATAA	ATCAGATAG	9,55
			D	-1374	-1369	HGATAR	AGATAG	5,96
			R	-1053	-1048	HGATAR	AGATAA	5,96
			R	-1053	-1045	ATCWGATAA	ATCAGATAA	10,48
			D	-918	-910	ATCWGATAA	ATCAGATAA	10,48
			D	-915	-910	HGATAR	AGATAA	5,96
			R	-803	-798	HGATAR	CGATAG	5,96
			R	-451	-443	ATCWGATAA	ATCTGATTA	8,24
			R	-417	-409	ATCWGATAA	ATCTGATTA	8,24
			D	-232	-227	HGATAR	TGATAA	5,96
			R	-135	-127	ATCWGATAA	ATCTGATAA	9,95
			R	-135	-130	HGATAR	TGATAA	5,96
	Afu3g03400	siderophore biosynthesis acetylase AceI, putative	R	-1945	-1940	HGATAR	AGATAG	5,96
			R	-1945	-1937	ATCWGATAA	ATCAGATAG	9,55
			R	-1494	-1489	HGATAR	AGATAA	5,96
			R	-1494	-1486	ATCWGATAA	ATCAGATAA	10,48
			R	-1420	-1415	HGATAR	AGATAG	5,96
			D	-1275	-1270	HGATAR	AGATAA	5,96
			D	-1000	-995	HGATAR	AGATAG	5,96
			D	-554	-549	HGATAR	AGATAA	5,96
	Afu3g03410	enoyl-CoA hydratase/isomerase family protein	R	-1879	-1874	HGATAR	AGATAG	5,96
			R	-1879	-1871	ATCWGATAA	ATCAGATAG	9,55
			D	-1877	-1869	ATCWGATAA	ATCTGATTA	8,24
			D	-1865	-1860	HGATAR	TGATAA	5,96
			R	-1798	-1793	HGATAR	TGATAA	5,96
			R	-586	-581	HGATAR	AGATAA	5,96
			D	-421	-416	HGATAR	AGATAG	5,96
	Afu3g03420	hypothetical protein	R	-1357	-1352	HGATAR	AGATAA	5,96
			R	-911	-906	HGATAR	AGATAG	5,96
			R	-636	-631	HGATAR	AGATAA	5,96
			D	-491	-486	HGATAR	AGATAG	5,96
			D	-420	-412	ATCWGATAA	ATCAGATAA	10,48
			D	-417	-412	HGATAR	AGATAA	5,96
	Afu3g03430	ABC multidrug transporter SitT, putative	D	-1196	-1191	HGATAR	AGATAA	5,96
			D	-988	-983	HGATAR	TGATAG	5,96
			R	-318	-313	HGATAR	TGATAA	5,96
			D	-245	-237	ATCWGATAA	ATCAGATAA	10,48
			D	-242	-237	HGATAR	AGATAA	5,96
	Afu3g03440	MFS family siderophore transporter, putative	D	-1300	-1295	HGATAR	TGATAA	5,96
			D	-1127	-1122	HGATAR	AGATAA	5,96
			D	-995	-990	HGATAR	TGATAG	5,96
			D	-874	-869	HGATAR	TGATAA	5,96
			D	-801	-796	HGATAR	CGATAG	5,96
			R	-541	-536	HGATAR	AGATAA	5,96
			R	-541	-533	ATCWGATAA	ATCAGATAA	10,48
			D	-465	-460	HGATAR	TGATAA	5,96
	Afu3g03640	siderochrome-iron transporter (MirB), putative	R	-1545	-1540	HGATAR	TGATAA	5,96
			R	-919	-914	HGATAR	TGATAG	5,96
			R	-435	-430	HGATAR	TGATAA	5,96
			R	-104	-99	HGATAR	AGATAA	5,96
			R	-104	-96	ATCWGATAA	ATCAGATAA	10,48
			D	-102	-94	ATCWGATAA	ATCTGATCA	8,3
			D	-35	-30	HGATAR	AGATAA	5,96
	Afu3g03650	acetyltransferase, GNAT family, putative	R	-844	-839	HGATAR	AGATAA	5,96
			R	-839	-834	HGATAR	AGATAA	5,96
			R	-839	-831	ATCWGATAA	ATCAGATAA	10,48
			R	-196	-191	HGATAR	TGATAA	5,96
	Afu3g03660	esterase superfamily protein	R	-1466	-1461	HGATAR	CGATAG	5,96
			R	-683	-678	HGATAR	AGATAG	5,96
			D	-645	-637	ATCWGATAA	ATCAAATAA	8,59
			R	-428	-423	HGATAR	TGATAG	5,96
			D	-366	-361	HGATAR	TGATAA	5,96
			D	-357	-352	HGATAR	TGATAA	5,96
			R	-346	-338	ATCWGATAA	ATCTGATCA	8,3
			D	-344	-336	ATCWGATAA	ATCAGATTA	8,76
	Afu3g03670	ABC multidrug transporter, putative	D	-804	-796	ATCWGATAA	ATCAGATAC	9,13
			R	-386	-378	ATCWGATAA	ATCAGATTA	8,76
			D	-384	-376	ATCWGATAA	ATCTGATCA	8,3
			R	-370	-365	HGATAR	TGATAA	5,96
			R	-361	-356	HGATAR	TGATAA	5,96
			D	-299	-294	HGATAR	TGATAG	5,96
			R	-85	-77	ATCWGATAA	ATCAAATAA	8,59
			D	-44	-39	HGATAR	AGATAG	5,96
	Afu3g07410	isoamyl alcohol oxidase	D	-1901	-1896	HGATAR	TGATAA	5,96
			R	-1502	-1497	HGATAR	CGATAG	5,96
			D	-1331	-1326	HGATAR	CGATAG	5,96
			R	-592	-587	HGATAR	TGATAG	5,96
			R	-511	-506	HGATAR	TGATAA	5,96
			R	-308	-303	HGATAR	TGATAA	5,96
			D	-289	-284	HGATAR	TGATAG	5,96
	Afu3g13500	hypothetical protein	D	-1874	-1869	HGATAR	CGATAG	5,96
			D	-1649	-1641	ATCWGATAA	ATCTGATAC	8,6
			D	-1575	-1570	HGATAR	TGATAA	5,96
			D	-990	-985	HGATAR	TGATAG	5,96
			R	-483	-478	HGATAR	CGATAG	5,96
			D	-306	-301	HGATAR	TGATAA	5,96
			R	-122	-117	HGATAR	AGATAG	5,96

Gene			Motifs					
Chromo-some	Locus tag	Description	strand	start	end	Motif consensus	Sequence found	patser score
IV	Afu4g10100	FAD binding domain protein	R	-1753	-1748	HGATAR	AGATAA	5,96
			D	-1747	-1742	HGATAR	AGATAG	5,96
			R	-1702	-1694	ATCWGATAA	ATCTGATAA	9,95
			R	-1702	-1697	HGATAR	TGATAA	5,96
			D	-1700	-1692	ATCWGATAA	ATCAGATGA	8,48
			R	-1639	-1634	HGATAR	AGATAG	5,96
			R	-1639	-1631	ATCWGATAA	ATCAGATAG	9,55
			R	-1524	-1516	ATCWGATAA	ATCAGATAT	8,75
			D	-1422	-1414	ATCWGATAA	ATCAGATAA	10,48
			D	-1419	-1414	HGATAR	AGATAA	5,96
			D	-1192	-1184	ATCWGATAA	ATCAGATTA	8,76
			R	-1071	-1066	HGATAR	CGATAA	5,96
			D	-886	-878	ATCWGATAA	ATCAGATAA	10,48
			D	-883	-878	HGATAR	AGATAA	5,96
			D	-869	-864	HGATAR	TGATAA	5,96
			R	-685	-677	ATCWGATAA	ATCAGATTA	8,76
			R	-527	-522	HGATAR	AGATAG	5,96
			R	-527	-519	ATCWGATAA	ATCAGATAG	9,55
			R	-300	-295	HGATAR	TGATAG	5,96
	Afu4g14070	glycosyl transferase, putative	R	-1926	-1921	HGATAR	CGATAG	5,96
			R	-1907	-1902	HGATAR	TGATAG	5,96
			D	-1863	-1858	HGATAR	TGATAG	5,96
			R	-1770	-1765	HGATAR	TGATAG	5,96
			D	-348	-343	HGATAR	TGATAA	5,96
			R	-283	-278	HGATAR	CGATAG	5,96
			R	-170	-165	HGATAR	TGATAG	5,96
			R	-33	-28	HGATAR	CGATAA	5,96
V	Afu5g00710	GABA permease, putative	D	-715	-710	HGATAR	CGATAA	5,96
			R	-393	-388	HGATAR	CGATAG	5,96
			R	-42	-37	HGATAR	AGATAA	5,96
	Afu5g02700	multidrug resistant protein	D	-1128	-1123	HGATAR	CGATAG	5,96
			D	-1029	-1024	HGATAR	TGATAA	5,96
			D	-791	-786	HGATAR	TGATAG	5,96
			R	-749	-744	HGATAR	TGATAG	5,96
			R	-569	-564	HGATAR	TGATAG	5,96
			D	-393	-388	HGATAR	AGATAA	5,96
			D	-139	-134	HGATAR	CGATAA	5,96
	Afu5g03780	L-PSP endoribonuclease family protein (Brt1), putative	R	-1842	-1837	HGATAR	AGATAA	5,96
			D	-1773	-1768	HGATAR	TGATAA	5,96
			D	-1719	-1714	HGATAR	TGATAG	5,96
			D	-1414	-1409	HGATAR	CGATAG	5,96
			D	-182	-177	HGATAR	TGATAG	5,96
			D	-178	-173	HGATAR	AGATAG	5,96
	Afu5g03790	ferro-O2-oxidoreductase	D	-1521	-1516	HGATAR	TGATAA	5,96
			D	-1464	-1459	HGATAR	AGATAA	5,96
			D	-1212	-1207	HGATAR	CGATAA	5,96
			D	-1181	-1176	HGATAR	TGATAA	5,96
			R	-1173	-1168	HGATAR	AGATAA	5,96
			D	-1116	-1111	HGATAR	TGATAA	5,96
			R	-1073	-1068	HGATAR	AGATAA	5,96
			R	-1073	-1065	ATCWGATAA	ATCAGATAA	10,48
			D	-1064	-1059	HGATAR	TGATAA	5,96
			D	-861	-856	HGATAR	AGATAG	5,96
			D	-645	-640	HGATAR	TGATAA	5,96
			R	-135	-130	HGATAR	TGATAA	5,96
	Afu5g03800	high-affinity iron permease CaFTR2	D	-1220	-1215	HGATAR	TGATAA	5,96
			R	-710	-705	HGATAR	TGATAA	5,96
			R	-494	-489	HGATAR	AGATAG	5,96
			R	-291	-286	HGATAR	TGATAA	5,96
			D	-285	-277	ATCWGATAA	ATCAGATAA	10,48
			D	-282	-277	HGATAR	AGATAA	5,96
			R	-239	-234	HGATAR	TGATAA	5,96
			D	-182	-177	HGATAR	AGATAA	5,96
			R	-174	-169	HGATAR	TGATAA	5,96
			R	-143	-138	HGATAR	CGATAA	5,96
	Afu5g03920	bZIP transcription factor (HapX), putative	R	-1867	-1862	HGATAR	AGATAG	5,96
			D	-1604	-1599	HGATAR	TGATAG	5,96
			D	-1535	-1530	HGATAR	TGATAA	5,96
			D	-1350	-1342	ATCWGATAA	ATCAGATAA	10,48
			D	-1347	-1342	HGATAR	AGATAA	5,96
			D	-1340	-1332	ATCWGATAA	ATCAGATGA	8,48
			R	-1214	-1206	ATCWGATAA	ATCTGATAA	9,95
			R	-1214	-1209	HGATAR	TGATAA	5,96
			D	-837	-829	ATCWGATAA	ATCTGATAA	9,95
			D	-834	-829	HGATAR	TGATAA	5,96
			D	-732	-727	HGATAR	AGATAA	5,96
			R	-675	-670	HGATAR	TGATAA	5,96
			D	-464	-459	HGATAR	CGATAA	5,96
			R	-456	-448	ATCWGATAA	ATCAGATTA	8,76
			R	-395	-390	HGATAR	AGATAG	5,96
	Afu5g03930	alcohol dehydrogenase, putative	R	-1773	-1768	HGATAR	TGATAG	5,96
			D	-1454	-1449	HGATAR	AGATAG	5,96
			R	-1064	-1059	HGATAR	TGATAA	5,96
			D	-807	-802	HGATAR	AGATAA	5,96
			D	-689	-684	HGATAR	TGATAA	5,96
			D	-15	-10	HGATAR	TGATAA	5,96
	Afu5g13300	aspartic endopeptidase Pep1	R	-1532	-1527	HGATAR	AGATAA	5,96
			R	-1155	-1147	ATCWGATAA	ATCAGATAT	8,75
			R	-954	-949	HGATAR	TGATAG	5,96
			R	-390	-385	HGATAR	AGATAG	5,96
			D	-379	-374	HGATAR	CGATAA	5,96
			R	-174	-169	HGATAR	AGATAG	5,96

Gene			Motifs					
Chromo-some	Locus tag	Description	strand	start	end	Motif consensus	Sequence found	patser score
VI	Afu6g03680	hypothetical protein	R	-1719	-1714	HGATAR	**AGATAA**	5,96
			R	-1596	-1591	HGATAR	**TGATAA**	5,96
			R	-1541	-1536	HGATAR	**CGATAA**	5,96
			R	-1533	-1528	HGATAR	**AGATAA**	5,96
			D	-1382	-1377	HGATAR	**AGATAG**	5,96
			R	-1139	-1134	HGATAR	**AGATAG**	5,96
			D	-1137	-1129	ATCWGATAA	**ATCTAATAA**	8,07
			R	-1066	-1061	HGATAR	**AGATAA**	5,96
			R	-347	-342	HGATAR	**AGATAG**	5,96
	Afu6g04920	NAD-dependent formate dehydrogenase	R	-1291	-1286	HGATAR	**AGATAG**	5,96
			D	-821	-816	HGATAR	**AGATAA**	5,96
			R	-214	-209	HGATAR	**AGATAG**	5,96
	Afu6g13590	3-isopropylmalate dehydrogenase	R	-1459	-1454	HGATAR	**CGATAA**	5,96
			R	-912	-907	HGATAR	**AGATAG**	5,96
			D	-608	-603	HGATAR	**AGATAA**	5,96
			D	-458	-453	HGATAR	**TGATAG**	5,96
VII	Afu7g02330	hypothetical protein	R	-1787	-1782	HGATAR	**AGATAG**	5,96
			R	-1700	-1695	HGATAR	**CGATAA**	5,96
			D	-778	-773	HGATAR	**TGATAA**	5,96
			D	-725	-720	HGATAR	**TGATAA**	5,96
			D	-628	-620	ATCWGATAA	**ATCTGATAG**	9,02
			D	-625	-620	HGATAR	**TGATAG**	5,96
			R	-557	-552	HGATAR	**TGATAG**	5,96
			R	-317	-312	HGATAR	**TGATAA**	5,96
			D	-12	-4	ATCWGATAA	**ATCAGATAA**	10,48
			D	-9	-4	HGATAR	**AGATAA**	5,96
	Afu7g04730	siderochrome-iron transporter, putative	D	-1972	-1967	HGATAR	**AGATAG**	5,96
			D	-1520	-1515	HGATAR	**AGATAG**	5,96
			D	-1475	-1467	ATCWGATAA	**ATCAGATTA**	8,76
			D	-1462	-1457	HGATAR	**AGATAA**	5,96
			R	-1332	-1327	HGATAR	**AGATAA**	5,96
			D	-1237	-1229	ATCWGATAA	**ATCAGATAA**	10,48
			D	-1234	-1229	HGATAR	**AGATAA**	5,96
			R	-1225	-1217	ATCWGATAA	**ATCTGATCA**	8,3
			D	-1223	-1215	ATCWGATAA	**ATCAGATAA**	10,48
			D	-1220	-1215	HGATAR	**AGATAA**	5,96
			R	-1006	-1001	HGATAR	**TGATAG**	5,96
			R	-856	-851	HGATAR	**AGATAG**	5,96
			R	-814	-809	HGATAR	**AGATAG**	5,96
			R	-487	-482	HGATAR	**AGATAA**	5,96
			R	-487	-479	ATCWGATAA	**ATCAGATAA**	10,48
			R	-459	-454	HGATAR	**AGATAG**	5,96
			R	-459	-451	ATCWGATAA	**ATCAGATAG**	9,55
			D	-457	-449	ATCWGATAA	**ATCTGATCA**	8,3
	Afu7g06060	siderochrome-iron transporter (Sit1), putative	D	-1437	-1429	ATCWGATAA	**ATCCGATAA**	9,19
			D	-1434	-1429	HGATAR	**CGATAA**	5,96
			R	-1181	-1176	HGATAR	**TGATAG**	5,96
			R	-1040	-1035	HGATAR	**AGATAG**	5,96
			R	-454	-446	ATCWGATAA	**ATCAGATAC**	9,13
			R	-435	-430	HGATAR	**AGATAA**	5,96
			R	-435	-427	ATCWGATAA	**ATCAGATAA**	10,48
			R	-380	-375	HGATAR	**AGATAA**	5,96
			R	-380	-372	ATCWGATAA	**ATCAGATAA**	10,48
			D	-378	-370	ATCWGATAA	**ATCTGATCA**	8,3
			R	-269	-264	HGATAR	**CGATAG**	5,96
			D	-180	-172	ATCWGATAA	**ATCAGATAA**	10,48
			D	-177	-172	HGATAR	**AGATAA**	5,96
	Afu7g06080	ubiE/COQ5 methyltransferase, putative	R	-1051	-1046	HGATAR	**AGATAG**	5,96
			D	-234	-229	HGATAR	**TGATAG**	5,96
	Afu7g06130	integral membrane protein	R	-532	-527	HGATAR	**CGATAG**	5,96
			D	-189	-184	HGATAR	**TGATAA**	5,96
VIII	Afu8g02750	nucleolar protein CgrA	D	-1424	-1419	HGATAR	**TGATAG**	5,96
			D	-1147	-1142	HGATAR	**TGATAG**	5,96
			D	-986	-981	HGATAR	**CGATAG**	5,96
			R	-478	-473	HGATAR	**AGATAA**	5,96
			R	-233	-228	HGATAR	**AGATAA**	5,96
			R	-233	-225	ATCWGATAA	**ATCAGATAA**	10,48
			D	-217	-212	HGATAR	**AGATAA**	5,96
	Afu8g02760	mitochondrial ornithine carrier protein (AmcA), putative	R	-1996	-1991	HGATAR	**CGATAA**	5,96
			D	-1455	-1450	HGATAR	**AGATAG**	5,96
			D	-815	-810	HGATAR	**CGATAG**	5,96
			R	-451	-446	HGATAR	**AGATAA**	5,96
			D	-438	-430	ATCWGATAA	**ATCAGATAA**	10,48
			D	-435	-430	HGATAR	**AGATAA**	5,96
			D	-190	-185	HGATAR	**AGATAA**	5,96

Table S3: Two motifs enriched in the region upstream of the 49 genes that may be regulated by SreA in *A. fumigatus*

	Average number of motifs			
	HGATAR		ATCWGATAA	
**Chromo-some**	**Chromosome average**	**putative SreA-regulated genes**	**Chromosome average**	**putative SreA-regulated genes**
I	5,71	7,08	0,37	2,58
II	5,57	8,50	0,33	1,00
III	5,64	5,07	0,37	1,87
IV	5,62	9,00	0,33	4,50
V	5,80	7,13	0,38	1,00
VI	5,71	5,00	0,35	0,33
VII	5,54	6,00	0,43	3,00
VIII	5,74	6,00	0,39	1,00
Average	5,67	6,33	0,36	1,96

Cutoff scores for patser in RSAT were 5, and 8 for HGATAR and ATCWGATAA, respectively.

Table S4. Hybridization probes and oligonucleotides used in this study

Hybridisation probe	Primer	Sequence 5' - 3'
	oAfsreA1	AACCCGCATGTCTAAGCC
	oAfsreA2	CAC GCA GCA CTG AAT CAC
sreA	oSreA-f	CTCAGTACGATCGCTTCC
	oSreA-r	GTTGGACGAGTAGGTAGC
sidA	oSidA-f	AACTACCTCCACCAGAAG
	oSidA-r	GAACGGCAATGTTGTAAG
ftrA	oFrA-f	GGGACAAGAGCAAGATGC
	oFtrA-r	CCCAGTAGAGGATGCAAG
fetC	oFetC-f	GTGACCGATCCCAAGAAC
	oFetC-r	GGATGGGAATGTCTTGTG
cat1	oCat1-f	TTCTATACCGACGAGGGC
	oCat1-r	GGTTGGCTTGTTTTGGGG
cccA	oAfccc1-1.f	GAT TCC GAC ACC CTA GAC
	oAfccc1-1.r	GCG ATG ATG TTG TCC CTG
cycA	oCytC-f	CCCTTTCTTGCAGTGTCC
	oCytC-r	CCGCGCATCTGCTTTTAC
tubA	oTubfum1	ATATGTTCCTCGTGCCGTTC
	oTUBFUMr	CCTTACCACGGAAAATGGCA

Table S5. Genes with proven or likely function in iron metabolism used for promoter motif studies

Gene	Putative function
Afu1g12690	ABC multidrug transporter Mdr4
Afu1g17180	pyridine nucleotide-disulphide oxidoreductase
Afu1g17190, *sidI*	long-chain-fatty-acid-CoA ligase
Afu1g17200, *sidC*	ferricrocin NRPS
Afu1g17270, *freB*	ferric-chelate reductase
Afu2g05730	siderophore iron transporter
Afu2g07680, *sidA*	L-ornithine N5-oxygenase SidA
Afu3g03390,	siderophore biosynthesis lipase/esterase
Afu3g03400, *sidF*	hydroxyornithine transacylase
Afu3g03410, *sidH*	enoyl-CoA hydratase/isomerase
Afu3g03420, *sidD*	fusarinine C NRPS
Afu3g03430	ABC multidrug transporter
Afu3g03440	siderophore iron transporter
Afu3g03640	siderophore iron transporter
Afu3g03650, *sidG*	fusarinine C acetyltransferase
Afu3g03660, *estB*	TafC esterase
Afu3g03670, *abcB*	ABC multidrug transporter
Afu5g03790, *fetC*	ferroxidase
Afu5g03800, *ftrA*	high-affinity iron permease
Afu5g03920, *hapX*	bZIP transcription factor
Afu7g04730	siderophore iron transporter
Afu7g06060	siderophore iron transporter

```
                      10        20        30        40        50        60
                       |         |         |         |         |         |
Af-SreA      -CSNCGTKSTPLWRRSPTGAMICNACGLYLKARNVARPTKRNRMEPGSEAGGQQTGHGIA
An-SreA      -CSNCGTKSTPLWRRSPTGAMICNACGLYLKARNVARPTKRNRTQASPEAYHPQNQSVGS
Ca-Sfu1      -CSNCGTTKTPLWRRAPDGTLICNACGLYYRSNNTHRPVN----LK-----RPPNTIA–
Um-Urbs1     -CSNCGVTSTPLWRRAPDGSTICNACGLYIKSHSTHRSASNR--LSGSDA-SPPTHEAKL
Cn-Cir1                                              NASPLP-----SPPPSTAVT
              *****...******:* *: ******** ::... *...

                      70        80        90       100       110       120
                       |         |         |         |         |         |
Af-SreA      QSESASQGHCQSNSGGGSCPGGGNCNGTGGAEGCDGCPAYNNRVYKSAARGTVPIHSWRA
An-SreA      QPDPAVTG---SEGCTGSCPGGGNCNGTGGAEGCDGCPAYNNRVYKSTARGNVAAHALNR
Ca-Sfu1      -VV------KEEE---GSCKGDGRCNGTGGSAACKGCPAYNNRIVAKKALEKSPKNDSSR
Um-Urbs1     AAAGPSCS-REDDPKSGSCPGDGLCNGTGGTASCSGCPAYNNNLSHALKVSKRESQTSED
Cn-Cir1      ASAHPS---------AGTCPGDGRCNGAGGKAGCEGCPTYNNSIASGLVSASNSHAASHP
                             *:* *.* ***:**  .*.***:*** :       .

                     130       140       150       160       170       180
                       |         |         |         |         |         |
Af-SreA      TNSGSDRPLA--------------------------------------------------
An-SreA      AGNSDAVPSP--------------------------------------------------
Ca-Sfu1      APIDKSLKRS--------------------------------------------------
Um-Urbs1     PPPARTAERAPP------------------------------------------------
Cn-Cir1      PNVSEGIERPVRNIYDREHRPYGFDRFMENSMGNGLAPRALPRQSPDQRQAHPSPVTTQP
             .        .

                     190       200       210       220       230       240
                       |         |         |         |         |         |
Af-SreA      --------------------ETENDAGKNGATVEGGNMLVSCQNCGTTVTPLWRRDENGH
An-SreA      --------------------EAEAPARNSGQPE--GNMLVACQNCGTTVTPLWRRDENGH
Ca-Sfu1      --------------------TSTDATTEDESSL-----AIACFNCGTTITPLWRRDDAGN
Um-Urbs1     --------------------VAEEKMDDDKSVVG----ALRCTNCQTTTTPLWRRDEDGN
Cn-Cir1      LMHPTSEKGTPTRFSPDSDVETPAAPGGNGSGLAATPVGMSCRNCGTSTTPLWRRDEEGR
                                  :      .          : * ** *: *******: *.

                     250       260       270
                       |         |         |
Af-SreA      PICNACGLYYKLHGCYRPTTMKKSIIKRRK
An-SreA      PICNACGLYYKLHGSYRPTTMKKTIIKRRK
Ca-Sfu1      TICNACGLFYRLHGSHRPIKMKRPTIKRRK
Um-Urbs1     NICNACGLYHKLHGTHRPIGMKKSVIKRRK
Cn-Cir1      PQCNACGLYHKLHGVPRPVAMKKTVIKRRK
               ******:::***  **  **:. *****
```

Fig. S1. Multiple alignment (CLUSTALW) of the region encompassing the two GATA-type zinc fingers and the cystein-rich region (boxed in yellow) of SreA orthologs from *A. fumigatus* (Af), *A. nidulans* (An), *C. albicans* (Ca), *U. maydis* (Um), and *C. neoformans* (Cn). Identical amino acids are coloured in red, conserved amino acids in blue and green, respectively. The analysis was carried out at http://npsa-pbil.ibcp.fr/cgi-bin/npsa_automat.pl?page=npsa_clustalw.html.

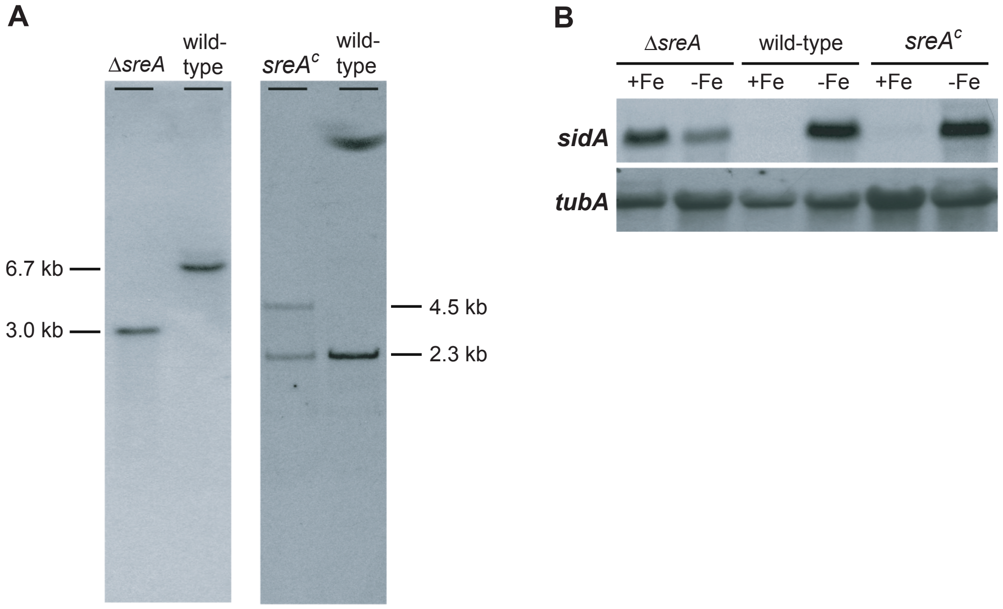

Fig. S2. Southern (A) and Northern (B) blot analysis of *A. fumigatus* wild-type, Δ*sreA* and *sreA*^{c} strains. (A) Southern blot analysis of *Nde*I- or *Xba*I–digested genomic DNA of wild-type, Δ*sreA* and *sreA*^{c}. (B) For Northern analysis, total RNA of *A. fumigatus* wild-type, Δ*sreA* and *sreA*^{c} strains was isolated after growth for 24 h under iron-depleted (-Fe) and iron-replete (+Fe) conditions. Blots were hybridised with the *sidA*- and *tubB*-specific probes. Expression of *sidA*, which is deregulated in Δ*sreA*, is wild-type-like expressed in *sreA*^{c}.

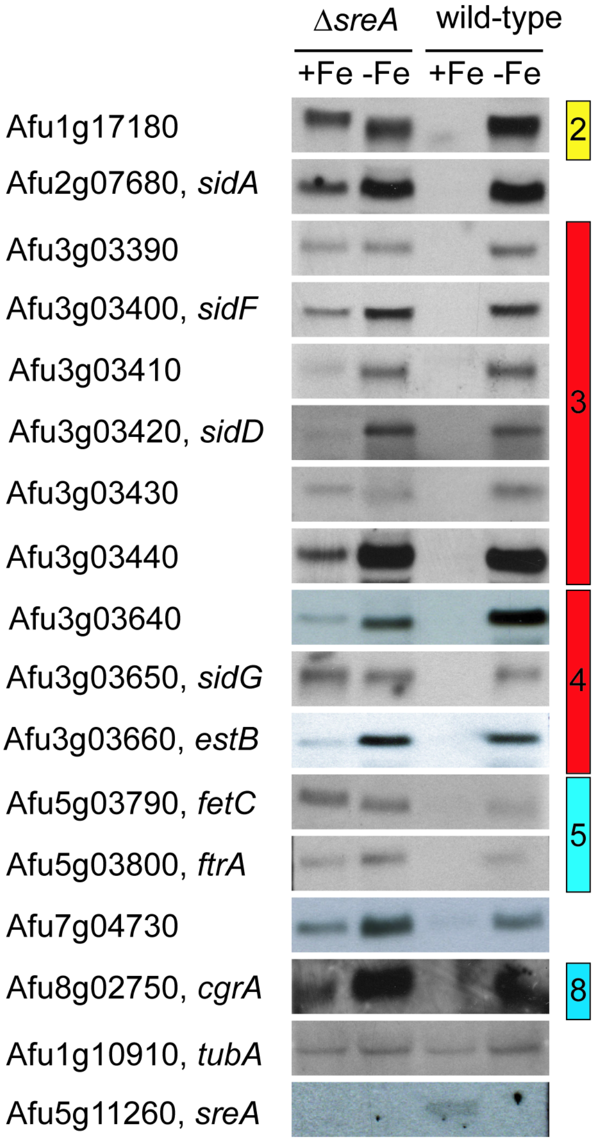

Fig. S3. Northern blot analysis of wild-type and Δ*sreA* strains during steady state iron-replete (+Fe) and depleted growth (-Fe). Northern blot analysis was performed as described in Materials and Methods using wild-type and Δ*sreA* RNA isolated after growth for 24 h under iron-depleted (-Fe) and iron-replete (+Fe) conditions. As a control for RNA quality, blots were hybridized with beta-tubulin encoding *tubA*. Numbers and color-coded boxes, representing clustered genes refer to Fig. 5.

Fig. S4. Gene expression in response to iron concentration after a shift from iron depleted (-Fe) to iron-replete (10 μM $FeSO_4$) conditions. A total of 1,147 genes that were differentially expressed (95% confidence level) in the wild-type strain in response to the shift at one or more time points were grouped using *k*-means clustering (*k*= 25; Euclidean distance). Genes in each cluster were grouped again using hierarchical clustering (Euclidean distance; average linkage clustering) based on similar expression patterns. The large bar in the box indicates colors corresponding to the range of the observed expression ratios on a $\log_2$ scale, while the two small bars below indicate the time-course data from the wild-type and *ΔsreA* strains.

A. Genes down-expressed. Clusters 1-13 showing down-expression in response to the iron-replete condition are displayed. Clusters 1-3 that contain putative *sreA*-regulated genes are boxed.

B. Genes up-expressed. Clusters 14-18 showing up-expression in response to the iron-replete condition are displayed.

C. Genes of up and down expressions. Clusters 19-25 showing both up and down expression in response to the iron-replete condition are displayed.

Figures: see next three pages.

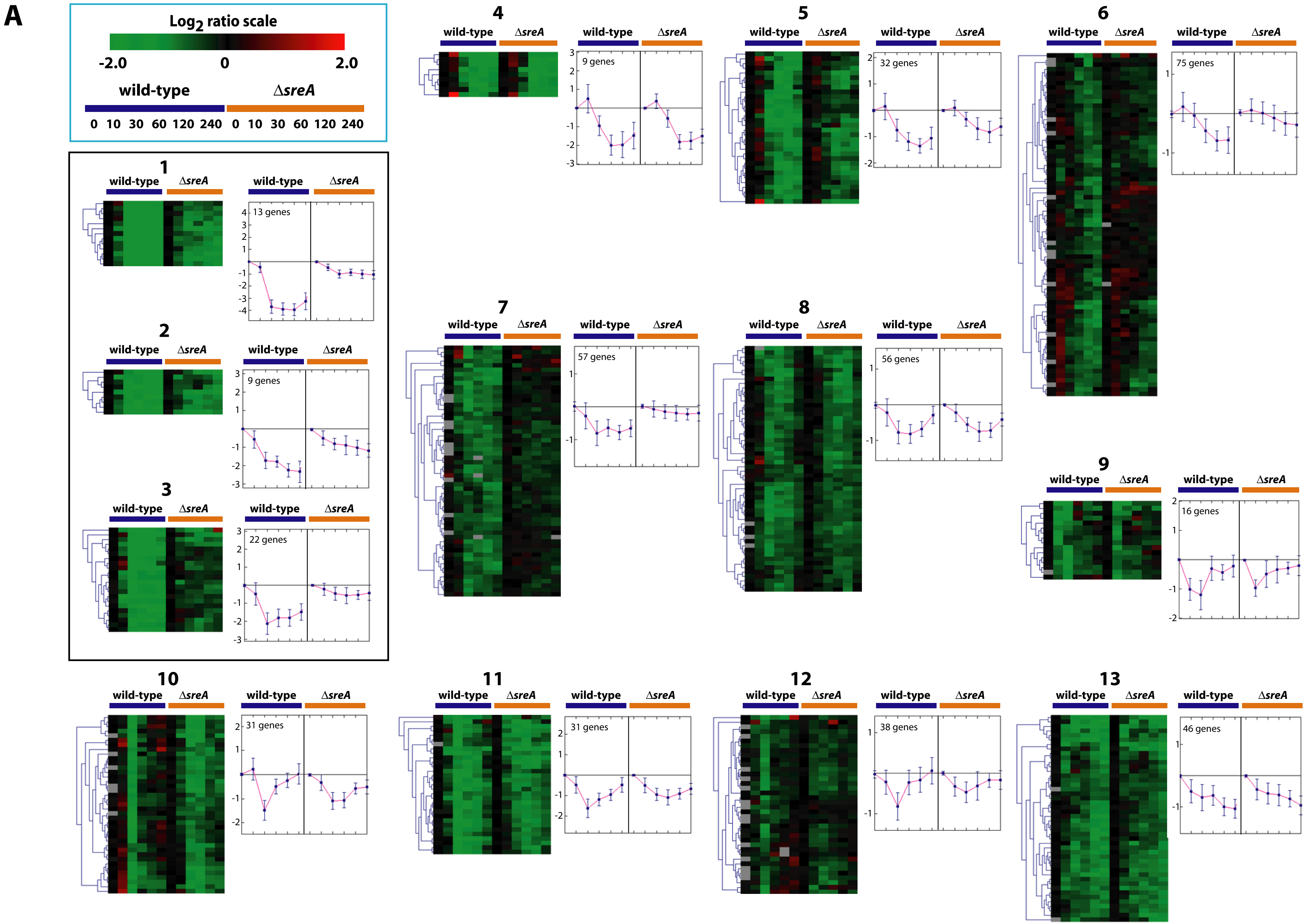
A
Log2 ratio scale
-2.0
0
2.0
wild-type
ΔsreA
0 10 30 60 120 240 0 10 30 60 120 240
1
13 genes
2
9 genes
3
22 genes
4
9 genes
5
32 genes
6
75 genes
7
57 genes
8
56 genes
9
16 genes
10
31 genes
11
31 genes
12
38 genes
13
46 genes

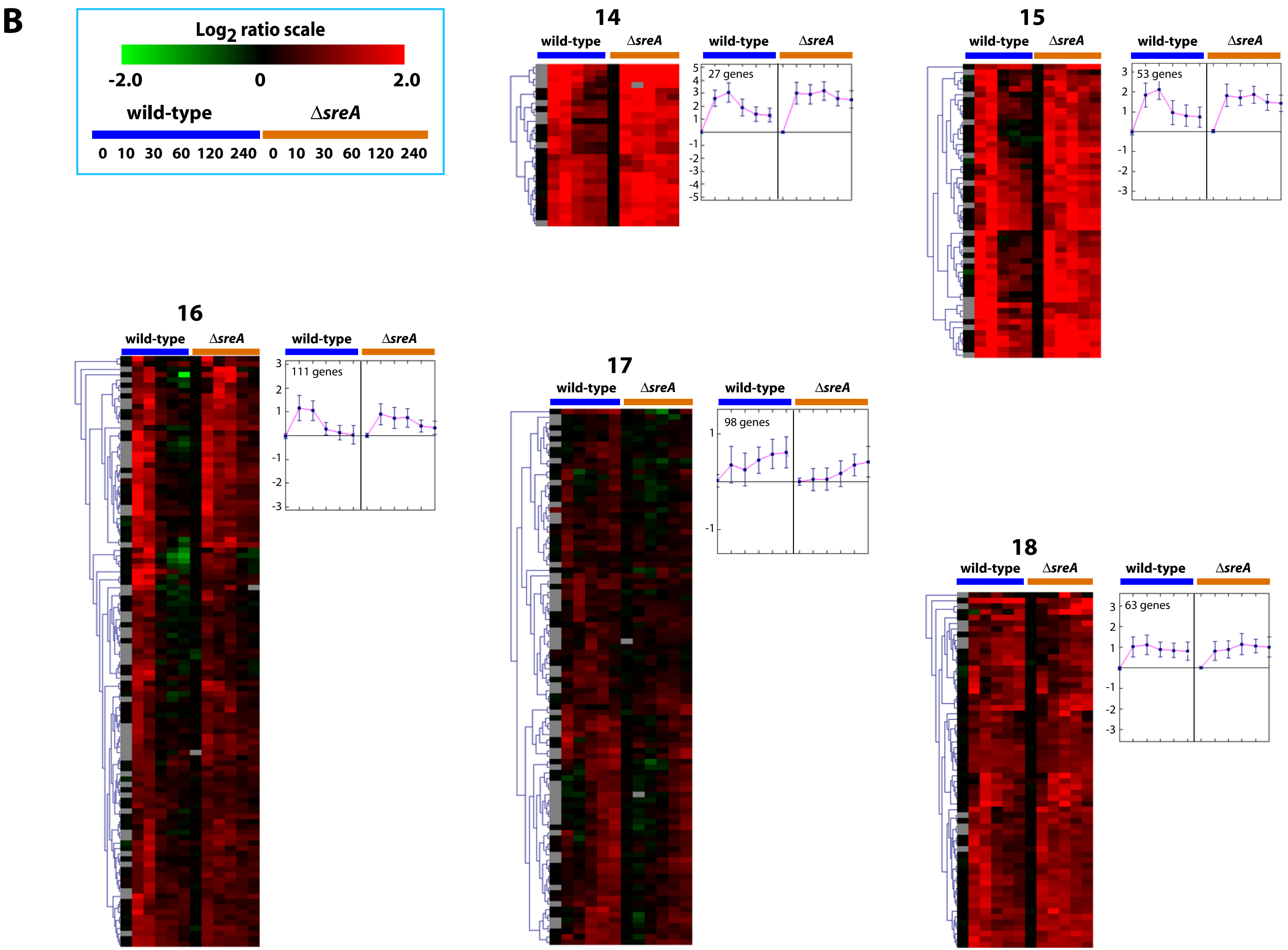

B
Log2 ratio scale
-2.0
0
2.0
wild-type
ΔsreA
0 10 30 60 120 240 0 10 30 60 120 240
14
wild-type
ΔsreA
27 genes
5
4
3
2
1
-1
-2
-3
-4
-5
15
wild-type
ΔsreA
53 genes
3
2
1
-1
-2
-3
16
wild-type
ΔsreA
111 genes
17
wild-type
ΔsreA
98 genes
1
-1
18
wild-type
ΔsreA
63 genes

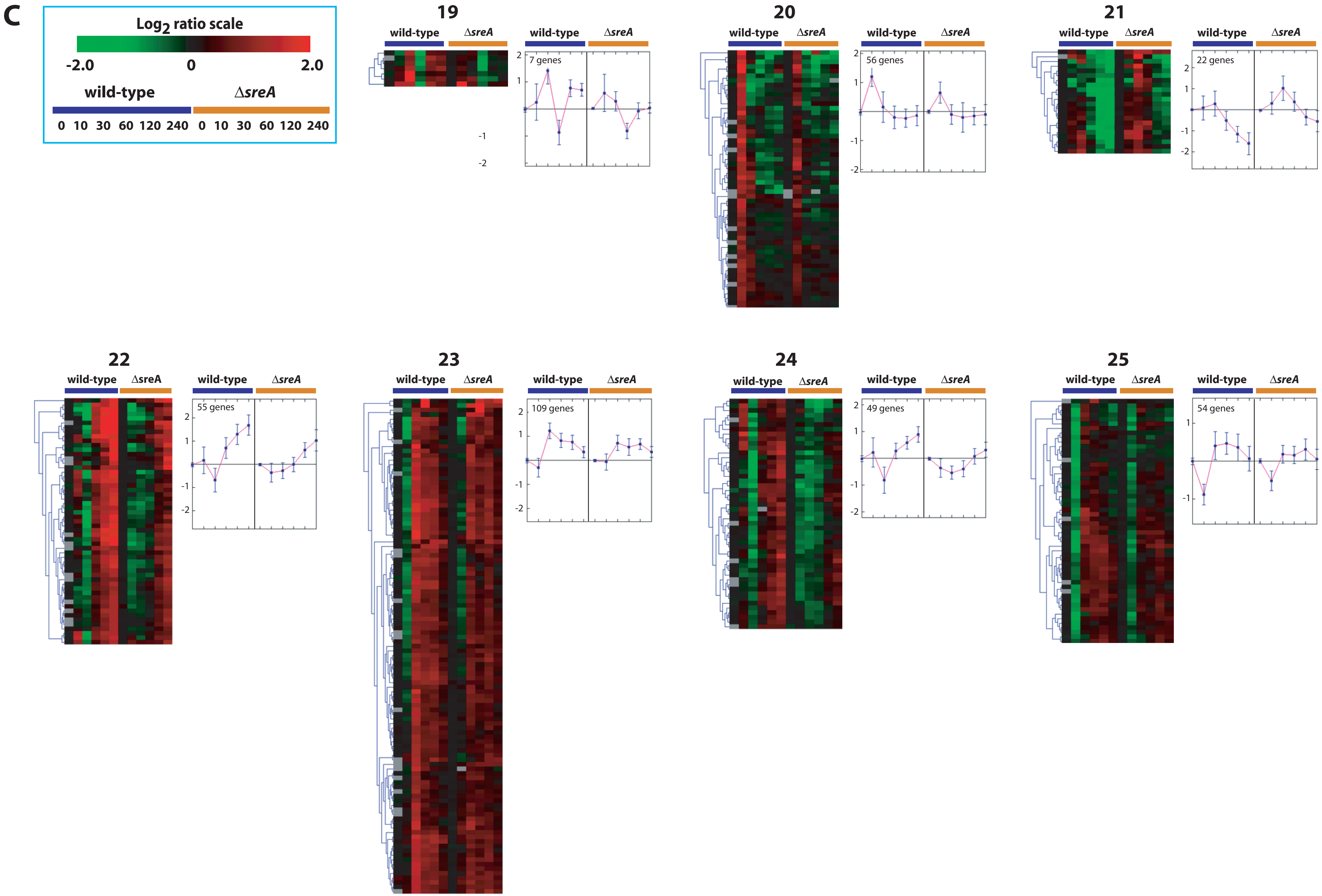
C
Log2 ratio scale
-2.0
0
2.0
wild-type
ΔsreA
0 10 30 60 120 240 0 10 30 60 120 240
19
20
21
22
23
24
25
wild-type
ΔsreA
7 genes
56 genes
22 genes
55 genes
109 genes
49 genes
54 genes

ATCwgAtaa

**A**	135	0	0	66	16	135	0	90	74
**C**	0	0	135	18	7	0	0	17	19
**G**	0	0	0	12	107	0	0	12	29
**T**	0	135	0	39	5	0	135	16	13

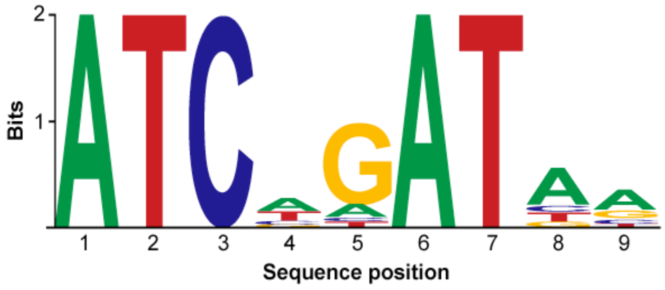

HGATAR

**A**	2	6	0	0	6	3
**C**	2	0	0	0	0	0
**G**	0	0	6	0	0	3
**T**	2	0	0	6	0	0

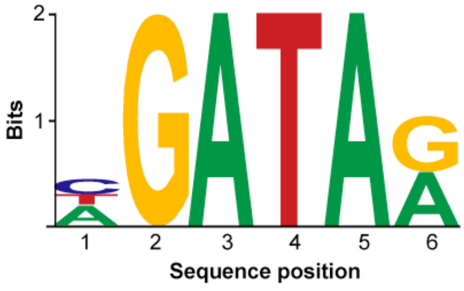

Fig. S5. Matrices used in the motif analyses.
